# Risk Factors Associated With Incidence of Lung Cancer in Never-Smokers: A Systematic Review and Meta-Analysis

**DOI:** 10.1016/j.jtocrr.2025.100910

**Published:** 2025-09-18

**Authors:** Sindhu Bhaarrati Naidu, Allegra Wisking, Akul Karoshi, Sarah Burdett, Peter J. Godolphin, Sanjay Popat, OLIVE PPI Group, Sam M. Janes, Neal Navani

**Affiliations:** a Lungs for Living Research Centre, UCL Respiratory, University College London, London, United Kingdom; b University College London, London, United Kingdom; c Meta-analysis Group, MRC Clinical Trials Unit at UCL, Institute of Clinical Trials and Methodology, UCL, London, United Kingdom; d Lung Unit, Royal Marsden Hospital, London, United Kingdom; e Division of Clinical Studies, Institute of Cancer Research, London, United Kingdom

**Keywords:** Lung neoplasms, Never-smokers, Nonsmokers, Risk factors, Epidemiology

## Abstract

**Objectives:**

Lung cancer is the leading cause of cancer mortality globally. Although often associated with smoking, up to 25% of cases worldwide and 50% in East Asia occur in “never-smokers.” There are currently no robust tools for predicting lung cancer in individuals who have never smoked (LCINS) for populations outside East Asia.

Together with a group of patient representatives, the authors of this study aimed to summarise risk factors for LCINS and quantify risk in different geographical regions.

**Methods:**

This study was prospectively registered (PROSPERO-CRD42022379253). The systematic review and meta-analysis included studies published from 2017 and aimed to comprehensively investigate risk factors associated with LCINS incidence. Risk of bias was assessed using Newcastle-Ottawa Scale.

**Results:**

A total of 6725 reports were identified and 54 studies were included, with multivariable analysis of 192 factors in 16 million never-smokers. No studies were assessed as having high risk of bias. Of the participants, 8,241,269 (51.0%) were from Western countries.

The meta-analysis found that female sex (adjusted hazard ratio [aHR] 1.28 [95% confidence interval or CI 1.12–1.47]), previous cancer (aHR 2.04 [1.95–2.13]), rheumatoid arthritis (aHR 1.41 [1.15–1.73]), passive smoking (aHR 1.30 [1.22–1.40]), PM10 (aHR 1.10 [1.09–1.11]), and PM2.5 (aHR 1.16 [1.03–1.30]) pollution were associated with LCINS. In planned subgroup analyses by region, LCINS was associated with family history of lung cancer in East Asian (aHR 1.56 [1.23–1.98]) but not Western countries (aHR 0.86 [0.35–2.11]).

**Conclusion:**

We found key factors linked with LCINS, including female sex, rheumatoid arthritis, and pollution and, for the first time, quantified their association through meta-analyses of studies globally. This may be used to develop tools to detect LCINS earlier.

## Graphical abstract

Together with a group of patient representatives, the authors in this study designed an infographic to highlight key factors associated with LCINS as identified in the systematic review and meta-analysis that will be of interest to health care professionals and members of the public.

**Figure:**
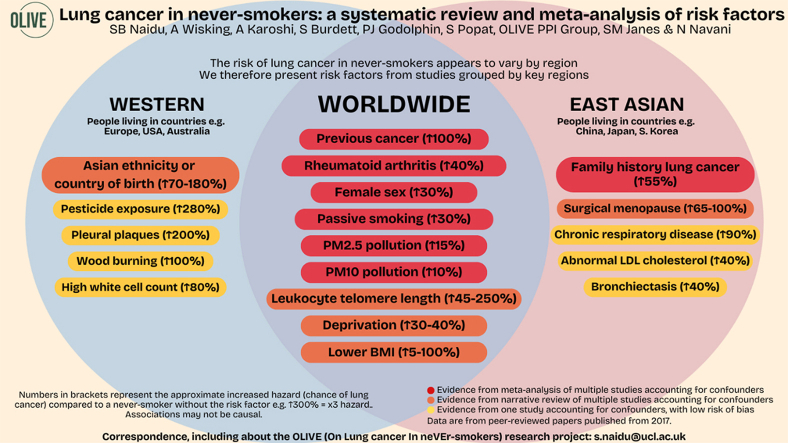

## Introduction

Lung cancer is the largest cause of cancer-related death worldwide, with 2,480,675 adults diagnosed and 1,817,469 people dying from it in 2022.^1^ Although often associated with smoking, reports from 2000 and 2020 respectively suggest up to 25% of lung cancer worldwide and 50% in East Asian countries occurs in “never-smokers” who have smoked less than 100 tobacco cigarettes in their lifetime.^2^^,^^3^ If considered separately, lung cancer in individuals who have never smoked (LCINS) is the seventh largest cause of cancer death worldwide.^2^

Approximately half of all individuals with lung cancer are diagnosed at stage IV, which is usually not curable and associated with increased mortality and poorer quality of life.^4^ A third of all individuals have multiple primary care presentations before being referred for further investigations, a higher proportion compared with most cancers.^5^ Never-smokers may experience longer delays to receiving a medical appointment and be more likely to have late-stage disease at diagnosis.^2^^,^^6^ There are now programs to improve early detection in individuals who have ever smoked, such as screening with low-dose computed tomography scans. Such programs have led to marked stage shifts, enabled the identification of individuals with early-stage disease, and contributed to marked improvements in mortality.^7^ Screening for LCINS has been introduced in some East Asian countries; most notably, in Taiwan, the prospective Taiwan Lung Cancer Screening in Never-Smoker Trial (TALENT) study reported on 12,011 participants, most (93.3%) of whom were never-smokers with the remaining being individuals who smoked less than 10 pack-years, aged 55 to 75 years with at least one risk factor of family history of lung cancer, passive smoking, tuberculosis, chronic pulmonary obstructive disease (COPD), high cooking index, or cooking without ventilation.^8^ The rate of screen-detected invasive lung cancer was 2.0% in the baseline round. Comparatively, randomized controlled trials such as Nederlands–Leuvens Longkanker Screenings Onderzoek (NELSON) and National Lung Screening Trial (NLST) screened individuals with significant smoking histories and reported rates of 0.9% to 1.0%.^9^^,^^10^ However, such trials also demonstrated a reduction in lung cancer-related mortality, paving the way for the widespread recommendation of screening for lung cancer in high-risk individuals who have ever smoked. As TALENT had no comparison arm, it was unable to demonstrate a reduction in lung cancer–related mortality. Furthermore, TALENT may have higher rates of overdiagnosis, especially as never-smokers may have more indolent lesions. The balance between benefits and risks and the efficacy of screening individuals who have never smoked remains widely debated. One way to maximize benefit may be to use risk-prediction models to identify high-risk individuals, rather than risk factor–based screening.^11^ However, there are currently no robust risk-prediction models to identify high-risk never-smokers who may benefit from such interventions in populations outside of East Asia.^3^ The lack of early detection programs for LCINS results in significantly fewer individuals identified with early-stage disease and lost opportunities for curing them.

Knowledge of risk factors would enable the identification of high-risk never-smokers who may benefit from such early detection interventions. Well-recognized risk factors include environmental exposures, such as passive smoking.^12^ There is a growing body of research investigating risk factors for LCINS, and summarizing this evidence is essential to ensure maximum benefit for patients. One systematic review, published in 2022, is available. However, it is not sufficiently comprehensive as it only included cohort studies.^13^ Furthermore, LCINS risk seems to vary by region. For example, most germline genetic variants associated with LCINS risk identified among East Asians are not found in Europeans.^14^ Moreover, somatic genomic architectures of LCINS in East Asians are different to Western populations, potentially reflecting varied etiologies.^2^

This study aimed to summarize the impact of risk factors on LCINS incidence worldwide and separately for Western and East Asian countries.

## Methods

This study was prospectively registered (CRD42022379253) on PROSPERO.^15^ Reporting standards are in accordance with the Preferred Reporting Items for Systematic Reviews and Meta-Analyses (PRISMA) guidelines.^16^

### Eligibility Criteria

Studies that reviewed the association of any risk factor with an outcome of primary LCINS incidence and which adjusted for confounding factors were included. The authors of this study chose to limit studies to those published from 2017. This was to ensure that this review reflects current evidence to maximize its relevance to never-smokers today, and that it would be feasible to complete given the large number of studies identified. Full criteria are listed in Table 1. Of note, potential confounding by individuals who have ever smoked was mitigated by ensuring appropriate identification of never-smokers, for example through study questionnaires.

**Table 1:** Inclusion and Exclusion Criteria

Criteria	Inclusion	Exclusion
Participant	• Adults aged ≥18 y old • Never-smokers	• Combine never-smokers with individuals who have previously smoked
Risk factor	• Any risk factor other than those meeting exclusion criteria	• Factors identified retrospectively from participants diagnosed with lung cancer • Factors derived from tumor tissue or somatic profile
Outcome	• Incidence of primary lung cancer	• Mortality from lung cancer • Risk of outcome estimated from modelling data instead of derived from study participants • Cancer from other organs which has metastasized to the lungs • Only reported on carcinoid tumors
Studies	• Published since 2017	• Not available in English • Not peer reviewed • Systematic reviews, meta-analyses, case reports, or series • Conference abstracts

### Search Strategy

With an experienced medical librarian, a search strategy was created and performed for MEDLINE via OVID, EMBASE, and Scopus (Appendix 1). Two reviewers (SBN and AW/AK) independently screened the abstracts and then the full text. Disagreements were resolved by discussion. Additional studies were identified by hand-searching reference lists of included articles and through prior knowledge of clinical experts.

### Data Extraction

Data were extracted using a predefined spreadsheet (Appendix 2) by one reviewer (AW/AK) and validated by a second (SBN). Study investigators were contacted at least twice for missing data.

### Risk of Bias

Two reviewers (SBN and AW/AK) independently appraised the studies using the Newcastle-Ottawa Scale (NOS).^17^ Discrepancies were resolved by discussion. NOS was chosen as it is appropriate for different study designs. Each study could receive a maximum total score of nine, with scores of zero to three, four to six, and seven to nine judged as high, moderate, and low risk of bias, respectively.

### Statistical Analysis

Following recommendations from the PROGRESS framework,^18^ effect estimates such as hazard ratios (HRs) that adjusted for confounding factors were extracted or calculated.^19^ Meta-analyses were performed if equivalent effect measures were available for at least two studies using a random-effects model with restricted maximum likelihood (REML) estimator. If more than five studies were included, Hartung-Knapp-Sidik-Jonkman correction was applied.^20^ Narrative synthesis was used where meta-analysis was not possible. Study cohorts were systematically reviewed for potential overlap before synthesis by comparing recruitment sites and time frames.

Heterogeneity was evaluated using Cochran’s Q test, with an a priori significance level of 10% and *I*^2^ to assess inconsistency between studies. The authors of this study aimed to perform study-level subgroup analyses by region (Western and East Asian), meta-regression by mean age of cohort, and sensitivity analyses excluding studies at high risk of bias.

Analyses were performed in STATA 17.^21^

### Patient and Public Involvement

The aim of patient and public involvement (PPI) was to review the protocol to ensure that it would meet diverse needs and provide insight on findings. Five patients with LCINS and two members of the public were consulted through 30-minute one-to-one meetings. These adults represented different ages, genders, and ethnicities and were compensated for their involvement. A checklist was consulted to report PPI.^22^

## Results

### Overview of Included Studies

**Figure 1:**
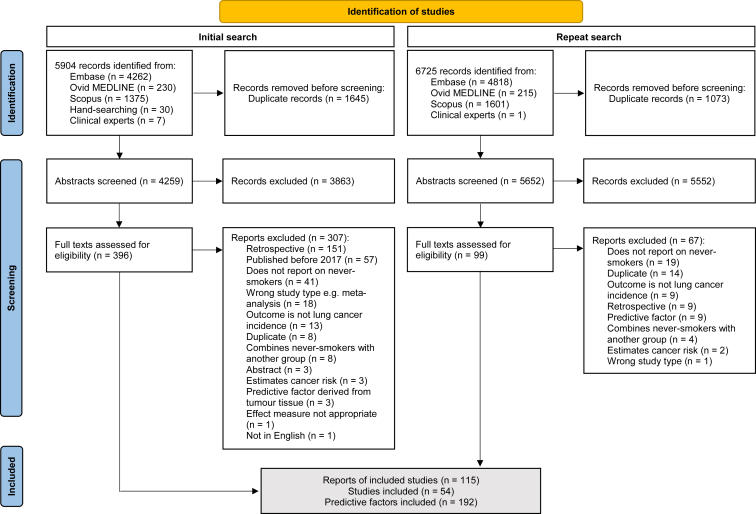
Preferred Reporting Items for Systematic Reviews and Meta-Analyses flow diagram. Adapted from: Page MJ, et al. The PRISMA 2020 statement: an updated guideline for reporting systematic reviews. BMJ 2021;372:n71. https://doi.org/10.1136/bmj.n71

Of 6,725 reports identified, 115 were included in this systematic review (Fig. 1). These reports were from 54 different studies reporting on 16,153,321 never-smokers; data for number of never-smokers were unavailable for two studies. Thirty-three studies with 8,241,269 never-smokers were in Western countries (including Australia, Europe, and USA), 20 studies with 7,870,686 never-smokers were in East Asian countries (including China, Japan, and South Korea), and one study in Iran. Study characteristics including gender and ethnicity breakdown where available are reported in Appendix 3.

**Figure 2:**
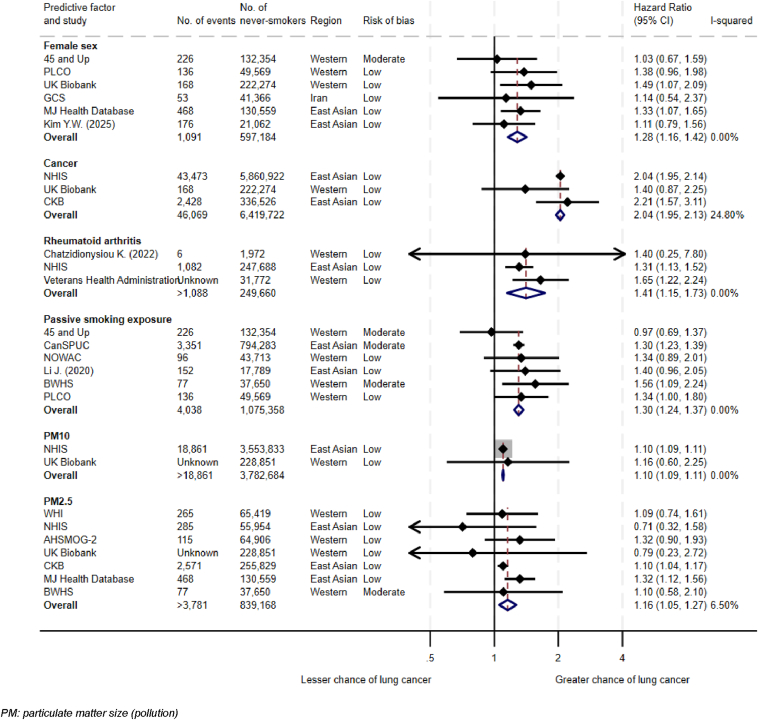
Meta-analyses of risk factors associated with LCINS. LCINS, lung cancer in individuals who have never smoked.

**Figure 3:**
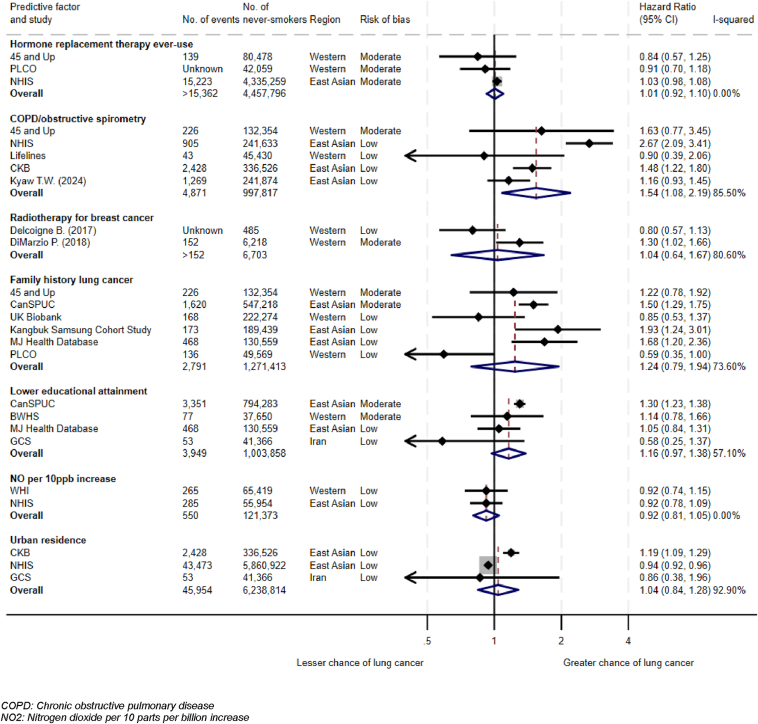
Meta-analyses of risk factors not associated with LCINS. LCINS, lung cancer in individuals who have never smoked.

This study reports on 192 risk factors investigated through multivariable analysis. The confounding factors adjusted for in these analyses are reported in Appendix 3. A summary of meta-analyses is available in Figures 2 and 3. Factors found to be associated with LCINS are summarized in Table 2.^23^, ^24^, ^25^, ^26^, ^27^, ^28^, ^29^, ^30^, ^31^, ^32^, ^33^, ^34^, ^35^, ^36^, ^37^, ^38^, ^39^, ^40^, ^41^, ^42^, ^43^, ^44^, ^45^, ^46^, ^47^, ^48^, ^49^, ^50^, ^51^, ^52^, ^53^, ^54^, ^55^, ^56^, ^57^, ^58^, ^59^, ^60^, ^61^, ^62^, ^63^, ^64^, ^65^, ^66^, ^67^, ^68^, ^69^, ^70^, ^71^, ^72^, ^73^, ^74^, ^75^, ^76^ A full list of meta-analyses performed and risk factors extracted is available in Appendices 4 to 5.

**Table 2:** Factors Associated With LCINS in Meta-Analyses and Single Studies

Factor	Studies	Hazard Ratio (95% CI)	*p* Value	No. of Never-Smokers	Risk of Bias
Factors associated with women					
Female sex (6 of 7 studies)	45 and Up^23^	**1.28 (1.12–1.47)**	0.005	597,184	6
	PLCO^24^				7
	UK Biobank^25^				9
	MJ Health Database^26^				7
	GCS^27^				9
	Kim et al.,^28^ 2025				8
Breastfeeding	NHIS^29^	**0.94 (0.90–0.98)**	0.008	4,907,697	6
	CanSPUC^30^				5
	JPHC^31^				6
Menopausal status	CanSPUC^30^	Natural menopause: Ref **Surgical:1.67 (1.32–2.11)**	0.0002	529,823	5
	JPHC^31^	Premenopausal: Ref Natural: 1.46 (0.85**–**2.50) **Surgical:1.99 (1.10–3.59)**	0.17 0.02	42,615	6
Benign breast disease	CanSPUC^30^	**1.24 (1.10–1.41)**	0.0007	529,823	5
Respiratory comorbidities					
Pleural plaques	ARDCO^32^	**3.13 (1.04–9.35)**	0.04	1,305	9
Bronchiectasis	NHIS^33^	**1.44 (1.31–1.57)**	<0.001	2,323,605	9
Pulmonary tuberculosis	NHIS^34^	**1.42 (1.04–1.95)** Without COPD: 0.91 (0.62**–**1.33)	0.03 0.64	32,086	7
Obstructive sleep apnea	NHS^35^	**2.96 (1.42–6.18)**	0.004	31,323	6
Chronic respiratory disease	CanSPUC^36^	**1.94 (1.24**-**3.04)**	0.004	75,917	7
Other comorbidities					
Rheumatoid arthritis	Chatzidionysiou et al.,^37^ 2022	**1.41 (1.15–1.73)**	0.001	281,432	8
	NHIS^38^				8
	Veterans Health Administration^39^				7
Cancer	UK Biobank^25^	**2.04 (1.95–2.13)**	<0.0001	6,419,722	9
	CKB^40^				8
	NHIS^41^				9
Hypertension	NHIS^29^	**1.16 (1.01–1.34)**	0.04	5,260,101	6
	MJ Health Database^26^				7
	CanSPUC^42^				5
Symptomatic peripheral arterial disease	ARIC^43^	**9.08 (1.16–71.34)**	0.04	5,548	8
Hepatobiliary disease	CanSPUC^42^	**1.29 (1.21–1.37)**	<0.0001	794,283	5
Gastrointestinal diseases	CanSPUC^42^	**1.30 (1.22–1.38)**	<0.0001	794,283	5
Oral microbiome	SMHS and SWHS^44^	Some associated with increased risk, e.g., *Lactobacillales* order medium vs. low OR **2.15 (1.03–4.47)**, some with decreased, e.g., *Bacteroidetes* order high vs. low OR **0.31 (0.15–0.64)**	Various	180	8
Medication					
Anti-hypertensives (1 of 2 studies)	NHIS^45^	CCB: Ref **ARB: 0.64 (0.42–0.99)**	0.04	39,784	9
Oral bisphosphonate	WHI^46^	**0.57 (0.39–0.84)**	0.004	75,962	7
Metformin use for diabetes	NHIS^47^	**Compared with non-diabetics:** **0.86 (0.74–0.99)** Compared with diabetics who had never used metformin: 0.87 (0.71**–**1.07)	0.04 0.18	515,100	9
Aspirin, statin, and metformin	NHIS^47^	Use ≥547.5 d: **0.38 (0.20–0.73)**	0.003	515,100	9
Family history					
Number of relatives with lung cancer	CanSPUC^42^	**1.31 (1.24–1.39)**	<0.0001	794,283	5
Father	CanSPUC^48^	**2.15 (1.66–2.77)**	<0.0001	547,218	6
Mother	CanSPUC^48^	**1.33 (1.14–1.57)**	0.0005	547,218	6
Siblings	CanSPUC^48^	**2.02 (1.30–3.12)**	0.002	547,218	6
Family history of any cancer	CanSPUC^42^	**1.31 (1.24–1.40)**	<0.0001	794,283	5
Sociodemographic factors					
Deprivation (2 of 3 studies)	BWHS^49^	**1.30 (1.03–1.63)** per 10 U increase of neighborhood concentrated disadvantage index	0.02	37,650	5
	THIN^50^	Women^a^: 1 (least deprived): Ref 2: 1.01 (0.88–1.16) 3: 0.92 (0.79–1.07) 4: 0.98 (0.83–1.15) 5: 1.13 (0.94–1.36) Men^a^: 1 (least deprived): Ref 2: 0.98 (0.84–1.15) 3: 1.01 (0.85–1.20) **4: 1.24 (1.04–1.48)** **5: 1.42 (1.15–1.76)**	0.31 0.002	3,679,831	7
Ethnicity	DeRouen et al.,^51^ 2022	Non-Hispanic white: Ref AANHPI: **1.69 (1.38–2.02)** Black: Not available Hispanic: 0.84 (0.56**–**1.23)^a^	**<0.0001** 0.39	889,870	6
	PLCO^51^	Non-Hispanic white: Ref Hispanic: 0.76 (0.11**–**5.58) Asian: 1.00 (0.41**–**2.50)	0.79 0.995	49,569	7
Country of birth	45 and Up^23^	**Asian: 2.83 (1.64–4.89)**	**0.0002**	132,354	6
Physical measurements					
BMI (5 of 9 studies)	SIDIAP^77^	**0.95 (0.91–1.00)** per 5 kg/m^2^ increase	0.03	2,339,363	8
	45 and Up^23^	**<18.5: 1.98 (1.16–3.39)** 18.5**–**25: Ref ≥ 25**–**< 30: 0.92 (0.65**–**1.30) ≥ 30: 1.01 (0.64**–**1.59)	0.05	132,354	6
	NHIS^29^	<18.5: 0.93 (0.83**–**1.03) 18.5**–**<23: Ref **23–<25: 0.95 (0.91–0.99)** **25–<30: 0.95 (0.91–0.99)** ≥30: 0.97 (0.90**–**1.05)	0.19 0.02 0.02 0.45	4,335,259	6
	CKB^40^	**<18.5: 1.45 (1.21–1.73)** **18.5–23.9: 1.17 (1.08–1.27)** ≥24: Ref	**<0.001** **<0.001**	336,526	8
	MJ Health Database^26^	<18.5: 0.77 (0.50**–**1.18) 18.5**–**23.9: Ref **24–27.9: 0.80 (0.64–0.99)** ≥28: 0.72 (0.51**–**1.03)	0.23 **0.04** 0.08	130,559	7
BMI change	Kailuan^52^	Major loss <1.0 kg/m^2^/y: **1.97 (1.12–3.45)** Major gain ≥1.0 kg/m^2^/y: **2.15 (1.15–4.02)**	**0.02** **0.02**	37,085	7
Lifestyle factors					
Sleep duration (<6 or >9 h)	CanPath^53^	**1.52 (1.01–2.29)**^b^	**0.0008**	950	7
Environmental factors					
Passive smoking	45 and Up^23^	**1.30 (1.22–1.40)**	<0.0001	1,075,358	6
	CanSPUC^42^				5
	BWHS^49^				5
	PLCO^24^				7
	NOWAC^54^				7
	Li et al.,^55^ 2020				7
PM_10_	NHIS^56^	**1.10 (1.09–1.11)**	<0.001	3,782,684	8
	UK Biobank^57^				9
PM_2.5_	WHI^58^	**1.16 (1.03–1.30)**	0.02	839,168	8
	NHIS^59^				9
	AHSMOG-2^60^				7
	UK Biobank^57^				9
	CKB^61^				9
	MJ Health Database^26^				7
	BWHS^49^				5
Wood-burning	Sister Study^78^	1**–**29/d/y: 1.64 (0.87**–**3.10) **≥1 mo/y:** **1.99 (1.02–3.89)**	0.13 0.04	28,290	7
Pesticides, e.g., chlorimuron-ethyl	AHS^62^	Non-exposed: Ref <median: 2.17 (0.92**–**5.11) **>median: 3.80 (1.53–9.48)**	0.08 0.004	26,859	7
Distance to A1 (busy London road) (m)	WHI^58^	>200: Ref 50**–**<200: 0.82 (0.34**–**1.98) **<50: 5.23 (1.94–14.13)**	0.67 0.001	65,419	8
Occupational exposure to hazardous substances	CanSPUC^42^	**1.29 (1.21–1.37)**	**<0.0001**	794,283	5
Air pollution	CanSPUC^42^	**1.29 (1.22–1.37)**	<0.0001	794,283	5
Cooking oil fumes	CanSPUC^42^	**1.29 (1.22–1.37)**	<0.0001	794,283	5
Dietary intake					
AHEI-2010 (2 of 3 studies)	MEC^63^	25.1**–**56.6: Ref 56.7**–**62.2: 0.83 (0.64**–**1.08) 62.3**–**67.1: 0.82 (0.63**–**1.06) 67.2**–**72.6: 0.91 (0.71**–**1.18) **72.7–104.5: 0.66 (0.50–0.87)**	0.16 0.14 0.48 **0.003**	80,635	9
	WHI^64^	Q1: Ref Q2: 0.69 (0.47**–**1.02) **Q3: 0.60 (0.40–0.91)** **Q4: 0.59 (0.39–0.93)** Q5: 0.98 (0.67**–**1.43)	0.06 **0.02** **0.02** 0.92	41,950	7
Laboratory tests					
White blood cell count	UK Biobank^65^	Women ≤5.55: Ref 5.55**–**6.51: 1.27 (0.76**–**2.13) 6.51**–**7.67: 1.09 (0.64**–**1.88) **>7.67: 1.82 (1.10–3.00)**	0.37 0.75 0.02	232,528	9
Neutrophil-to-lymphocyte ratio	UK Biobank^65^	Women: **1.20 (1.01–1.43)** Men: 1.06 (0.85**–**1.33)	0.03 0.62	232,528	9
Platelet count	UK Biobank^66^	Women: **1.18 (1.04–1.34)** Men: 1.13 (0.95**–**1.34)	0.01 0.18	222,893	8
eGFR	MJ Health Database^26^	≥90: Ref **60–89: 1.58 (1.25–2.00)** **45–59: 1.85 (1.23–2.79)** <45: 1.47 (0.54**–**4.06)	<0.001 0.003 0.45	130,559	7
Total cholesterol	Kailuan^67^	**Q1: 1.42 (1.05–1.93)** Q2: Ref **Q3: 1.46 (1.07–2.00)** Q4: 1.24 (0.90**–**1.70) **Q5: 1.38 (1.01–1.89)**	0.02 0.02 0.19 0.04	56,097	7
LDL-C	Kailuan^67^	**Q1: 1.39 (1.02–1.89)** Q2: Ref Q3: 1.17 (0.83**–**1.65) **Q4: 1.58 (1.14–2.20)** **Q5: 1.42 (1.00–2.02)**	0.04 0.38 0.006 0.05	56,097	7
Folate	CHHRS^68^	<6.72 ng/mL: **1.72 (1.11–2.66)**^b^	0.01	555	8
5-mTHF	CHHRS^68^	<5.80 ng/mL: **1.58 (1.02–2.43)**^b^	0.04	555	8
CEA	MJ Health Database^26^	**1.06 (1.04–1.08)**	<0.001	130,559	7
sIL-6R	SWHS^69^	Q1: Ref Q2: 1.20 (0.69**–**2.10) **Q3: 1.91 (1.12–3.23)** **Q4: 2.37 (1.40–4.02)**^b^	0.54 0.02 0.001	511	9
IL-21	SWHS^69^	Q1: Ref Q2: 0.99 (0.59**–**1.64) **Q3: 0.51 (0.29–0.89)** **Q4: 0.53 (0.31–0.93)**^b^	0.97 0.02 0.02	511	9
Genetic information					
Leukocyte telomere length	Singapore Chinese Health Study^70^	Adenocarcinoma Q1 (shortest): Ref Q2: 1.22 (0.65**–**2.29) Q3: 1.53 (0.84**–**2.81) Q4: 1.39 (0.74**–**2.60) **Q5: 3.14 (1.80–5.49)**	0.55 0.17 0.31 <0.001	18,034	8
	SMHS^71^	Q1 (shortest): Ref Q2: 1.37 (0.71**–**2.62) **Q3: 3.48 (1.85–6.57)** Q4: 1.85 (0.99**–**3.44)^b^	0.35 0.0001 0.05	323	8
	SWHS^71^	Q1 (shortest): Ref **Q2: 1.45 (1.05–2.02)** **Q3: 1.76 (1.28–2.43)** **Q4: 2.10 (1.52–2.90)**	0.03 0.0005 <0.0001	1590	8
	UK Biobank^72^	Per IQR change: **1.45 (1.23–1.71)**	<0.001	234,302	8
Lung cancer**–**related CpG sites	NOWAC^73^	Various, including cg10151248-PC **0.36 (0.17-0.77)**^b^ and cg13482620-B3GNTL1 **0.31 (0.14-0.67)**^b^	Various	71	7
Urinary tests					
Urine metabolomics	SWHS^74^	pos_2.61_127.0382m/z **0.57 (0.46–0.72)**^b^ neg_2.60_369.0408m/z **0.97 (0.96–0.98)**^b^ pos_2.61_184.0325n **0.55 (0.43–0.71)**^b^ 5-methyl2-furoic acid Tertile 1: Ref **T2: 0.52 (0.34–0.80)** **T3: 0.46 (0.30–0.70)**^b^	<0.001 <0.001 <0.001 0.003 <0.001	564	9
Urinary phytoestrogens	SWHS^75^	Isoflavones Q1: Ref **Q2: 0.57 (0.39–0.83)** **Q3: 0.64 (0.44–0.92)** **Q4: 0.60 (0.41–0.86)**^b^	**0.04**	956	8
Urinary benzothiazole, benzotriazole, and derivatives	QEEHH^76^	Urinary 2-hydroxy-benzothiazole **2.44 (1.29–4.62)**^b^	**<0.01**	228	8

*Note:* Values in bold are statistically significant.

5-mTHF, etabolically active folate; BMI, body mass index; CCB, calcium channel blocker; CEA, carcinoembryonic antigen test; CI, confidence interval; COPD, chronic obstructive pulmonary disease; eGFR, estimated glomerular filtration rate; IL, interleukin; IQR, interquartile range; LCINS, lung cancer in individuals who have never smoked; LDL-C, low density lipoprotein-cholesterol; PM, particulate matter; Ref, reference; sIL-6R, soluble interleukin-6 receptor.

a Incidence rate ratio.

b OR.

### Factors Associated With Women

Female sex was associated with LCINS in meta-analysis of six studies (adjusted HR [aHR] 1.28 [95% CI 1.12–1.47], *p* = 0.005, *I*^2^ *=* 0.0%, Hetp = 0.73, n = 597,184; Fig. 2).^23^, ^24^, ^25^, ^26^, ^27^, ^28^

Breastfeeding (aHR 0.94 [95% CI 0.90–0.98], *p* = 0.008, *I*^2^ *=* 0.0%, Hetp = 0.66, n = 4,907,697)^29^, ^30^, ^31^ was minimally protective against LCINS in meta-analysis of three studies.

Individuals with surgical menopause were more likely to have LCINS than those who had undergone natural menopause (aHR 1.67 [95% CI 1.32–2.11], n = 529,823)^30^ and those who were premenopausal (aHR 1.99 [95% CI 1.10–3.59], n = 42,615) when adjusted for age and other confounders.^31^

None of the other factors associated with women reported in multiple studies, including hormone replacement therapy (Fig. 3, Appendix 5), were consistently associated with LCINS.

Of four factors reported in one study each, only benign breast disease (aHR 1.24 [95% CI 1.10–1.41], n = 529,823) was associated with LCINS.^30^

All studies had low-moderate risk of bias.

### Respiratory Comorbidities

There was no statistical evidence that COPD/obstructive spirometry was associated with LCINS in meta-analysis of five studies with low-moderate risk of bias (aHR 1.54 [95% CI 0.94–2.54], *p* = 0.07, *I*^2^ = 85.5%, Hetp < 0.0001, n = 997,817; Fig. 3).^23^^,^^40^^,^^79^, ^80^, ^81^ None of these studies accounted for pollution exposure as a confounder; all confirmed never-smoking status through questionnaires.

Of factors reported in single studies with low risk of bias, pleural plaques in a study adjusting for asbestos exposure (aHR 3.13 [95% CI 1.04–9.35], n = 1305),^32^ bronchiectasis (aHR 1.44 [95% CI 1.31–1.57], n = 2,323,605),^33^ and chronic respiratory disease (aHR 1.94 [95% CI 1.24–3.04], n = 75,917)^36^ were associated with LCINS. In a study with moderate risk of bias, obstructive sleep apnea (aHR 2.96 [95% CI 1.42–6.18], n = 31,323)^35^ was associated with LCINS; this was adjusted for body mass index (BMI). Although pulmonary tuberculosis was associated with LCINS (aHR 1.42 [95% CI 1.04–1.95]) in a study with low risk of bias, this association did not persist among participants without COPD (aHR 0.91 [95% CI 0.62–1.33], n = 32,086).^34^

### Other Comorbidities

Meta-analysis was performed for nine factors (Appendix 4). Previous history of cancer (aHR 2.04 [95% CI 1.95–2.13], *p* < 0.0001, *I*^2^ = 24.8%, Hetp = 0.27, n = 6,419,722; Fig. 2)^25^^,^^40^^,^^41^ was associated with LCINS in three studies, all with very low risk of bias.

Rheumatoid arthritis (aHR 1.41 [95% CI 1.15–1.73], *p* = 0.001, *I*^2^ = 0.0%, Hetp = 0.41, n = 281,432; Fig. 2)^37^, ^38^, ^39^ was associated with LCINS in three studies, all with low risk of bias. One of these studies also reported that mortality from lung cancer in participants with rheumatoid arthritis was similar to incidence, reducing the likelihood that this association is due to lead-time bias and increased imaging.

Hypertension (aHR 1.16 [95% CI 1.01–1.34], *p* = 0.04, *I*^2^ = 93.5%, Hetp < 0.0001, n = 5,260,101; Appendix 4)^26^^,^^29^^,^^42^ was associated with LCINS in three studies with low-moderate risk of bias.

Notably, meta-analysis of two studies with low-moderate risk of bias did not find receiving radiotherapy for primary breast cancer (aHR 1.04 [95% CI 0.64–1.67], *p* = 0.56, *I*^2^ = 80.6%, Hetp = 0.02, n = 6703; Fig. 3)^82^^,^^83^ was associated with LCINS.

Symptomatic peripheral arterial disease (aHR 9.08 [95% CI 1.16–71.34], n = 5548)^43^ was associated with LCINS in a single study with low risk of bias. This association persisted even after adjustment for possible confounders, including cardiovascular disease and diabetes. Certain oral microbiome was also associated with LCINS in a study with low risk of bias (Table 2).

In studies with moderate risk of bias, hepatobiliary (aHR 1.29 [95% CI 1.21–1.37])^42^ and gastrointestinal diseases (aHR 1.30 [95% CI 1.22–1.38], both n = 794,283)^42^ were associated with LCINS.

### Family History

Meta-analysis of six studies globally with low-moderate risk of bias did not find family history of lung cancer to be associated with LCINS (aHR 1.24 [95% CI 0.79–1.95], *p* = 0.03, *I*^2^ = 73.6%, Hetp = 0.002, n = 1,271,413; Fig. 3).^23^, ^24^, ^25^, ^26^^,^^48^^,^^84^ Three of these studies adjusted for passive smoking and another adjusted for pollution.

Of note, subgroup analysis found that family history of lung cancer differed by region (*p* value for subgroup differences = 0.006); it was associated with LCINS in three studies with low-moderate risk of bias in East Asia (aHR 1.56 [95% CI 1.23–1.98], *I*^2^ = 0.0%, n = 867,216) but not in three studies with low-moderate risk of bias in Western countries (aHR 0.86 [95% CI 0.35–2.11], *I*^2^ = 52.8%, n = 404,197).

One East Asian study, which had moderate risk of bias, did report an association between LCINS and maternal, sibling, and especially paternal lung cancer (paternal lung cancer aHR 2.15 [95% CI 1.66–2.77], n = 547,218), even after adjusting for passive smoking.^48^ It also found an increasing number of relatives with lung cancer increased risk of LCINS (aHR 1.31 [95% CI 1.24–1.39], n = 794,283).^42^

An East Asian study with moderate risk of bias also found that family history of any cancer (aHR 1.31 [95% CI 1.24–1.40], n = 794,283) was associated with LCINS.^42^

### Sociodemographic Factors

There was no association between educational attainment and LCINS in meta-analysis of four studies with low-moderate risk of bias (aHR 1.16 [95% CI 0.97–1.38], *p* = 0.10, *I*^2^ = 57.10%, Hetp = 0.07, n = 1,003,858; Fig. 3).^26^^,^^27^^,^^42^^,^^49^

Two of three studies reporting on deprivation found an association with LCINS (Appendix 5); men living in more deprived areas (highest deprivation score: incidence rate ratio [IRR] 1.42 [95% CI 1.15–1.76], n = 3,679,831)^50^ were more likely to have LCINS in one study with low risk of bias, whereas living in a more deprived neighborhood (aHR 1.30 [95% CI 1.03–1.63], n = 37,650)^49^ was associated with LCINS in another study with moderate risk of bias.

There was no association between LCINS and income in two studies with moderate risk of bias (Appendix 5).^29^^,^^49^

Two studies performed a multivariable analysis of LCINS and ethnicity. Both studies were of individuals living in America. One study with low risk of bias found that Hispanic and Asian ethnicities were not associated with LCINS (e.g., Asian ethnicity aHR 1.00 [95% CI 0.41–2.50], n = 49,569).^24^ Another larger study but with moderate risk of bias reported an increased incidence of LCINS among women of Asian American, Native Hawaiian, and Pacific Islander ethnicity (IRR 1.69 [95% CI 1.38–2.02], n = 889,870) compared with non-Hispanic white women.^51^ In addition, an Australian study with moderate risk of bias found participants who had been born in Asian countries had increased risk of LCINS (aHR 2.83 [95% CI 1.64–4.89], n = 132,354) compared with those born in non-Asian countries.^23^

### Physical Measurements

Of nine studies with low-moderate risk of bias, five found lower BMI associated with LCINS (n = at least 7,366,661 with one study having unknown number of never-smokers; Table 2). For example, two studies reported BMI less than 18.5 was associated with LCINS (aHR 1.98 [95% CI 1.16–3.39] compared with BMI 18.5–25, n = 132,354^23^ and aHR 1.45 [95% CI 1.21–1.73] compared with BMI more than or equal to 24, n = 336,526).^40^ Furthermore, BMI change of more than or equal to 1.0 kg/m^2^/y was associated with LCINS (loss: aHR 1.97 [95% CI 1.12–3.45], gain: aHR 2.15 [95% CI 1.15–4.02], n = 37,085) in a study with low risk of bias.^52^

Three of five studies (n = at least 542,737) with low-moderate risk of bias did not find an association between LCINS and height (Appendix 5).

### Lifestyle Factors

Only two of eight studies (n = at least 4,501,339) with low-moderate risk of bias reported an association between LCINS and physical inactivity, and only one of six studies (n = at least 5,458,489) with low-moderate risk of bias reported an association with alcohol consumption (Appendix 5).

One study with low risk of bias found sleep duration of less than six or more than nine hours was associated with LCINS (aOR 1.52 [95% CI 1.01–2.29], n = 950).

### Environmental Factors

Meta-analysis of six studies with low-moderate risk of bias found an association between passive smoking and LCINS (aHR 1.30 [95% CI 1.22–1.40], *p* < 0.0001, *I*^2^ = 0.0%, Hetp = 0.55, n = 1,075,358; Fig. 2).^23^^,^^24^^,^^42^^,^^49^^,^^54^^,^^55^ Two studies, both with low risk of bias, further reported on in utero^85^ and childhood smoking exposure^24^; neither was associated with LCINS.

Meta-analysis of two studies with low risk of bias found pollution as measured by PM10 was associated with LCINS (aHR 1.10 [95% CI 1.09–1.11], *p* < 0.001, *I*^2^ = 0.0%, Hetp = 0.88, n = 3,782,684; Fig. 2).^56^^,^^57^^,^^59^ Meta-analysis of seven studies, of which six had low and one had moderate risk of bias, found pollution as measured by PM2.5 was also associated with LCINS (aHR 1.16 [95% CI 1.03–1.30], *p* = 0.02, *I*^2^ = 6.5%, Hetp = 0.38, n = 839,168; Fig. 2).^26^^,^^49^^,^^57^, ^58^, ^59^, ^60^, ^61^ Only one of these studies adjusted for other pollution measures.

Neither meta-analysis of two studies reporting nitrogen dioxide (NO_2_) per 10 ppb (aHR 0.92 [95% CI 0.81–1.05], *p* = 0.22, *I*^2^ = 0.00%, Hetp = 1.00, n = 121,373; Fig. 3)^58^^,^^59^ nor a single study reporting NO_2_ per 10 μg/m^3^ (aHR 0.98 [95% CI 0.85–1.13], n =  246,664; Appendix 5)^57^ found an association with LCINS. All three studies had low risk of bias.

Neither meta-analysis of three studies reporting on hazard of urban residence (aHR 1.04 [95% CI 0.84–1.28], *p* = 0.71, *I*^2^ = 92.90%, Hetp < 0.0001, n = 6,238,814; Fig. 3)^27^^,^^40^^,^^41^ nor a single study reporting incidence rate ratios (Appendix 5)^50^ found an association with LCINS. All four studies had low risk of bias.

Of factors reported in single studies with low risk of bias, exposures to wood-burning more than one month a year (aHR 1.99 [95% CI 1.02–3.89], n = 28,290),^78^ a busy London road (aHR 5.23 [95% CI 1.94–14.13], n = 65,419),^58^ and pesticides such as chlorimuron-ethyl (aHR 3.80 [95% CI 1.53–9.48], n = 26,859)^62^ were associated with LCINS. In a study with moderate risk of bias (n = 794,283), occupational exposures to hazardous substances (aHR 1.29 [95% CI 1.21–1.37]), air pollution, and cooking oil fumes (both aHR 1.29 [95% CI 1.22–1.37]) were also associated with LCINS.^42^ Of note, occupational radon exposure was not associated with LCINS (Appendix 5).^86^

### Laboratory Tests

#### Blood Tests

The association between LCINS and testosterone, C-reactive protein, and bilirubin was reviewed in two studies each with low-moderate risk of bias; there was no consistent association of these blood tests with LCINS (Appendix 5).

In single studies with low risk of bias, women with high white blood cell count (aHR 1.82 [95% CI 1.10–3.00]),^65^ neutrophil-to-lymphocyte ratio (aHR 1.20 [95% CI 1.01–1.43], both n = 232,528),^65^ and platelet count (aHR 1.18 [95% CI 1.04–1.34], n = 222,893)^66^ and adults with deranged kidney function (eGFR 45–59 aHR 1.85 [95% CI 1.23–2.79], n = 130,559),^26^ total cholesterol, and low-density lipoprotein-cholesterol (Table 2; n = 56,097) were more likely to have LCINS.^67^ Low folate (aOR 1.72 [95% CI 1.11–2.66]) and 5-mTHF (metabolically active folate, aOR 1.58 [95% CI 1.02–2.43], both n = 555) were associated with LCINS.^68^ Carcinoembryonic antigen (CEA) was also associated with LCINS (aHR 1.06 [95% CI 1.04–1.08], n = 130,559).^26^

In a study with low risk of bias, certain immune markers including high s-IL6R (a regulator of inflammatory response, aOR 2.37 [95% CI 1.40–4.02]) and low IL-21 (involved in immune cell proliferation and differentiation, aOR 0.53 [95% CI 0.31–0.93], all n = 511) (Table 2) were associated with LCINS at mean follow-up of 7.5 years.^69^

Of note, vitamin D level was not associated with LCINS in a study with low risk of bias.^87^

#### Genetic Information

Meta-analysis of two studies with low risk of bias found no association between LCINS and mosaic loss of chromosome Y (aHR 0.99 [95% CI 0.75–1.32), *p* = 0.97, *I*^2^ = 17.4%, Hetp = 0.27, n = 117,392)^88^ or clonal hematopoeisis (aHR 1.55 [95% CI 1.00–2.41], *p* = 0.05, *I*^2^ = 0.0%, Hetp = 0.85, n = 1273).^89^

Leukocyte telomere length was reported in four studies with low risk of bias (n = 254,249, Table 2) and all were suggestive of an association between longer length and LCINS (e.g., per interquartile range (IQR) change aHR 1.45 [95% CI 1.23–1.71], n = 234,302).^72^

In a single study with low risk of bias, various lung cancer–related CpG sites were found to be associated with LCINS. For example, cg10151248-*PC* and cg13482620-*B3GNTL1* were found to be associated with reduced future risk of LCINS (aOR 0.36 [95% CI 0.17–0.77] and aOR 0.31 [95% CI 0.14–0.67], respectively; n = 71).^73^

Factors associated with medication, dietary intake, and urine laboratory tests are available in Appendix 6.

### Subgroup Analyses by Geographic Region

Prespecified subgroup analysis by geographic region was able to be performed for 11 of 32 meta-analyses (Appendix 7). Only two factors differed by region: family history of lung cancer (discussed previously, *p* value for subgroup differences = 0.006) and periodontitis (*p* value for subgroup differences = 0.001).

Periodontitis was associated with LCINS in one study with low risk of bias in East Asia (aHR 3.56 [95% CI 2.68–4.72], n = 90,947) but not in two studies with low-moderate risk of bias in Western countries (aHR 1.11 [95% CI 0.61–2.03], *I*^2^ = 47.6%, n = 36,944). None of these studies adjusted for socioeconomic status as a possible confounder.

### Subgroup Analyses by Age

Subgroup analysis by age was able to be performed for 12 of 32 meta-analyses (Appendix 7); there was no evidence that mean age affected association of any risk factor with LCINS.

### Risk of Bias

No studies were assessed as having high risk of bias (Appendix 8). Of note, 16 of 32 meta-analyses included studies with low risk of bias. These include meta-analyses of studies reviewing history of cancer, rheumatoid arthritis, cardiovascular disease, PM10, and NO_2_.

**Figure 4:**
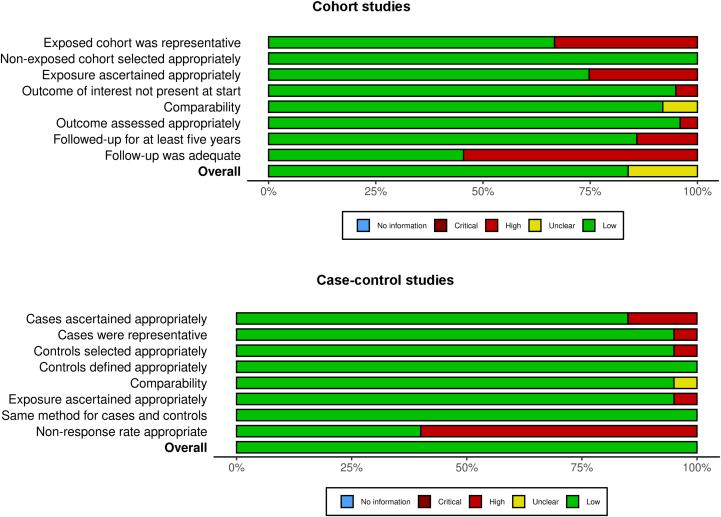
Summary of risk of bias for cohort and case-control studies.

The overall risk of bias across each domain is summarized in Figure 4.^90^ Cohort studies scored lowest for adequacy of follow-up, with 45.5% of reports accounting for at least 90% of subjects or describing participants lost to follow-up. Only 67.3% used a cohort which was representative of an average never-smoker in the community. Case-control studies scored lowest for response rate, with 40.0% specifying the same rate for both groups.

## Discussion

This study comprehensively reviewed and summarized 192 risk factors with 32 meta-analyses for LCINS. Meta-analysis of studies using multivariable analysis to account for possible confounding factors quantifies the association of factors associated with LCINS diagnosis, including female sex, previous history of cancer, rheumatoid arthritis, passive smoking, and pollution as measured by PM10 and PM2.5. Of note, in planned subgroup analyses, family history of lung cancer was associated with LCINS in East Asian but not Western countries. Narrative review also suggests that surgical menopause, deprivation, Asian ethnicity and country of birth, lower BMI, increased height and leukocyte telomere length are associated with LCINS. No studies were assessed as having high risk of bias.

Lung cancer referral guidelines and risk assessment tools usually prioritize smoking status and may not relate specifically to never-smokers, making such tools less valuable for identifying high-risk never-smokers.^91^ These factors are therefore useful for clinicians to review during consultations to guide appropriate referral and investigations.

This systematic review also provides a basis for further research and designing early diagnosis interventions. Together with their patient representatives, the authors of this study advocate prioritizing four areas. First, the review provides evidence for and quantifies the association of factors that may be combined to develop risk prediction models for screening.

Second, their patients also advocate investigating the causality of these factors. Of note, female sex was associated with LCINS. However, among factors associated with women, only surgical menopause was significantly associated with LCINS, whereas other factors including hormone replacement therapy, hormonal contraception, and parity were not associated. Further research to determine causality could lead to more effective risk mitigation strategies in this important group.

Third, although this study aimed to review differences between Western and East Asian countries, few studies were found outside these areas and further studies in other geographical areas should be supported. In Ghana, for example, 61% of lung cancer is identified in never-smokers,^92^ but risk factors are poorly understood.

Finally, heterogeneity in reporting risk factors complicates public health messaging and undermines early detection interventions.^93^ A global registry of lung cancer risk studies with standardized methods and a minimum data set including data such as sex and smoking status would significantly improve understanding of risk factors and research applicability. This study also identifies multiple factors that were only reported by single studies, and further evidence is required to validate the findings.

This review is unique for its inclusion of PPI who developed and supported the study and co-designed an infographic for dissemination of key findings. Researchers felt that there were important positive consequences from PPI input. Patient feedback helped researchers to understand what was important and led to protocol amendments; for example, subgroup analyses by geographical region and age were prespecified. Patients reviewed findings to provide their perspective and would like to highlight its importance to different groups. For example, factors such as passive smoking and deprivation, if addressed by individuals or organizations, can affect LCINS incidence. Factors such as rheumatoid arthritis are important for clinicians to be aware of when reviewing patients to ensure prompt investigation and referral of symptoms.

This review has other strengths. The authors of this study were comprehensive in their summary by extracting relevant information on never-smokers only from studies with mixed populations of individuals who have never and ever smoked. The applicability of the findings is increased as recommended review methodology was used.^18^ Furthermore, no studies had high risk of bias.

Nevertheless, this review has some limitations. Many risk factors were reviewed by a small number of studies with high heterogeneity, limiting further analysis including subgroup and reporting bias. Risk factors reported as not associated with LCINS may therefore reflect a lack of statistical significance. Furthermore, these limited data preclude reviewing subgroup differences by ethnicity and between people of East Asian ethnicity living in Western and East Asian countries. As LCINS rates likely differ between ethnic groups, the impact of risk factors on people from different ethnicities requires further study.^94^ Data on risk factors associated with lung cancers with specific mutations such as EGFR were also unavailable in this review. Furthermore, some studies did not account for any confounding factors and were not included in this review. Others did not adjust for potentially important confounding factors. In particular, pollution exposure was rarely measured or adjusted for and cannot be excluded as a confounder, for example, in the relationship between family history of lung cancer and LCINS. There were also challenges with data extraction and synthesis. For example, many studies categorized continuous data. Therefore, meta-analyses were performed where this was appropriate and narrative synthesis used otherwise.

In summary, this study evaluated 192 risk factors and summarized and quantified key factors associated with LCINS worldwide, including female sex, rheumatoid arthritis, and pollution, for use in a potential tool to identify high-risk never-smokers for the early detection of LCINS.

## CRediT Authorship Contribution Statement

**Sindhu Bhaarrati Naidu**: Conceptualization, Methodology, Formal analysis, Investigation, Data curation, Writing - original draft, Writing - review & editing, Visualization, Project administration, Funding acquisition.

**Allegra Wisking**: Investigation, Writing - review & editing.

**Akul Karoshi**: Investigation, Writing - review & editing.

**Sarah Burdett**: Methodology, Formal analysis, Validation, Writing - review & editing, Visualization.

**Peter J. Godolphin**: Methodology, Formal analysis, Validation, Writing - review & editing, Visualization.

**Sanjay Popat**: Writing - review & editing.

**OLIVE PPI Group**: Conceptualization, Methodology, Writing - review & editing, Visualization.

**Sam M. Janes**: Resources, Writing - review & editing, Supervision, Funding acquisition.

**Neal Navani**: Conceptualization, Methodology, Validation, Resources, Writing - original draft, Writing - review & editing, Visualization, Supervision, Project administration, Funding acquisition.

## Disclosure

Popat has received consulting fees and/or is on advisory boards for AnHeart Therapeutics, Amgen, AstraZeneca, Bayer, Arcus Biosciences, Bristol Myers Squibb, Boehringer Ingelheim, Ellipses, Erasca, Daiichi Sankyo, Gilead, GlaxoSmithKline, Guardant Health, IO Biotech, Janssen, Lilly, Merck KGaA, Mirati, Merck Sharp & Dohme, Novocure, Novartis, Pharmamar, Roche, Sanofi, Takeda, Pfizer, Pierre Fabre, Turning Point Therapeutics, and Regeneron. He reports receiving honoraria from Amgen, AstraZeneca, Bayer, Gilead, Guardant Health, Janssen, Merck KGaA, Roche and Takeda. He has received travel expenses from Gilead. He had unpaid leadership roles in British Thoracic Oncology Group, ALK Positive UK, Lung Cancer Europe, Ruth Strauss Foundation, and ETOP-IBCSG Partners Foundation Board. Janes has received fees for advisory board membership in the last three years from Bard1 Lifescience. He has received grant income from GRAIL Inc. He is an unpaid member of a GRAIL advisory board. He has received lecture fees for academic meetings from Cheisi and AstraZeneca. His wife works for AstraZeneca. Navani reports receiving honoraria for non-promotional educational talks or advisory boards from Amgen, AstraZeneca, Boehringer Ingelheim, Bristol Myers Squibb, EQRx, Fujifilm, Guardant Health, Intuitive, Janssen, Lilly, Merck Sharp & Dohme, Sanofi, Olympus, and Roche. The remaining authors declare no conflict of interest.

## References

[1] Bray F., Laversanne M., Sung H. (2024). Global cancer statistics 2022: GLOBOCAN estimates of incidence and mortality worldwide for 36 cancers in 185 countries. CA Cancer J Clin.

[2] Sun S., Schiller J.H., Gazdar A.F. (2007). Lung cancer in never smokers — a different disease. Nat Rev Cancer.

[3] Kerpel-Fronius A., Tammemägi M., Cavic M. (2022). Screening for lung cancer in individuals who never smoked: an International Association for the Study of Lung Cancer early detection and screening committee report. J Thorac Oncol.

[4] National Lung Cancer Audit (Published Online 2024). State of the Nation 2024. https://www.natcan.org.uk/wp-content/uploads/2025/07/NLCA-State-of-the-Nation-2024_16.05.24_V2.0-1.pdf.

[5] Lyratzopoulos G., Neal R.D., Barbiere J.M., Rubin G.P., Abel G.A. (2012). Variation in number of general practitioner consultations before hospital referral for cancer: findings from the 2010 National Cancer Patient Experience Survey in England. Lancet Oncol.

[6] Dias M., Linhas R., Campainha S., Conde S., Barroso A. (2017). Lung cancer in never-smokers – what are the differences?. Acta Oncol.

[7] UK National Screening Committee (Published Online September 2022). Targeted Screening for Lung Cancer in Individuals at Increased Risk. https://view-health-screening-recommendations.service.gov.uk/lung-cancer/.

[8] Chang G.C., Chiu C.H., Yu C.J. (2024). Low-dose CT screening among never-smokers with or without a family history of lung cancer in Taiwan: a prospective cohort study. Lancet Respir Med.

[9] National Lung Screening Trial Research Team, Aberle D.R., Adams A.M. (2011). Reduced lung-cancer mortality with low-dose computed tomographic screening. N Engl J Med.

[10] De Koning H.J., Van Der Aalst C.M., De Jong P.A. (2020). Reduced lung-cancer mortality with volume CT screening in a randomized trial. N Engl J Med.

[11] Ten Haaf K ten, Van Der Aalst C.M., De Koning H.J., Kaaks R., Tammemägi M.C. (2021). Personalising lung cancer screening: an overview of risk-stratification opportunities and challenges. Int J Cancer.

[12] LoPiccolo J., Gusev A., Christiani D.C., Jänne P.A. (2024). Lung cancer in patients who have never smoked — an emerging disease. Nat Rev Clin Oncol.

[13] Zhang Z., Zhang X., Gao Y., Chen Y., Qin L., Wu I.X. (2022). Risk factors for the development of lung cancer among never smokers: a systematic review. Cancer Epidemiol.

[14] Shi J., Shiraishi K., Choi J. (2023). Genome-wide association study of lung adenocarcinoma in East Asia and comparison with a European population. Nat Commun.

[15] Naidu S.B., Navani N., Wisking A., Karoshi A., Burdett S. A systematic review of risk factors for lung cancer in never-smokers. CRD.York. https://www.crd.york.ac.uk/prospero/display_record.php?ID=CRD42022379253.

[16] Page M.J., McKenzie J.E., Bossuyt P.M. (2021). The PRISMA 2020 statement: an updated guideline for reporting systematic reviews. BMJ.

[17] Wells G., Shea B., O’Connell D. The Newcastle-Ottawa Scale (NOS) for assessing the quality of nonrandomised studies in meta-analyses. http://www.ohri.ca/programs/clinical_epidemiology/oxford.asp.

[18] Riley R.D., Moons K.G.M., Snell K.I.E. (2019). A guide to systematic review and meta-analysis of prognostic factor studies. BMJ.

[19] Tierney J.F., Stewart L.A., Ghersi D., Burdett S., Sydes M.R. (2007). Practical methods for incorporating summary time-to-event data into meta-analysis. Trials.

[20] IntHout J., Ioannidis J.P.A., Borm G.F. (2014). The Hartung-Knapp-Sidik-Jonkman method for random effects meta-analysis is straightforward and considerably outperforms the standard DerSimonian-Laird method. BMC Med Res Methodol.

[21] StataCorp (Published Online 2023). Stata statistical software. release 18. https://www.stata.com/.

[22] Staniszewska S., Brett J., Simera I. (2017). GRIPP2 reporting checklists: tools to improve reporting of patient and public involvement in research. BMJ.

[23] Cheng E.S., Weber M.F., Steinberg J., Canfell K., Yu X.Q. (2022). Evaluating risk factors for lung cancer among never-smoking individuals using two Australian studies. J Cancer Res Clin Oncol.

[24] Abdel-Rahman O. (2020). Incidence and mortality of lung cancer among never smokers in relationship to secondhand smoking: findings from the PLCO trial. Clin Lung Cancer.

[25] Warkentin M.T., Lam S., Hung R.J. (2019). Determinants of impaired lung function and lung cancer prediction among never-smokers in the UK Biobank cohort. EBioMedicine.

[26] Huang H.L., Chuang Y.H., Lin T.H. (2021). Ambient cumulative PM2.5 exposure and the risk of lung cancer incidence and mortality: a retrospective cohort study. Int J Environ Res Public Health.

[27] Yano Y., Abnet C.C., Roshandel G. (2024). Dental health and lung cancer risk in the Golestan Cohort Study. BMC Cancer.

[28] Kim Y.W., Joo D.H., Kim S.Y. (2025). Gender disparities and lung cancer screening outcomes among individuals who have never smoked. JAMA Netw Open.

[29] Jeon K.H., Shin D.W., Han K. (2020). Female reproductive factors and the risk of lung cancer in postmenopausal women: a nationwide cohort study. Br J Cancer.

[30] Yang Z., Wang F., Tan F. (2021). Menstrual factors, reproductive history, and risk of lung cancer: a multi-center population-based cohort study in Chinese females. Transl Lung Cancer Res.

[31] Wilunda C., Sawada N., Yamaji T., Iwasaki M., Inoue M., Tsugane S. (2021). Reproductive factors and lung cancer risk among never-smoking Japanese women with 21 years of follow-up: a cohort study. Cancer Epidemiol Biomarkers Prev.

[32] Gallet J., Laurent F., Paris C. (2022). Pleural plaques and risk of lung cancer in workers formerly occupationally exposed to asbestos: extension of follow-up. Occup Environ Med.

[33] Choi H., Park H.Y., Han K. (2022). Non–cystic fibrosis bronchiectasis increases the risk of lung cancer independent of smoking status. Ann Am Thorac Soc.

[34] Park H.Y., Kang D., Shin S.H. (2022). Pulmonary tuberculosis and the incidence of lung cancer among patients with chronic obstructive pulmonary disease. Ann Am Thorac Soc.

[35] Huang T., Lin B.M., Stampfer M.J. (2021). Associations of self-reported obstructive sleep apnea with total and site-specific cancer risk in older women: a prospective study. Sleep.

[36] Guo L., Meng Q., Zheng L. (2023). Lung cancer risk prediction nomogram in nonsmoking Chinese women: retrospective cross-sectional cohort study. JMIR Public Health Surveill.

[37] Chatzidionysiou K., Di Giuseppe D., Soderling J., Catrina A., Askling J. (2022). Risk of lung cancer in rheumatoid arthritis and in relation to autoantibody positivity and smoking. RMD Open.

[38] Cho M.H., Cho J.H., Eun Y. (2024). Rheumatoid arthritis and risk of lung cancer: a nationwide cohort study. J Thorac Oncol.

[39] Brooks R.T., Luedders B., Wheeler A. (2024). The risk of lung cancer in rheumatoid arthritis and rheumatoid arthritis–associated interstitial lung disease. Arthritis Rheumatol.

[40] Ma Z., Lv J., Zhu M. (2023). Lung cancer risk score for ever and never smokers in China. Cancer Commun (Lond).

[41] Ko Y.H., Kim S.J., Kim W.S. (2020). Risk factors for primary lung cancer among never-smoking women in South Korea: a retrospective nationwide population-based cohort study. Korean J Intern Med.

[42] Wu Z., Tan F., Yang Z. (2022). Sex disparity of lung cancer risk in non-smokers: a multicenter population-based prospective study based on China National Lung Cancer Screening Program. Chin Med J (Engl).

[43] Nohara S., Mok Y., Van’t Hof J.R. (2025). Subsequent risk of cancer among adults with peripheral artery disease in the community: the atherosclerosis risk in communities (ARIC) study. Int J Cardiol.

[44] Hosgood H.D., Cai Q., Hua X. (2021). Variation in oral microbiome is associated with future risk of lung cancer among never-smokers. Thorax.

[45] Moon S., Lee H.Y., Jang J., Park S.K. (2020). Association between angiotensin II receptor blockers and the risk of lung cancer among patients with hypertension from the Korean National Health Insurance Service-national health screening cohort. J Prev Med Public Health.

[46] Tao M.H., Chen S., Freudenheim J.L. (2018). Oral bisphosphonate use and lung cancer incidence among postmenopausal women. Ann Oncol.

[47] Kang J., Jeong S.M., Shin D.W., Cho M., Cho J.H., Kim J. (2021). The associations of Aspirin, statins, and metformin with lung cancer risk and related mortality: a time-dependent analysis of population-based nationally representative data. J Thorac Oncol.

[48] Wang F., Tan F., Wu Z. (2021). Lung cancer risk in non-smoking females with a familial history of cancer: a multi-center prospective cohort study in China. J Natl Cancer Cent.

[49] Erhunmwunsee L., Wing S.E., Zou X., Coogan P., Palmer J.R., Lennie Wong F. (2022). Neighborhood disadvantage and lung cancer risk in a national cohort of never smoking Black women. Lung Cancer.

[50] Rait G., Horsfall L. (2020). Twenty-year sociodemographic trends in lung cancer in non-smokers: a UK-based cohort study of 3.7 million people. Cancer Epidemiol.

[51] DeRouen M.C., Canchola A.J., Thompson C.A. (2022). Incidence of lung cancer among never-smoking Asian American, Native Hawaiian, and Pacific islander females. J Natl Cancer Inst.

[52] Wu Z., Xie S., Wang F. (2022). BMI changes and the risk of lung cancer in male never-smokers: a prospective cohort study. Cancer Med.

[53] Murphy R.A., Darvishian M., Qi J. (2022). Lifestyle factors and lung cancer risk among never smokers in the Canadian Partnership for Tomorrow’s Health (CanPath). Cancer Causes Control.

[54] Hansen M.S., Licaj I., Braaten T., Lund E., Gram I.T. (2021). The fraction of lung cancer attributable to smoking in the Norwegian Women and Cancer (NOWAC) Study. Br J Cancer.

[55] Li J., Xu H.L., Yao B.D. (2020). Environmental tobacco smoke and cancer risk, a prospective cohort study in a Chinese population. Environ Res.

[56] Lee H.W., Kang S.C., Kim S.Y., Cho Y.J., Hwang S. (2022). Long-term exposure to PM10 increases lung cancer risks: a cohort analysis. Cancer Res Treat.

[57] Huang Y., Zhu M., Ji M. (2021). Air pollution, genetic factors, and the risk of lung cancer: a prospective study in the UK Biobank. Am J Respir Crit Care Med.

[58] Gowda S.N., DeRoos A.J., Hunt R.P. (2019). Ambient air pollution and lung cancer risk among never-smokers in the Women’s Health Initiative. Environ Epidemiol.

[59] Yang S., Kim O.J., Shin M., Kim W.J., Kim S.Y. (2021). Association between long-term exposure to high levels of ambient air pollution and incidence of lung cancer in a population-based cohort. Environ Res.

[60] Gharibvand L., Shavlik D., Ghamsary M. (2017). The association between ambient fine particulate air pollution and lung cancer incidence: results from the AHSMOG-2 study. Environ Health Perspect.

[61] Zhu M., Han Y., Mou Y. (2025). Effect of long-term fine particulate matter exposure on lung cancer incidence and mortality in Chinese nonsmokers. Am J Respir Crit Care Med.

[62] Bonner M.R., Freeman L.E.B., Hoppin J.A. (2017). Occupational exposure to pesticides and the incidence of lung cancer in the agricultural health study. Environ Health Perspect.

[63] Park S.Y., Boushey C.J., Shvetsov Y.B. (2021). Diet quality and risk of lung cancer in the multiethnic cohort study. Nutrients.

[64] Myneni A.A., Giovino G.A., Millen A.E. (2021). Indices of diet quality and risk of lung cancer in the Women’s Health Initiative observational study. J Nutr.

[65] Wong J.Y.Y., Bassig B.A., Loftfield E. (2020). White blood cell count and risk of incident lung cancer in the UK Biobank. JNCI Cancer Spectr.

[66] Christakoudi S., Tsilidis K.K., Evangelou E., Riboli E. (2023). Interactions of platelets with obesity in relation to lung cancer risk in the UK Biobank cohort. Respir Res.

[67] Lyu Z., Li N., Wang G. (2019). Independent and joint associations of blood lipids and lipoproteins with lung cancer risk in Chinese males: a prospective cohort study. Int J Cancer.

[68] Wei Y., Xu B., He Q. (2023). Serum total folate, 5-methyltetrahydrofolate and vitamin B12 concentrations on incident risk of lung cancer. Int J Cancer.

[69] Shiels M.S., Shu X.O., Chaturvedi A.K. (2017). A prospective study of immune and inflammation markers and risk of lung cancer among female never smokers in Shanghai. Carcinogenesis.

[70] Yuan J.M., Beckman K.B., Wang R. (2018). Leukocyte telomere length in relation to risk of lung adenocarcinoma incidence: findings from the Singapore Chinese Health Study. Int J Cancer.

[71] Wong J.Y.Y., Shu X.O., Hu W. (2023). Associations between longer leukocyte telomere length and increased lung cancer risk among never smokers in urban China. Cancer Epidemiol Biomarkers Prev.

[72] Han D., Zhu Y., Choudhry A.A. (2023). Association of telomere length with risk of lung cancer: a large prospective cohort study from the UK Biobank. Lung Cancer.

[73] Sandanger T.M., Nøst T.H., Guida F. (2018). DNA methylation and associated gene expression in blood prior to lung cancer diagnosis in the Norwegian Women and Cancer cohort. Sci Rep.

[74] Seow W.J., Shu X.O., Nicholson J.K. (2019). Association of Untargeted Urinary Metabolomics and Lung Cancer Risk Among Never-Smoking Women in China. JAMA Netw Open.

[75] Li M., Cai Q., Gao Y.T. (2022). Phytoestrogens and lung cancer risk: a nested case-control study in never-smoking Chinese women. Am J Clin Nutr.

[76] Mao W., Qu J., Liu H. (2024). Associations between urinary concentrations of benzothiazole, benzotriazole, and their derivatives and lung cancer: a nested case-control study. Environ Res.

[77] Recalde M., Davila-Batista V., Díaz Y. (2021). Body mass index and waist circumference in relation to the risk of 26 types of cancer: a prospective cohort study of 3.5 million adults in Spain. BMC Med.

[78] Mehta S.S., Elizabeth Hodgson M., Lunn R.M. (2023). Indoor wood-burning from stoves and fireplaces and incident lung cancer among Sister Study participants. Environ Int.

[79] Du Y., Sidorenkov G., Groen H.J.M. (2022). Airflow limitation increases lung cancer risk in smokers: the lifelines cohort study. Cancer Epidemiol Biomarkers Prev.

[80] Park H.Y., Kang D., Shin S.H. (2020). Chronic obstructive pulmonary disease and lung cancer incidence in never smokers: a cohort study. Thorax.

[81] Kyaw T.W., Tsai M.K., Wen C.P. (2024). Impaired lung function and lung cancer risk in 461 183 healthy individuals: a cohort study. BMJ Open Respir Res.

[82] Delcoigne B., Colzani E., Prochazka M. (2017). Breaking the matching in nested case–control data offered several advantages for risk estimation. J Clin Epidemiol.

[83] DiMarzio P., Peila R., Dowling O. (2018). Smoking and alcohol drinking effect on radiotherapy associated risk of second primary cancer and mortality among breast cancer patients. Cancer Epidemiol.

[84] Lee Y.G., Seo D., Gil H.I. (2025). Assessment of lung cancer risks related to family history in never-smokers: a cohort study. Clin Lung Cancer.

[85] He H., He M.M., Wang H. (2023). *In utero* and childhood/adolescence exposure to tobacco smoke, genetic risk, and lung cancer incidence and mortality in adulthood. Am J Respir Crit Care Med.

[86] Kelly-Reif K., Sandler D.P., Shore D. (2022). Lung and extrathoracic cancer incidence among underground uranium miners exposed to radon progeny in the Příbram region of the Czech Republic: a case–cohort study. Occup Environ Med.

[87] Cheng T.D., Song X., Beresford S.A.A. (2017). Serum 25-hydroxyvitamin D concentrations and lung cancer risk in never-smoking postmenopausal women. Cancer Causes Control.

[88] Weng C., Zhao Y., Song M. (2024). Mosaic loss of chromosome Y, tobacco smoking and risk of age-related lung diseases: insights from two prospective cohorts. Eur Respir J.

[89] Tian R., Wiley B., Liu J. (2023). Clonal hematopoiesis and risk of incident lung cancer. J Clin Oncol.

[90] McGuinness L.A., Higgins J.P.T. (2021). Risk-of-bias VISualization (robvis): an R package and Shiny web app for visualizing risk-of-bias assessments. Res Synth Methods.

[91] Black G.B., Janes S.M., Callister M.E.J., Van Os S., Whitaker K.L., Quaife S.L. (2024). The role of smoking status in making risk-informed diagnostic decisions in the lung cancer pathway: a qualitative study of health care professionals and patients. Med Decis Making.

[92] Afriyie-Mensah J.S., Kwarteng E., Tetteh J. (2023). A three-year review of lung cancer patient characteristics in a tertiary hospital. Ghana Med J.

[93] Riley R.D., van der Windt D., Croft P., Moons K.G.M. (2019). Prognosis Research in Healthcare: Concepts, Methods, and Impact.

[94] Pinheiro P.S., Callahan K.E., Medina H.N. (2022). Lung cancer in never smokers: distinct population-based patterns by age, sex, and race/ethnicity. Lung Cancer.

