# Supplementary material for: Risk Factors Associated With Incidence of Lung Cancer in Never-Smokers: A Systematic Review and Meta-Analysis
Source: JTO Clin Res Rep. 2025 Sep 18;6(12):100910. doi: 10.1016/j.jtocrr.2025.100910 (PMC12639266; doi:10.1016/j.jtocrr.2025.100910)
Supplement: Supplementary Appendix [file mmc1.docx]

**Online Data Supplement**

**Risk factors associated with incidence of lung cancer in never-smokers: a systematic review and meta-analysis**

**Authors**

Sindhu Bhaarrati Naidu, MBBS

Allegra Wisking, BSc

Akul Karoshi, BSc

Sarah Burdett, MSc

Peter J Godolphin, PhD

Sanjay Popat, PhD

OLIVE PPI Group

Sam M Janes, PhD

Neal Navani, PhD

[Appendix 1: Search strategy 3](#_Toc200961234)

[Appendix 2: Data extraction form 14](#_Toc200961235)

[Appendix 3: Studies reviewing association of risk factors with outcome of lung cancer in never-smokers 16](#_Toc200961236)

[Appendix 4: Summary of meta-analyses 45](#_Toc200961237)

[Appendix 5: Effect measures for all factors and studies 47](#_Toc200961238)

[Appendix 6: Factors associated with medication, dietary intake and urine laboratory tests 65](#_Toc200961239)

[Appendix 7: Subgroup analyses 66](#_Toc200961240)

[Appendix 8: Summary of risk of bias 68](#_Toc200961241)

[References for appendices 73](#_Toc200961242)

# Appendix 1: Search strategy

| **Search strategy used, including any limits** | | **Number of results** | **Repeat search** |
| --- | --- | --- | --- |
| **Embase (13/01/23 and 20/03/25)** | | |  |
| 1 | (lung adj3 neoplasm*).mp. [mp=title, abstract, heading word, drug trade name, original title, device manufacturer, drug manufacturer, device trade name, keyword heading word, floating subheading word, candidate term word] | 12902 | 10710 |
| 2 | (lung adj3 cancer*).mp. [mp=title, abstract, heading word, drug trade name, original title, device manufacturer, drug manufacturer, device trade name, keyword heading word, floating subheading word, candidate term word] | 390645 | 478572 |
| 3 | (lung adj3 carcinoma*).mp. [mp=title, abstract, heading word, drug trade name, original title, device manufacturer, drug manufacturer, device trade name, keyword heading word, floating subheading word, candidate term word] | 77948 | 91472 |
| 4 | (lung adj3 tumor*).mp. [mp=title, abstract, heading word, drug trade name, original title, device manufacturer, drug manufacturer, device trade name, keyword heading word, floating subheading word, candidate term word] | 87106 | 102696 |
| 5 | (lung adj3 tumour*).mp. [mp=title, abstract, heading word, drug trade name, original title, device manufacturer, drug manufacturer, device trade name, keyword heading word, floating subheading word, candidate term word] | 5642 | 6419 |
| 6 | (lung adj3 malignanc*).mp. [mp=title, abstract, heading word, drug trade name, original title, device manufacturer, drug manufacturer, device trade name, keyword heading word, floating subheading word, candidate term word] | 5211 | 6370 |
| 7 | (lung adj3 adenocarcinoma*).mp. [mp=title, abstract, heading word, drug trade name, original title, device manufacturer, drug manufacturer, device trade name, keyword heading word, floating subheading word, candidate term word] | 62188 | 76703 |
| 8 | (lung adj3 carcinogenesis).mp. [mp=title, abstract, heading word, drug trade name, original title, device manufacturer, drug manufacturer, device trade name, keyword heading word, floating subheading word, candidate term word] | 8903 | 9846 |
| 9 | (lung adj3 adenoma*).mp. [mp=title, abstract, heading word, drug trade name, original title, device manufacturer, drug manufacturer, device trade name, keyword heading word, floating subheading word, candidate term word] | 1938 | 2389 |
| 10 | (pulmonary adj3 neoplasm*).mp. [mp=title, abstract, heading word, drug trade name, original title, device manufacturer, drug manufacturer, device trade name, keyword heading word, floating subheading word, candidate term word] | 1375 | 1610 |
| 11 | (pulmonary adj3 cancer*).mp. [mp=title, abstract, heading word, drug trade name, original title, device manufacturer, drug manufacturer, device trade name, keyword heading word, floating subheading word, candidate term word] | 5811 | 6945 |
| 12 | (pulmonary adj3 carcinoma*).mp. [mp=title, abstract, heading word, drug trade name, original title, device manufacturer, drug manufacturer, device trade name, keyword heading word, floating subheading word, candidate term word] | 4687 | 5611 |
| 13 | (pulmonary adj3 tumor*).mp. [mp=title, abstract, heading word, drug trade name, original title, device manufacturer, drug manufacturer, device trade name, keyword heading word, floating subheading word, candidate term word] | 6459 | 7383 |
| 14 | (pulmonary adj3 tumour*).mp. [mp=title, abstract, heading word, drug trade name, original title, device manufacturer, drug manufacturer, device trade name, keyword heading word, floating subheading word, candidate term word] | 1140 | 1319 |
| 15 | (pulmonary adj3 malignanc*).mp. [mp=title, abstract, heading word, drug trade name, original title, device manufacturer, drug manufacturer, device trade name, keyword heading word, floating subheading word, candidate term word] | 2233 | 2726 |
| 16 | (pulmonary adj3 adenocarcinoma*).mp. [mp=title, abstract, heading word, drug trade name, original title, device manufacturer, drug manufacturer, device trade name, keyword heading word, floating subheading word, candidate term word] | 4067 | 4602 |
| 17 | (pulmonary adj3 carcinogenesis).mp. [mp=title, abstract, heading word, drug trade name, original title, device manufacturer, drug manufacturer, device trade name, keyword heading word, floating subheading word, candidate term word] | 281 | 307 |
| 18 | (pulmonary adj3 adenoma*).mp. [mp=title, abstract, heading word, drug trade name, original title, device manufacturer, drug manufacturer, device trade name, keyword heading word, floating subheading word, candidate term word] | 582 | 714 |
| 19 | (bronchopulmonary adj3 neoplasm*).mp. [mp=title, abstract, heading word, drug trade name, original title, device manufacturer, drug manufacturer, device trade name, keyword heading word, floating subheading word, candidate term word] | 75 | 88 |
| 20 | (bronchopulmonary adj3 cancer*).mp. [mp=title, abstract, heading word, drug trade name, original title, device manufacturer, drug manufacturer, device trade name, keyword heading word, floating subheading word, candidate term word] | 302 | 419 |
| 21 | (bronchopulmonary adj3 carcinoma*).mp. [mp=title, abstract, heading word, drug trade name, original title, device manufacturer, drug manufacturer, device trade name, keyword heading word, floating subheading word, candidate term word] | 187 | 235 |
| 22 | (bronchopulmonary adj3 tumor*).mp. [mp=title, abstract, heading word, drug trade name, original title, device manufacturer, drug manufacturer, device trade name, keyword heading word, floating subheading word, candidate term word] | 249 | 299 |
| 23 | (bronchopulmonary adj3 tumour*).mp. [mp=title, abstract, heading word, drug trade name, original title, device manufacturer, drug manufacturer, device trade name, keyword heading word, floating subheading word, candidate term word] | 71 | 87 |
| 24 | (bronchopulmonary adj3 malignanc*).mp. [mp=title, abstract, heading word, drug trade name, original title, device manufacturer, drug manufacturer, device trade name, keyword heading word, floating subheading word, candidate term word] | 18 | 22 |
| 25 | (bronchopulmonary adj3 adenocarcinoma*).mp. [mp=title, abstract, heading word, drug trade name, original title, device manufacturer, drug manufacturer, device trade name, keyword heading word, floating subheading word, candidate term word] | 20 | 26 |
| 26 | (bronchopulmonary adj3 carcinogenesis).mp. [mp=title, abstract, heading word, drug trade name, original title, device manufacturer, drug manufacturer, device trade name, keyword heading word, floating subheading word, candidate term word] | 2 | 2 |
| 27 | (bronchopulmonary adj3 adenoma*).mp. [mp=title, abstract, heading word, drug trade name, original title, device manufacturer, drug manufacturer, device trade name, keyword heading word, floating subheading word, candidate term word] | 45 | 50 |
| 28 | (bronchial adj3 neoplasm*).mp. [mp=title, abstract, heading word, drug trade name, original title, device manufacturer, drug manufacturer, device trade name, keyword heading word, floating subheading word, candidate term word] | 260 | 282 |
| 29 | (bronchial adj3 cancer*).mp. [mp=title, abstract, heading word, drug trade name, original title, device manufacturer, drug manufacturer, device trade name, keyword heading word, floating subheading word, candidate term word] | 1396 | 1831 |
| 30 | (bronchial adj3 carcinoma*).mp. [mp=title, abstract, heading word, drug trade name, original title, device manufacturer, drug manufacturer, device trade name, keyword heading word, floating subheading word, candidate term word] | 3129 | 4145 |
| 31 | (bronchial adj3 tumor*).mp. [mp=title, abstract, heading word, drug trade name, original title, device manufacturer, drug manufacturer, device trade name, keyword heading word, floating subheading word, candidate term word] | 1487 | 1704 |
| 32 | (bronchial adj3 tumour*).mp. [mp=title, abstract, heading word, drug trade name, original title, device manufacturer, drug manufacturer, device trade name, keyword heading word, floating subheading word, candidate term word] | 394 | 454 |
| 33 | (bronchial adj3 malignanc*).mp. [mp=title, abstract, heading word, drug trade name, original title, device manufacturer, drug manufacturer, device trade name, keyword heading word, floating subheading word, candidate term word] | 133 | 157 |
| 34 | (bronchial adj3 adenocarcinoma*).mp. [mp=title, abstract, heading word, drug trade name, original title, device manufacturer, drug manufacturer, device trade name, keyword heading word, floating subheading word, candidate term word] | 192 | 210 |
| 35 | (bronchial adj3 carcinogenesis).mp. [mp=title, abstract, heading word, drug trade name, original title, device manufacturer, drug manufacturer, device trade name, keyword heading word, floating subheading word, candidate term word] | 112 | 116 |
| 36 | (bronchial adj3 adenoma*).mp. [mp=title, abstract, heading word, drug trade name, original title, device manufacturer, drug manufacturer, device trade name, keyword heading word, floating subheading word, candidate term word] | 193 | 320 |
| 37 | (bronchogenic adj3 neoplasm*).mp. [mp=title, abstract, heading word, drug trade name, original title, device manufacturer, drug manufacturer, device trade name, keyword heading word, floating subheading word, candidate term word] | 55 | 68 |
| 38 | (bronchogenic adj3 cancer*).mp. [mp=title, abstract, heading word, drug trade name, original title, device manufacturer, drug manufacturer, device trade name, keyword heading word, floating subheading word, candidate term word] | 386 | 461 |
| 39 | (bronchogenic adj3 carcinoma*).mp. [mp=title, abstract, heading word, drug trade name, original title, device manufacturer, drug manufacturer, device trade name, keyword heading word, floating subheading word, candidate term word] | 4213 | 5105 |
| 40 | 40 (bronchogenic adj3 tumor*).mp. [mp=title, abstract, heading word, drug trade name, original title, device manufacturer, drug manufacturer, device trade name, keyword heading word, floating subheading word, candidate term word] | 159 | 194 |
| 41 | (bronchogenic adj3 tumour*).mp. [mp=title, abstract, heading word, drug trade name, original title, device manufacturer, drug manufacturer, device trade name, keyword heading word, floating subheading word, candidate term word] | 48 | 63 |
| 42 | (bronchogenic adj3 malignanc*).mp. [mp=title, abstract, heading word, drug trade name, original title, device manufacturer, drug manufacturer, device trade name, keyword heading word, floating subheading word, candidate term word] | 81 | 92 |
| 43 | (bronchogenic adj3 adenocarcinoma*).mp. [mp=title, abstract, heading word, drug trade name, original title, device manufacturer, drug manufacturer, device trade name, keyword heading word, floating subheading word, candidate term word] | 137 | 158 |
| 44 | (bronchogenic adj3 carcinogenesis).mp. [mp=title, abstract, heading word, drug trade name, original title, device manufacturer, drug manufacturer, device trade name, keyword heading word, floating subheading word, candidate term word] | 11 | 11 |
| 45 | (bronchogenic adj3 adenoma*).mp. [mp=title, abstract, heading word, drug trade name, original title, device manufacturer, drug manufacturer, device trade name, keyword heading word, floating subheading word, candidate term word] | 27 | 31 |
| 46 | exp lung tumor/ | 444706 | 592527 |
| 47 | 1 or 2 or 3 or 4 or 5 or 6 or 7 or 8 or 9 or 10 or 11 or 12 or 13 or 14 or 15 or 16 or 17 or 18 or 19 or 20 or 21 or 22 or 23 or 24 or 25 or 26 or 27 or 28 or 29 or 30 or 31 or 32 or 33 or 34 or 35 or 36 or 37 or 38 or 39 or 40 or 41 or 42 or 43 or 44 or 45 or 46 | 551017 | 664807 |
| 48 | exp risk factor/ | 1278243 | 1523492 |
| 49 | risk*.mp. [mp=title, abstract, heading word, drug trade name, original title, device manufacturer, drug manufacturer, device trade name, keyword heading word, floating subheading word, candidate term word] | 4809647 | 5675799 |
| 50 | epidemiolog*.mp. [mp=title, abstract, heading word, drug trade name, original title, device manufacturer, drug manufacturer, device trade name, keyword heading word, floating subheading word, candidate term word] | 1724000 | 1960528 |
| 51 | incidence.mp. [mp=title, abstract, heading word, drug trade name, original title, device manufacturer, drug manufacturer, device trade name, keyword heading word, floating subheading word, candidate term word] | 1412085 | 1668558 |
| 52 | prevalence.mp. [mp=title, abstract, heading word, drug trade name, original title, device manufacturer, drug manufacturer, device trade name, keyword heading word, floating subheading word, candidate term word] | 1299725 | 1554683 |
| 53 | (health adj3 correlate*).mp. [mp=title, abstract, heading word, drug trade name, original title, device manufacturer, drug manufacturer, device trade name, keyword heading word, floating subheading word, candidate term word] | 4841 | 5771 |
| 54 | associat*.mp. [mp=title, abstract, heading word, drug trade name, original title, device manufacturer, drug manufacturer, device trade name, keyword heading word, floating subheading word, candidate term word] | 7647651 | 8953156 |
| 55 | 48 or 49 or 50 or 51 or 52 or 53 or 54 | 11941407 | 13966321 |
| 56 | non-smok*.mp. [mp=title, abstract, heading word, drug trade name, original title, device manufacturer, drug manufacturer, device trade name, keyword heading word, floating subheading word, candidate term word] | 41274 | 48794 |
| 57 | never-smok*.mp. [mp=title, abstract, heading word, drug trade name, original title, device manufacturer, drug manufacturer, device trade name, keyword heading word, floating subheading word, candidate term word] | 20098 | 24033 |
| 58 | nonsmok*.mp. [mp=title, abstract, heading word, drug trade name, original title, device manufacturer, drug manufacturer, device trade name, keyword heading word, floating subheading word, candidate term word] | 25438 | 26700 |
| 59 | "never smok*".mp. [mp=title, abstract, heading word, drug trade name, original title, device manufacturer, drug manufacturer, device trade name, keyword heading word, floating subheading word, candidate term word] | 20098 | 24033 |
| 60 | "non smok*".mp. [mp=title, abstract, heading word, drug trade name, original title, device manufacturer, drug manufacturer, device trade name, keyword heading word, floating subheading word, candidate term word] | 41274 | 48794 |
| 61 | "non-current smok*".mp. [mp=title, abstract, heading word, drug trade name, original title, device manufacturer, drug manufacturer, device trade name, keyword heading word, floating subheading word, candidate term word] | 149 | 175 |
| 62 | "non current smok*".mp. [mp=title, abstract, heading word, drug trade name, original title, device manufacturer, drug manufacturer, device trade name, keyword heading word, floating subheading word, candidate term word] | 149 | 175 |
| 63 | exp never smoker/ | 3709 | 6533 |
| 64 | 56 or 57 or 58 or 59 or 60 or 61 or 62 or 63 | 79516 | 91248 |
| 65 | 47 and 55 and 64 | 9070 | 10676 |
| 66 | limit 65 to (english language and yr="2015 -Current") | 4288 | 4848 |
| 67 | 66 not ((exp animal/ or nonhuman/) not exp human/) | 4262 | 4818 |
| **Ovid MEDLINE(R) ALL (including Pubmed) (16/01/23 and 20/03/25)** | | |  |
| 1 | (lung adj3 neoplasm*).mp. [mp=title, book title, abstract, original title, name of substance word, subject heading word, floating sub-heading word, keyword heading word, organism supplementary concept word, protocol supplementary concept word, rare disease supplementary concept word, unique identifier, synonyms] | 253304 | 279039 |
| 2 | (lung adj3 cancer*).mp. [mp=title, book title, abstract, original title, name of substance word, subject heading word, floating sub-heading word, keyword heading word, organism supplementary concept word, protocol supplementary concept word, rare disease supplementary concept word, unique identifier, synonyms] | 208141 | 244507 |
| 3 | (lung adj3 carcinoma*).mp. [mp=title, book title, abstract, original title, name of substance word, subject heading word, floating sub-heading word, keyword heading word, organism supplementary concept word, protocol supplementary concept word, rare disease supplementary concept word, unique identifier, synonyms] | 41165 | 45650 |
| 4 | (lung adj3 tumor*).mp. [mp=title, book title, abstract, original title, name of substance word, subject heading word, floating sub-heading word, keyword heading word, organism supplementary concept word, protocol supplementary concept word, rare disease supplementary concept word, unique identifier, synonyms] | 26035 | 29351 |
| 5 | (lung adj3 tumour*).mp. [mp=title, book title, abstract, original title, name of substance word, subject heading word, floating sub-heading word, keyword heading word, organism supplementary concept word, protocol supplementary concept word, rare disease supplementary concept word, unique identifier, synonyms] | 3800 | 4111 |
| 6 | (lung adj3 malignanc*).mp. [mp=title, book title, abstract, original title, name of substance word, subject heading word, floating sub-heading word, keyword heading word, organism supplementary concept word, protocol supplementary concept word, rare disease supplementary concept word, unique identifier, synonyms] | 2809 | 3324 |
| 7 | (lung adj3 adenocarcinoma*).mp. [mp=title, book title, abstract, original title, name of substance word, subject heading word, floating sub-heading word, keyword heading word, organism supplementary concept word, protocol supplementary concept word, rare disease supplementary concept word, unique identifier, synonyms] | 29177 | 36006 |
| 8 | (lung adj3 carcinogenesis).mp. [mp=title, book title, abstract, original title, name of substance word, subject heading word, floating sub-heading word, keyword heading word, organism supplementary concept word, protocol supplementary concept word, rare disease supplementary concept word, unique identifier, synonyms] | 2245 | 2424 |
| 9 | (lung adj3 adenoma*).mp. [mp=title, book title, abstract, original title, name of substance word, subject heading word, floating sub-heading word, keyword heading word, organism supplementary concept word, protocol supplementary concept word, rare disease supplementary concept word, unique identifier, synonyms] | 1854 | 1945 |
| 10 | (pulmonary adj3 neoplasm*).mp. [mp=title, book title, abstract, original title, name of substance word, subject heading word, floating sub-heading word, keyword heading word, organism supplementary concept word, protocol supplementary concept word, rare disease supplementary concept word, unique identifier, synonyms] | 1187 | 1264 |
| 11 | (pulmonary adj3 cancer*).mp. [mp=title, book title, abstract, original title, name of substance word, subject heading word, floating sub-heading word, keyword heading word, organism supplementary concept word, protocol supplementary concept word, rare disease supplementary concept word, unique identifier, synonyms] | 4777 | 5359 |
| 12 | (pulmonary adj3 carcinoma*).mp. [mp=title, book title, abstract, original title, name of substance word, subject heading word, floating sub-heading word, keyword heading word, organism supplementary concept word, protocol supplementary concept word, rare disease supplementary concept word, unique identifier, synonyms] | 3935 | 4326 |
| 13 | (pulmonary adj3 tumor*).mp. [mp=title, book title, abstract, original title, name of substance word, subject heading word, floating sub-heading word, keyword heading word, organism supplementary concept word, protocol supplementary concept word, rare disease supplementary concept word, unique identifier, synonyms] | 5071 | 5519 |
| 14 | (pulmonary adj3 tumour*).mp. [mp=title, book title, abstract, original title, name of substance word, subject heading word, floating sub-heading word, keyword heading word, organism supplementary concept word, protocol supplementary concept word, rare disease supplementary concept word, unique identifier, synonyms] | 840 | 887 |
| 15 | (pulmonary adj3 malignanc*).mp. [mp=title, book title, abstract, original title, name of substance word, subject heading word, floating sub-heading word, keyword heading word, organism supplementary concept word, protocol supplementary concept word, rare disease supplementary concept word, unique identifier, synonyms] | 1331 | 1558 |
| 16 | (pulmonary adj3 adenocarcinoma*).mp. [mp=title, book title, abstract, original title, name of substance word, subject heading word, floating sub-heading word, keyword heading word, organism supplementary concept word, protocol supplementary concept word, rare disease supplementary concept word, unique identifier, synonyms] | 2817 | 3153 |
| 17 | (pulmonary adj3 carcinogenesis).mp. [mp=title, book title, abstract, original title, name of substance word, subject heading word, floating sub-heading word, keyword heading word, organism supplementary concept word, protocol supplementary concept word, rare disease supplementary concept word, unique identifier, synonyms] | 246 | 256 |
| 18 | (pulmonary adj3 adenoma*).mp. [mp=title, book title, abstract, original title, name of substance word, subject heading word, floating sub-heading word, keyword heading word, organism supplementary concept word, protocol supplementary concept word, rare disease supplementary concept word, unique identifier, synonyms] | 1025 | 1051 |
| 19 | (bronchopulmonary adj3 neoplasm*).mp. [mp=title, book title, abstract, original title, name of substance word, subject heading word, floating sub-heading word, keyword heading word, organism supplementary concept word, protocol supplementary concept word, rare disease supplementary concept word, unique identifier, synonyms] | 82 | 88 |
| 20 | (bronchopulmonary adj3 cancer*).mp. [mp=title, book title, abstract, original title, name of substance word, subject heading word, floating sub-heading word, keyword heading word, organism supplementary concept word, protocol supplementary concept word, rare disease supplementary concept word, unique identifier, synonyms] | 495 | 510 |
| 21 | (bronchopulmonary adj3 carcinoma*).mp. [mp=title, book title, abstract, original title, name of substance word, subject heading word, floating sub-heading word, keyword heading word, organism supplementary concept word, protocol supplementary concept word, rare disease supplementary concept word, unique identifier, synonyms] | 178 | 181 |
| 22 | (bronchopulmonary adj3 tumor*).mp. [mp=title, book title, abstract, original title, name of substance word, subject heading word, floating sub-heading word, keyword heading word, organism supplementary concept word, protocol supplementary concept word, rare disease supplementary concept word, unique identifier, synonyms] | 214 | 233 |
| 23 | (bronchopulmonary adj3 tumour*).mp. [mp=title, book title, abstract, original title, name of substance word, subject heading word, floating sub-heading word, keyword heading word, organism supplementary concept word, protocol supplementary concept word, rare disease supplementary concept word, unique identifier, synonyms] | 55 | 56 |
| 24 | (bronchopulmonary adj3 malignanc*).mp. [mp=title, book title, abstract, original title, name of substance word, subject heading word, floating sub-heading word, keyword heading word, organism supplementary concept word, protocol supplementary concept word, rare disease supplementary concept word, unique identifier, synonyms] | 12 | 13 |
| 25 | (bronchopulmonary adj3 adenocarcinoma*).mp. [mp=title, book title, abstract, original title, name of substance word, subject heading word, floating sub-heading word, keyword heading word, organism supplementary concept word, protocol supplementary concept word, rare disease supplementary concept word, unique identifier, synonyms] | 14 | 15 |
| 26 | (bronchopulmonary adj3 carcinogenesis).mp. [mp=title, book title, abstract, original title, name of substance word, subject heading word, floating sub-heading word, keyword heading word, organism supplementary concept word, protocol supplementary concept word, rare disease supplementary concept word, unique identifier, synonyms] | 1 | 1 |
| 27 | (bronchopulmonary adj3 adenoma*).mp. [mp=title, book title, abstract, original title, name of substance word, subject heading word, floating sub-heading word, keyword heading word, organism supplementary concept word, protocol supplementary concept word, rare disease supplementary concept word, unique identifier, synonyms] | 37 | 38 |
| 28 | (bronchial adj3 neoplasm*).mp. [mp=title, book title, abstract, original title, name of substance word, subject heading word, floating sub-heading word, keyword heading word, organism supplementary concept word, protocol supplementary concept word, rare disease supplementary concept word, unique identifier, synonyms] | 12005 | 12099 |
| 29 | (bronchial adj3 cancer*).mp. [mp=title, book title, abstract, original title, name of substance word, subject heading word, floating sub-heading word, keyword heading word, organism supplementary concept word, protocol supplementary concept word, rare disease supplementary concept word, unique identifier, synonyms] | 2255 | 2322 |
| 30 | (bronchial adj3 carcinoma*).mp. [mp=title, book title, abstract, original title, name of substance word, subject heading word, floating sub-heading word, keyword heading word, organism supplementary concept word, protocol supplementary concept word, rare disease supplementary concept word, unique identifier, synonyms] | 4280 | 4314 |
| 31 | (bronchial adj3 tumor*).mp. [mp=title, book title, abstract, original title, name of substance word, subject heading word, floating sub-heading word, keyword heading word, organism supplementary concept word, protocol supplementary concept word, rare disease supplementary concept word, unique identifier, synonyms] | 1305 | 1380 |
| 32 | (bronchial adj3 tumour*).mp. [mp=title, book title, abstract, original title, name of substance word, subject heading word, floating sub-heading word, keyword heading word, organism supplementary concept word, protocol supplementary concept word, rare disease supplementary concept word, unique identifier, synonyms] | 350 | 360 |
| 33 | (bronchial adj3 malignanc*).mp. [mp=title, book title, abstract, original title, name of substance word, subject heading word, floating sub-heading word, keyword heading word, organism supplementary concept word, protocol supplementary concept word, rare disease supplementary concept word, unique identifier, synonyms] | 89 | 96 |
| 34 | (bronchial adj3 adenocarcinoma*).mp. [mp=title, book title, abstract, original title, name of substance word, subject heading word, floating sub-heading word, keyword heading word, organism supplementary concept word, protocol supplementary concept word, rare disease supplementary concept word, unique identifier, synonyms] | 155 | 160 |
| 35 | (bronchial adj3 carcinogenesis).mp. [mp=title, book title, abstract, original title, name of substance word, subject heading word, floating sub-heading word, keyword heading word, organism supplementary concept word, protocol supplementary concept word, rare disease supplementary concept word, unique identifier, synonyms] | 96 | 98 |
| 36 | (bronchial adj3 adenoma*).mp. [mp=title, book title, abstract, original title, name of substance word, subject heading word, floating sub-heading word, keyword heading word, organism supplementary concept word, protocol supplementary concept word, rare disease supplementary concept word, unique identifier, synonyms] | 645 | 651 |
| 37 | (bronchogenic adj3 neoplasm*).mp. [mp=title, book title, abstract, original title, name of substance word, subject heading word, floating sub-heading word, keyword heading word, organism supplementary concept word, protocol supplementary concept word, rare disease supplementary concept word, unique identifier, synonyms] | 46 | 46 |
| 38 | (bronchogenic adj3 cancer*).mp. [mp=title, book title, abstract, original title, name of substance word, subject heading word, floating sub-heading word, keyword heading word, organism supplementary concept word, protocol supplementary concept word, rare disease supplementary concept word, unique identifier, synonyms] | 419 | 422 |
| 39 | (bronchogenic adj3 carcinoma*).mp. [mp=title, book title, abstract, original title, name of substance word, subject heading word, floating sub-heading word, keyword heading word, organism supplementary concept word, protocol supplementary concept word, rare disease supplementary concept word, unique identifier, synonyms] | 10947 | 10974 |
| 40 | (bronchogenic adj3 tumor*).mp. [mp=title, book title, abstract, original title, name of substance word, subject heading word, floating sub-heading word, keyword heading word, organism supplementary concept word, protocol supplementary concept word, rare disease supplementary concept word, unique identifier, synonyms] | 139 | 140 |
| 41 | (bronchogenic adj3 tumour*).mp. [mp=title, book title, abstract, original title, name of substance word, subject heading word, floating sub-heading word, keyword heading word, organism supplementary concept word, protocol supplementary concept word, rare disease supplementary concept word, unique identifier, synonyms] | 46 | 47 |
| 42 | (bronchogenic adj3 malignanc*).mp. [mp=title, book title, abstract, original title, name of substance word, subject heading word, floating sub-heading word, keyword heading word, organism supplementary concept word, protocol supplementary concept word, rare disease supplementary concept word, unique identifier, synonyms] | 50 | 50 |
| 43 | (bronchogenic adj3 adenocarcinoma*).mp. [mp=title, book title, abstract, original title, name of substance word, subject heading word, floating sub-heading word, keyword heading word, organism supplementary concept word, protocol supplementary concept word, rare disease supplementary concept word, unique identifier, synonyms] | 122 | 126 |
| 44 | (bronchogenic adj3 carcinogenesis).mp. [mp=title, book title, abstract, original title, name of substance word, subject heading word, floating sub-heading word, keyword heading word, organism supplementary concept word, protocol supplementary concept word, rare disease supplementary concept word, unique identifier, synonyms] | 11 | 11 |
| 45 | (bronchogenic adj3 adenoma*).mp. [mp=title, book title, abstract, original title, name of substance word, subject heading word, floating sub-heading word, keyword heading word, organism supplementary concept word, protocol supplementary concept word, rare disease supplementary concept word, unique identifier, synonyms] | 22 | 22 |
| 46 | exp Lung Neoplasms/ | 268527 | 294477 |
| 47 | 1 or 2 or 3 or 4 or 5 or 6 or 7 or 8 or 9 or 10 or 11 or 12 or 13 or 14 or 15 or 16 or 17 or 18 or 19 or 20 or 21 or 22 or 23 or 24 or 25 or 26 or 27 or 28 or 29 or 30 or 31 or 32 or 33 or 34 or 35 or 36 or 37 or 38 or 39 or 40 or 41 or 42 or 43 or 44 or 45 or 46 | 367335 | 414637 |
| 48 | exp Risk Factors/ | 944854 | 1028902 |
| 49 | risk*.mp. [mp=title, book title, abstract, original title, name of substance word, subject heading word, floating sub-heading word, keyword heading word, organism supplementary concept word, protocol supplementary concept word, rare disease supplementary concept word, unique identifier, synonyms] | 3241556 | 3792281 |
| 50 | epidemiolog*.mp. [mp=title, book title, abstract, original title, name of substance word, subject heading word, floating sub-heading word, keyword heading word, organism supplementary concept word, protocol supplementary concept word, rare disease supplementary concept word, unique identifier, synonyms] | 2318087 | 2605943 |
| 51 | incidence.mp. [mp=title, book title, abstract, original title, name of substance word, subject heading word, floating sub-heading word, keyword heading word, organism supplementary concept word, protocol supplementary concept word, rare disease supplementary concept word, unique identifier, synonyms] | 997517 | 1130356 |
| 52 | prevalence.mp. [mp=title, book title, abstract, original title, name of substance word, subject heading word, floating sub-heading word, keyword heading word, organism supplementary concept word, protocol supplementary concept word, rare disease supplementary concept word, unique identifier, synonyms] | 854385 | 989522 |
| 53 | (health adj3 correlate*).mp. [mp=title, book title, abstract, original title, name of substance word, subject heading word, floating sub-heading word, keyword heading word, organism supplementary concept word, protocol supplementary concept word, rare disease supplementary concept word, unique identifier, synonyms] | 3933 | 4737 |
| 54 | associat*.mp. [mp=title, book title, abstract, original title, name of substance word, subject heading word, floating sub-heading word, keyword heading word, organism supplementary concept word, protocol supplementary concept word, rare disease supplementary concept word, unique identifier, synonyms] | 5508821 | 6379604 |
| 55 | 48 or 49 or 50 or 51 or 52 or 53 or 54 | 8908892 | 10216571 |
| 56 | non-smok*.mp. [mp=title, book title, abstract, original title, name of substance word, subject heading word, floating sub-heading word, keyword heading word, organism supplementary concept word, protocol supplementary concept word, rare disease supplementary concept word, unique identifier, synonyms] | 24388 | 26784 |
| 57 | never-smok*.mp. [mp=title, book title, abstract, original title, name of substance word, subject heading word, floating sub-heading word, keyword heading word, organism supplementary concept word, protocol supplementary concept word, rare disease supplementary concept word, unique identifier, synonyms] | 11814 | 13223 |
| 58 | nonsmok*.mp. [mp=title, book title, abstract, original title, name of substance word, subject heading word, floating sub-heading word, keyword heading word, organism supplementary concept word, protocol supplementary concept word, rare disease supplementary concept word, unique identifier, synonyms] | 19759 | 20776 |
| 59 | "never smok*".mp. [mp=title, book title, abstract, original title, name of substance word, subject heading word, floating sub-heading word, keyword heading word, organism supplementary concept word, protocol supplementary concept word, rare disease supplementary concept word, unique identifier, synonyms] | 11814 | 13223 |
| 60 | "non smok*".mp. [mp=title, book title, abstract, original title, name of substance word, subject heading word, floating sub-heading word, keyword heading word, organism supplementary concept word, protocol supplementary concept word, rare disease supplementary concept word, unique identifier, synonyms] | 24388 | 26784 |
| 61 | "non-current smok*".mp. [mp=title, book title, abstract, original title, name of substance word, subject heading word, floating sub-heading word, keyword heading word, organism supplementary concept word, protocol supplementary concept word, rare disease supplementary concept word, unique identifier, synonyms] | 102 | 119 |
| 62 | "non current smok*".mp. [mp=title, book title, abstract, original title, name of substance word, subject heading word, floating sub-heading word, keyword heading word, organism supplementary concept word, protocol supplementary concept word, rare disease supplementary concept word, unique identifier, synonyms] | 102 | 119 |
| 63 | exp Non-Smokers/ | 670 | 801 |
| 64 | 56 or 57 or 58 or 59 or 60 or 61 or 62 or 63 | 53072 | 57643 |
| 65 | 47 and 55 and 64 | 4992 | 5581 |
| 66 | limit 65 to (english language and yr="2015")  2017 for repeat search | 231 | 216 |
| 67 | 66 not ((exp animal/ or nonhuman/) not exp human/) | 230 | 215 |
| **SCOPUS (17/1/23)** | | |  |
| 1 | TITLE-ABS-KEY ( ( lung OR pulmonary OR bronchopulmonary OR bronchial OR bronchogenic ) W/3 ( neoplasm OR cancer OR carcinoma OR tumour OR malignanc* OR adenocarcinoma OR carcinogenesis OR adenoma ) ) AND TITLE-ABS-KEY ( risk OR epidemiolog* OR incidence OR prevalence OR ( health W/3 correlate ) OR associat ) AND TITLE-ABS-KEY ( "non smok*" OR "never smok*" OR "non current smok*" ) AND PUBYEAR > 2015 AND ( LIMIT-TO ( LANGUAGE , "English" ) )  2017 for repeat search | 1375 | 1601 |

# Appendix 2: Data extraction form

**Reviewer details**

- Name of reviewer:
- Date extracted:
- Agreed for inclusion: Yes/No

**Article details**

- Year published:
- Authors:
- Title of article:
- Journal:
- Volume:
- Issue:
- Page:
- DOI:
- Study funding source:
- Conflicts of interest:

**Study details**

- Study name:
- Prospective or retrospective:
- Study design:
- Total duration of study (in years, specify dates):
- How LCINS diagnosed:
- How never-smokers (NS) with lung cancer recruited:
- Exposures studied:
- How exposures defined:
- Number of participants excluded and why:
- Number of participants who withdrew:
- Number lost-to-follow-up:
- Any other missing participants and reasons

**Participant demographics**

- Total number in study
- Total number of NS (i.e. with and without LCINS):
- Total number NS with LCINS
- Location(s) of study (and number per country):
- Age range of NS with LCINS
- How age reported e.g. mean, median, proportion
- Sex (proportion of females) of NS with LCINS
- Ethnicity of NS with LCINS (and number per group)
- Other relevant factors e.g. stage Stage of LC or other relevant details

**Risk factors reviewed**

- Risk factor reviewed:
- Number of NS in exposed group:
- Number NS with LCINS in exposed group:
- Number NS in non-exposed group:
- Number NS with LCINS in non-exposed group:
- Unadjusted HR:
- Adjusted HR:
- Confounders adjusted for:
- Link to data:

# Appendix 3: Studies reviewing association of risk factors with outcome of lung cancer in never-smokers

| **Study name** | **Study type** | **Report first author, year** | **Country /territory** | **Sample size** (never-smokers, NS) | **Sample size**  (NS with lung cancer) | **Age (mean ± SD)** | **Female** | **Ethnicity (%)** | **Recruit-ment and period of follow-up** | **How NS defined** | **Factors studied** | **Confounders adjusted for** |
| --- | --- | --- | --- | --- | --- | --- | --- | --- | --- | --- | --- | --- |
| 45 and Up | Cohort | Cheng E.S.  2022 | Australia | 132,354 | 226 | 72.4 (11.0) | 61.5% | Asian 20 (8.8%)  Non-Asian 206  (91.2%) | 2006 - 2008  Median follow-up 5.41 years | Questionnaire | Age birth of first child  Alcohol intake Anti-hypertensives Asthma  BMI  COPD  Country of birth  Diabetes  Family history  Fruit intake  Height  Hormonal contraceptive  Hormone replacement therapy  Menopausal age  Parity  Passive smoking  Physical activity  Processed meat  Red meat intake  Sex | Age, sex, socioeconomic status, region of residence, education, height, Asian country of birth, family history of lung cancer, passive smoking, COPD, physical activity, BMI, alcohol, fruit intake, vegetable intake, red meat intake, parity, age at birth of first child, hormonal contraceptive use, HRT, menopausal age |
| Adventist Health Study 2 (AHS-2) | Cohort | Fraser G.E.  2020 | USA | 64,700 | 117 | 74.8 | 73.5% | American Indian 1 (0.9%)  Asian 3 (2.5%)  Black 27 (23.1%)  Hispanic 2  (1.7%)  White 84 (71.8%) | Average follow-up 7.8 years | Questionnaire | Adventists, who often follow vegetarian diets | Race, sex, smoking, education, region of residence. |
| Agricultural Health Study (AHS) | Cohort | Bonner M.  2017 | USA | 26,859 | 57 | 58.9  (11.4) | 3.5% | Black/Other: 1 (1.75%)  White: 55 (96.5%) | Average follow-up 14.8 years | Questionnaire | Pesticide | Age, sex, total days pesticide |
| Adventist Health and Smog Study-2 (AHSMOG-2) | Cohort | Gharibvand L.  2017 | USA | 64,906 | 115 | Unknown | Unknown |  | 2002 - 2011  Median follow-up 7.5 years | Questionnaire | PM2.5 | Sex, race, education level |
| Asbestos Related Diseases Cohort (ARDCO) | Cohort | Gallet J.  2022 | France | 1,305 | 14 | 71.2 (4.9) | 0% | Not available | 2003 - 2005  Up to 13 years follow-up | Questionnaire | Pleural plaques | Exposure to asbestos, time since first exposure, time since last exposure, smoking status |
| Atherosclerosis Risk in Communities Study (ARIC) | Cohort | Michaud D.S.  2018 | USA | 2,847 | ~13-19 | Unknown | Unknown |  | 1987 - 1989  Follow-up 10 years | Study visits | Periodontitis and edentulism | Age, race, field centre, education, smoking, alcohol intake, BMI, diabetes,  sex, HRT |
|  |  | Nohara S.  2025 |  | 5,548 | 1-12 |  |  |  | Median follow-up 25.3 years |  | Peripheral arterial disease | Age, sex, race, field centre, education |
| Black Women’s Health Study (BWHS) | Cohort | Erhunmwunsee L.  2022 | USA | 37,650 | 77 | 59.4 (13.2) | 100% | Black 100% | 1995 - 2018  Median follow-up 22.6 years | Questionnaire | Annual family income  BMI  Educational attainment  Health insurance  Neighbourhood deprivation  PM2.5  Secondhand smoke | Age, BMI, health insurance status, education, income, second hand smoke at home, PM2.5 |
|  |  | Nomura S.J.O.  2018 |  | 31,134 | 45 | Unknown |  | Black 100% | 1995 - 2013  Mean follow-up 16.9 years |  | Fruit and vegetable intake | Age, caloric intake, education, geographic region, physical activity, sedentary time, BMI |
|  |  | Bethea, T.N.  2022 |  | Unknown | Unknown | Unknown |  | Black 100% | 1995 - 2017 |  | Physical activity and inactivity | Age, time, marital status, education, BMI, menopausal status, alcohol intake, pack-years smoking, fruit and vegetable consumption, geographic region |
| Canadian Partnership for Tomorrow’s Health (CanPath) | Nested case–control study | Murphy R.A.  2022 | Canada | 950 | 190 | 57.8 (8.8) | 60.5% | Non-white 61  (32.1%)  White 128 (67.4%) | Minimum 7 years follow-up | Questionnaire | Alcohol consumption BMI  Fruits and vegetables Physical activity  Sleep duration  Waist circumference  Waist to hip ratio | Age, sex, regional cohort, income |
| Cancer Screening Program in Urban China (CanSPUC)/China National Lung Cancer Screening (NLCS) | Cohort | Wang F.  2021 | China | 547,218 | 1,620 | 59.9 (7.5) | 100% | Not available | 2013 - 2019  Median follow-up 3.9 years | Questionnaire | Family history of lung cancer | Age, BMI, passive smoking, education, occupational exposure to hazardous substances, respiratory diseases, vegetable intake, fruit intake, alcohol, tea drinking, psychological trauma |
|  |  | Guo L.  2023 |  | 151,834 | 204 | 60.2 (7.6) |  | 98.5% Han Chinese | 2013 – 2019  Follow-up until March 2020 |  | Chronic respiratory disease  Emphysema  Tuberculosis  Marital status | For chronic respiratory disease only: includes age, family history, menopause, benign breast disease |
|  |  | Wu Z.  2022 |  | 794,283 | 3,351 | Unknown | 62.8% |  | Median follow-up 4.80 years |  | Air pollution  Alcohol consumption  Cooking oil fumes  Diabetes  Education  Family history of cancer  Gastrointestinal disease  Hepatobiliary disease  Hyperlipidemia  Hypertension  Occupational exposure  Passive smoking | Age, separate HR for sex |
|  |  | Yang Z.  2021 |  | 529,823 | 1,454 | Unknown | 100% |  | 2013 - 2018  Median follow-up 3.61 years |  | Age at menarche, first live birth and menopause  Benign breast disease Duration of breastfeeding  Gynaecological surgery  Menopause status | Age at menarche, age at first live birth, duration breastfeeding, benign breast disease, surgery of reproductive system, age, education, BMI, smoking status, environmental tobacco smoke, cooking smoke, occupational exposure, history of COPD, family history lung cancer |
| China H-type Hypertension Registry Study (CHHRS) | Nested case-control | Wei Y.  2022 | China | 555 | 261 | Unknown | Unknown |  | Follow-up 3 years | Questionnaire | 5-methyltetrahydrofolate  Folate | Age, sex, centre, education, alcohol intake, hypertension, BMI, pulse, cholesterol, triglycerides, fasting glucose, total homocysteine, vitamin B12 |
| China Kadoorie Biobank (CKB) | Cohort | Li X.  2019 | China | 334,082 | 1,988 | 58.9 (9.7) | 83.4% | Chinese 100% | 2004 - 2008  Follow-up 10.1 years | Questionnaire | Tea consumption | Age, sex, education, occupation, marital status, household income, physical activity,  intakes of red meat, fresh fruit, vegetable, preserved vegetable, diabetes, heart disease, stroke, peptic ulcer, chronic hepatitis or cirrhosis, gallstone or gallbladder disease, menopausal status, age at first period, oral contraceptives, number of live births, secondhand smoke, family history of cancer |
|  |  | Jian S.  2022 |  | 32,186 | Unknown | Unknown | Unknown |  |  |  | Physical activity | Age, sex, education, marital status, alcohol intake, smoking status, consumption of fresh fruit and red meat, BMI |
|  |  | Zhu M.  2024 |  | 255,829 | 2,571 |  |  |  | Median follow-up 12.17 years |  | PM2.5 | Age, sex, education, income, living area, red meat intake, fruit, passive smoking, household air pollution, self-rated health, BMI |
|  |  | Ma Z.  2023 |  | 336,526 | 2,428 |  |  |  | Median follow-up 10.1 years |  | Residential area  Height  BMI  COPD  Previous cancer | Age, residential area, height, BMI, physical activity, cough, emphysema/bronchitis, previous cancer, family history cancer |
|  |  | Weng C.  2024 |  | 10,678 | 130 |  |  |  | Median follow-up 12.2 years |  | Mosaic loss of chromosome Y | Age, region, ethnic background, income, education, occupation, smoking, alcohol, MET, diet, BMI, diabetes, hypertension |
| Cancer Prevention Study (CPS) Nutrition Cohort | Cohort | Patel A.V.  2017 | USA | Unknown | Unknown | Unknown | Unknown |  | From 1992  Follow-up until 2011 | Questionnaire | BMI  MET  Waist circumference | Age, sex, race, sitting time, marital status, prevalent disease (emphysema or other lung diseases), education, cigarettes per day and smoking duration, fruits and vegetable intake, BMI in MET-hours/week models, MET-hours/week in BMI models, and PA and BMI in waist |
|  | Nested case-control | Tang Z.  2024 |  | 462 | 114 |  |  |  | 1992-1993, 2006-2013 |  | Sphingomyelin Taurodeoxycholic acid 3-sulfate | Age, sex, race, date of blood draw, BMI, hours since last meal, physical activity, fruits, and vegetables consumption, hormone use |
| European Prospective Investigation into Cancer and Nutrition (EPIC) | Cohort | Heath A.K.  2022 | Denmark  France  Germany  Greece  Italy  Netherlands  Norway  Spain  Sweden  UK | 194,087 | 278 | 56.0 (8.2) | 76.0% | White 100% | 1992 – 2000  Median follow-up 15 years | Questionnaire | Food intake: beer/cider, fibre, fruit, offal, retinol, vitamin C | Age, study centre, sex, smoking status, cigarettes, cigarette smoking years, BMI, physical activity education, diabetes, energy intake |
| Golestan Cohort Study (GCS) | Cohort | Wang Q.  2021 | Iran | Unknown | 62 | 59.6 (9.3) | 48.4% | Turkman 75.8% | 2004 - 2008  Median follow-up 12 years | Questionnaire | Dietary indices | Age, gender, race/ethnicity, BMI, education, residence, socioeconomic score, marital status, opium, alcohol intake, total energy intake, physical activity |
|  |  | Yano Y.  2024 |  | 41,366 | 53 |  |  |  | Median follow-up 14 years |  | Sex  Deprivation  Rural  Education  Dental health | Age, sex, socioeconomic status, ethnicity, residence, education, nass use, dental hygiene |
| Health in Men Study (HIMS) | Cohort | Chan Y.X.  2017 | Australia | 1,226 | 3 | 75.8 (2.8) | 0% | Not available | 1996 - 1999,  Median follow-up 9.2 years | Questionnaire | Testosterone | Age, BMI, smoking status, vigorous physical activity, alcohol intake, diabetes, high density lipoprotein, triglycerides, previous diagnosis of cancer |
| Japan Collaborative Cohort Study for Evaluation of Cancer Risk (JACC) | Cohort | Fangyu Y.  2022 | Japan | Unknown | 126 | Unknown | Unknown |  | 1988 – 1990  Median follow-up 14.6 years | Questionnaire | Dietary vitamin K intake | Age, sex, residence, alcohol intake, BMI, family history of cancer, mental stress, smoking status, sports hours, walking hours, education, energy, vitamin A, vitamin C, β-cryptoxanthin intake |
| Japan Public Health Center (JPHC) | Cohort | Mori N.  2017 | Japan | 54,659 | 509 | 60.7 (7.3) | 70.3% | Japanese 100% | 1990 - 1993  Median follow-up 14.9 years | Questionnaire | Cruciferous vegetable intake | Age and area adjusted |
|  |  | Abe S.K.  2021 |  | 54,950 | 600 | 56.7 (7.6) | 80.2% |  | 1990 - 1994  Average follow-up 19.1 years |  | BMI  Height  Weight change | Age, area, alcohol intake, physical activity intake of fruit vegetables and isoflavones, BMI |
|  |  | Wilunda C.  2021 |  | 42,615 | 400 | 51.5 | 100% |  | 1990 - 1994  Median follow-up  21 years |  | Reproductive factors | Age, area, residence, BMI, alcohol intake, passive smoking at home or at the workplace, sports activity, family history of lung cancer, asthma |
| Kailuan Cohort Study | Cohort | Lyu Z.  2019 | China | 56,097 | 438 | Unknown | 0% |  | From 2006  Median follow-up 8.83 years | Questionnaire | Blood lipids | Age, education, income, smoking, pack-years, alcohol intake, coal dust exposure and degree, FBG physical activity, dietary fat intake, BMI |
|  |  | Wu Z.  2022 |  | 37,085 | 224 | 58.6 (9.6) | 0% |  | Median follow-up 5.16 years |  | Annual BMI change | Age, income, education, alcohol intake, coal dust exposure and degree, physical activity, dietary fat intake, TC, TG, LDL-C, waist circumference, BMI |
| Kangbuk Samsung Cohort Study | Cohort | Lee Y-G  2025 | South Korea | 189,439 | 173 | 50.5 | 69.9% | 100% Korean | 2011-2020  8,309,600 person-years | Health examination and questionnaires | Family history of lung cancer | Age, sex, BMI, alcohol intake, vigorous exercise, diabetes, hypertension, dyslipidaemia |
| Korean Cancer Prevention Study-II (KCPS-II) | Cohort | Shin J.W.  2025 | South Korea | 63,880 | 721 | Unknown | Unknown | Unknown | 2004 – 2013  Median follow-up 14 years | Questionnaires and health examination | Bilirubin levels | Age, sex, alcohol, BMI, liver blood tests (GOT and GGT) |
| Lianyungang | Nested case-control | Ruan G-T  2023 | China | 182 | 88 | Unknown | Unknown | Unknown | 2016 – 2018  Median follow-up 1.8 years | Questionnaire | Sleep quality | Sleep duration, siesta habit, BMI, stress intensity, alcohol, smoking, economic conditions, education, labour intensity, snoring |
| Life Span Study | Cohort | Cahoon E.K.  2017 | Japan | Unknown | 432 | Unknown | 90.7% |  | 1958 - 2009 | Questionnaire | Radiation dose | Age, sex, age at exposure |
| Lifelines | Cohort | Du Y.  2022 | Netherlands | 45,430 | 43 | 54.5 (12.2) | 67.4% | Not available | 2006 – 2013  Median follow-up  9.5 years | Questionnaire | Airflow limitation | Age, sex, education, smoking, passive smoking, asthma, asbestos exposure |
| Mass General Brigham Biobank | Nested case-control | Tian R.  2022 | USA | 548 | 94 | Unknown | Unknown | Unknown | 2010 – 2021  Median follow-up 5.1 years | Linked electronic health records and survey data | Clonal haematopoiesis | Age and year at blood draw, sex, race, family history of lung cancer, principal components of genetic ancestry |
| Miyagi Cohort Study | Cohort | Mugikura M.  2020 | Japan | 18,907 | 233 | Unknown | 81.1% |  | 1990 - 2014  Mean follow-up 24.5 years | Questionnaire | Height | Age, weight family history of cancer, education, alcohol intake, time spent walking vegetable intake, fruit intake |
| MJ Health Database | Cohort | Huang H-L  2021 | Taiwan | 130,559 | 468 | 53.3 (12.1) | 71.2% | Unknown, likely all Taiwanese | 2000 – 2015  Median follow-up 12.3 years | Self-reported questionnaire | Sex  Education  BMI  eGFR  CEA  Family history lung cancer  Fruit/vegetable  PM2.5  Hypertension Diabetes Stroke Cardiovascular disease | Age, gender, education, BMI, eGFR, CEA, family history, hypertension, diabetes, stroke, cardiovascular disease, fruit intake, pollution |
| Multiethnic Cohort Study (MEC) | Cohort | Park S.Y.  2021 | USA | 80,635 | 635 | 63.4 (8.3) | 71.8% | AAPI 281 (44.0%)  African American 96 (15%)  Latino 142 (22.0%)  White 116 (18.0%) | 1993 -1996  Average follow-up 17.5 years | Questionnaire | Dietary indices | Age, sex, ethnicity, family history lung cancer, education, BMI, physical activity, total energy intake, alcohol intake. |
|  |  | Choi E.  2023 |  | 94,579 | 849 | Unknown | 78.9% |  | 1993 - 1996  Follow-up up to 20 years |  | Primary lung cancer |  |
| National Health Insurance Service (NHIS) | Cohort | Cho M.H  2023 | South Korea | 247,688 | 1,082 | Unknown | Unknown | Not available, likely predominantly Korean | 2010 – 2017  Mean follow-up 4.5 years | Questionnaire | Rheumatoid arthritis | Age, sex, smoking, alcohol intake, regular physical activity, income level, BMI. diabetes, hypertension, dyslipidaemia, CKD, MI, stroke, depression, ILD |
|  |  | Park H.Y.  2020 |  | 241,633 | 905 | Unknown | Unknown |  | 2002 - 2013  Median follow-up 7.0 years |  | COPD | Age as time scale, sex, BMI, Charlson comorbidity index |
|  |  | Park H.Y.  2022 |  | 32,086 | 226 | Unknown | Unknown |  | 2003 – 2015  Median follow-up 7.7 years |  | Pulmonary tuberculosis | Age as time scale, sex, BMI, smoking status, Charlson Comorbidity Index, income |
|  |  | Choi H.  2022 |  | 2,323,605 | 10,400 | Unknown | Unknown |  | 2009  Minimum follow-up 9 years |  | Bronchiectasis | Age, sex, BMI, smoking, alcohol intake, income, physical activity, Charlson Comorbidity Index |
|  |  | Moon S.  2020 |  | 39,784 | 165 | Unknown | Unknown |  | 2003 -2015,  Median follow-up 7.8 years |  | Anti-hypertensive medication | Age, gender, income, smoking, alcohol, physical activity, other antihypertensives, diabetes, dyslipidaemia, myocardial infarction, congestive heart failure, cerebrovascular disease, chronic kidney disease, COPD, Charlson Comorbidity, BMI, blood pressure, blood sugar, cholesterol, liver blood tests, number of physician visits |
|  |  | Kang J.  2021 |  | 515,100 | 2,753 | Unknown | Unknown |  | 2022  Follow-up 10 years |  | Aspirin  Metformin  Statins | Age, sex, income, BMI, smoking, alcohol intake |
|  |  | Choi Y.J.  2019 |  | Unknown | Unknown | Unknown | Unknown |  | 2009 – 2012  Minimum follow-up 3 years |  | Height | Age, sex, BMI, alcohol intake, physical activity, diabetes |
|  |  | Jeon K.H  2020 |  | 4,335,259 | 15,223 | 61.4 (8.7) | 100% |  | Median follow-up 4.4 years |  | Alcohol consumption  Body mass index Dyslipidaemia  Hypertension  Income Regular physical activity Reproductive factors | Age, age at menarche and menopause, parity, breastfeeding, of HRT, oral contraceptive, alcohol intake, smoking, exercise, income, BMI, hypertension, diabetes, dyslipidaemia, cancer |
|  |  | Park H.J.  2019 |  | 300,518 | 1,972 | 63.4 (9.6) | 48.4% |  | 2003 - 2004.  Follow-up 8 years |  | Fasting blood sugar  T2DM | Age, sex, income, alcohol, physical activity, Charlson Comorbidity Index, BMI, diabetes, blood pressure, cholesterol |
|  |  | Yang S.  2021 |  | 55,954 | 285 | Unknown | Unknown |  | 2005 – 2007    Follow-up 9 years |  | Ambient air pollution | Age, sex, health insurance, employment, smoking, alcohol intake, physical activity, diet, BMI, obesity, hypertension, diabetes, hyperlipidaemia, family history of diseases (cancer, hypertension, and diabetes), medical use |
|  |  | Lee H.W.  2022 |  | 3,553,833 | 18,861 | 53.4 (8.6) | 58.8% |  | 2005 – 2007  Follow-up until 2015 |  | PM_10_ level | Age, BMI, income, previous cancer, chest X-ray abnormality |
|  |  | Ko Y.H  2020 |  | 5,860,922 | 43,473 | 61.1 (11.4) | 100% |  | 2003 -2004  Median follow-up 11.4 years |  | Cancer history  Meat intake  Urban residence | Age, BMI, exercise, alcohol intake, diet, residence, previous cancer history |
|  |  | Kim B-G  2024 |  | 90,947 |  |  |  |  | 2002 – 2019    Median follow-up 5.1 years |  | Chronic periodontitis | Matched by age and sex, adjusted for BMI, smoking status |
| Netherlands Cohort Study (NLCS) | Case-cohort | Heath A.K.  2022 | Netherlands | 1,556 | 198 | 62.8 (4.3) | 52.0% | White 100% | 1986  Follow-up up to 20.3 years | Questionnaire | Food intake: beer/cider, fibre, fruit, offal, retinol, vitamin C | Age, sex, smoking status, BMI, physical activity, education, family history of lung cancer, diabetes, energy intake |
|  |  | Schulpen M.  2018 |  |  |  |  |  |  |  |  | Mediterranean diet | Age, gender, smoking status,  cigarette smoking, daily energy intake, alcohol intake, BMI,  non-occupational physical activity, education, family history of lung cancer,  chronic bronchitis |
| NIH-AARP Diet and Health Study | Cohort | Korn A.R.  2022 | USA | 86,242 | 271 | 71.6 (5.1) | 57.6% | White 256 (95%)  Black 6 (2%)  Other 9 (3%) | 1995 –1996  Mean follow-up 7.0 years | Questionnaires | 2018 World Cancer Research Fund/American Institute for Cancer Research Score calculated from weight, physical activity, diet, alcohol intake | Age, race/ethnicity, marital status, education, energy intake, hormone replacement therapy, diabetes |
| Nurses Health Study (NHS) | Cohort | Huang T.  2021 | USA | 31,323 | 74 | 77.3 (6.5) | 100% | White 69 (93.0%) | 1976 - 2008  Follow-up 8 years | Questionnaire | Obstructive sleep apnoea | Age, ethnicity, BMI, height, family history of cancer, physical activity, alcohol intake, sleep duration, duration of HT by type, diabetes, regular physical examination |
|  |  | Trudel-Fitzgerald C.  2022 |  | Unknown | 129 | 60.6 (6.7) |  | Asian 1 (0.8%)  Black 2 (1.6%)  Multi-racial 1 (0.8%)  White 120 (93.0%) | 1992  Follow-up 24 years |  | Depressive symptoms | Age, exposure to second-hand smoke in childhood, parents' occupations, family history of lung cancer, husband's education |
| Norwegian Women and Cancer Study (NOWAC) | Nested cohort | Sandanger T.M.  2018 | Norway | 71 | 14 | 57.4 | 100% | Not available | 1991 - 2006  Median follow-up 4.7 years | Questionnaire | DNA methylation | Age, sample storage time, technical covariates, blood cell composition |
|  | Cohort | Hansen M.S.  2021 |  | 43,713 | 96 | Unknown |  |  | 1991 - 2007  Mean follow-up 15.9 years |  | Passive smoking | Age, education, alcohol intake |
|  |  | Borch K.B.  2019 |  | Unknown | 61 |  |  |  | 1996 – 2004  Follow-up 6-8 years later |  | Physical activity | BMI, education, smoking, fruit intake, birth cohort |
| Prostate, Lung, Colorectal and Ovarian (PLCO) Cancer Screening Trial | Cohort | Wang Q.  2021 | USA | 48,549 | 141 | 65.2 (5.1) | 62.4% | Hispanic 1.42%  Non-Hispanic black 1.42%  Non-Hispanic white 92.2% Other 4.96% | 1993 - 2001  Median follow-up 12.2 years | Questionnaire | Isoflavones  Phytoestrogen levels | Age, gender, race, BMI, education, alcohol, marital status, family history of lung cancer, total daily energy intake, total daily β-carotene intake, daily vitamin C, daily vitamin E intake |
|  |  | Abdel-Rahman O.  2020 |  | 49,569 | 136 | Unknown | 64.7% |  | 1992 – 2009 |  | Family history of lung cancer  Race  Secondhand smoke  Sex | Age, sex, family history of lung cancer, study arm |
|  |  | Titan A.L.  2020 |  | 42,059 | Unknown | Unknown | 100% |  |  |  | Hormone replacement therapy | Age, BMI, race, education, marital status, family history of cancer and lung cancer, emphysema, bronchitis, pregnancy, birth control, stroke, cardiac disease |
|  |  | Zhu Z.  2023 |  | 24,588 | 61 | Unknown | Unknown |  | 1993 – 2001  Mean follow-up 8.84 years |  | DASH score | Age, gender, race, alcohol, BMI, randomization group, hypertension, family history of lung cancer, energy intake |
|  |  | Gai X.  2024 |  | 38,883 | 111 | Unknown | 100% |  | 1993 – 2001  Median follow-up 11.3 years |  | Menopause age | Age, intervention arm, race, education, BMI, menopause hormone therapy use |
| Quzhou Environmental Exposure and Human Health (QEEHH) | Nested case-control | Mao W.  2024 | China | 228 | 110 | Unknown | Unknown | Unknown | 2019 – 2020  Follow-up until Nov 2022 | Face-to-face interview | Urinary benzothiazole, benzotriazole and derivatives | Age, sex, BMI, marital status, education, household income, occupation, residence, dietary habit, smoking status, alcohol |
| Shanghai Men’s Health Study (SMHS) | Nested case-control | Yuan J.  2018 | China | 232 | 116 | 58.6 (5.3) | 0% | Chinese 100% | 1986 - 2013  Mean follow-up 11.3 years | In-person interviews | Oxidative damage biomarker 8-epi-prostaglandin F2α | Age, year of biospecimen collection, residence, education, BMI |
|  |  | Hosgood H.D.  2021 |  | 48 | 24 | 64.2 (7.6) |  |  | Follow-up 7.2 years cases,  13.9 years controls |  | Oral microbiome | Age, sex, sample collection, education, antibiotic use |
|  |  | Wong J.Y.Y.  2024 |  | 323 | 161 | 62.3 (8.4) |  |  |  |  | Leukocyte telomere length | Age, BMI, education, alcohol intake |
|  | Cohort | Sun J.  2018 |  | Unknown | 94 | Unknown |  |  | Mean follow-up 14.8 years |  | Carbohydrate intake | Age, education, income, BMI, physical activity, total energy intake, alcohol, lung disease, hypertension, diabetes, family history of cancer |
| Shanghai Women’s Health Study (SWHS) | Nested case- control | Hosgood H.D.  2021 | China | 180 | 90 | 58.0 (8.8) | 100% | Chinese 100% |  | In-person interviews | Oral microbiome | Age, sex, sample collection time, menopause status, education, antibiotic use |
|  | Nested case- control | Shiels M.S.  2017 |  | 511 | 248 | 57.84 (estimate) |  |  | 1996 - 2000  Mean follow-up 7.5 years |  | Inflammatory cytokines  CRP | Age, date of blood collection, exposure to passive smoke, poor indoor ventilation, chronic bronchitis |
|  | Cohort | Sun J.  2018 |  | Unknown | 594 | Unknown |  |  | Mean follow-up 14.8 years |  | Carbohydrate intake | Age, education, income, BMI, physical activity, total energy intake, lung disease, hypertension, diabetes, family history of cancer, menopausal status |
|  | Nested case- control | Seow W.J.  2019 |  | 564 | 275 | 61.0 |  |  | 1996 - 2000  Median follow-up  10.9 years |  | Urinary metabolites | Age, BMI, respiratory diseases, secondhand smoke exposure |
|  | Cohort | Hung N.L.  2018 |  | 65,076 | 714 | 57.2 (8.9) |  |  | 1996 – 2000  Mean follow-up 8.3 years cases, 13.9 years controls |  | Dietary polyunsaturated fatty acids | Age, smoking status, alcohol intake, BMI, physical activity, vitamin supplement use, menopausal status, HRT |
|  | Nested case-control | Zhao Y.  2022 |  | 794 | 397 | 62.0 (5.6) |  |  | Average follow-up 15.6 years |  | Endogenous sex hormones and aromatase | Age, assay batch, education, occupation, passive smoking, alcohol intake, physical activity, HRT usage, family history of lung cancer |
|  | Nested case-control | Li M.  2022 |  | 956 | 478 | 61.4 (6.1) |  |  | Follow-up 15.6 years |  | Dietary and urinary phytoestrogens | Age, education, alcohol intake, physical activity, BMI, menopausal status, family history of lung cancer s, total calories, fruits, red meat, vegetables |
|  | Nested case-control | Wong J.Y.Y.  2024 |  | 1590 | 798 | 56.8 (8.9) |  |  |  |  | Leukocyte telomere length | Age, BMI, education, alcohol intake, secondhand smoke |
| Information System for Research in Primary Care (SIDIAP) | Cohort | Recalde M.  2021 | Spain | 2,339,363 | Unknown | Unknown | Unknown |  | 2006 - 2018  Median follow-up 8.3 years | Electronic health records | BMI | Adjusted for age, sex, smoking status, alcohol intake, nationality, deprivation, type 2 diabetes |
|  | Case-control | Turkiewicz A.  2022 |  | 405,566 | Unknown | 76.2 (8.2) | 48% |  | 2009 - 2017  Follow-up 8 years |  | Knee osteoarthritis | Age, sex, BMI, smoking, alcohol intake, deprivation, hypertension, ischaemic heart disease, heart failure, cerebrovascular disease, diabetes type I or II |
| Singapore Chinese Health Study | Cohort | Yuan J.  2018 | Singapore | 18,034 | 198 | Unknown | Unknown | Chinese 100% | 1993 - 1998  Mean follow-up 11.8 years | In-person interviews | Leukocyte telomere length | Age, sex, dialect group, education, BMI, cigarettes, smoking, alcohol intake |
| Sister Study | Cohort | Mehta S.S.  2023 | USA | 28,290 | 58 | Unknown | 100% | Unknown | 2003 – 2009  Mean follow-up 11.3 years | Questionnaires | Wood-burning | Age, race/ethnicity, education, urban status, marital status, income, secondhand smoke |
| Southern Community Cohort Study (SCCS) | Cohort | Munro H.M.  2023 | USA | 25,860 | 89 | 64.26 (10.0) | 69.6% | White 17 (19.10%)  Black 69 (77.53%)  Asian or Pacific Islander <5  Mixed race (excluding Hispanic/ Latino) <5 | 2002 – 2009  Follow-up 10.6 years | Structured in-person interviews or questionnaire | HEI-10 | Sex, race, enrolment source, education, income, marital status, health insurance, BMI, diabetes, heart attack, stroke, hypertension, hypercholesterolemia, COPD, HIV/AIDS, total physical activity, hours spent sitting, energy intake, menopausal status, hormone replacement therapy |
| The Health Improvement Network (THIN) | Cohort | Rait G.  2020 | UK | 3,679,831 | 3,212 |  | 58.5% | Not available | 1998 – 2018  Median follow-up 6 years | Electronic health records | Deprivation  Sex  Urban-rural | Age |
| Utrecht Cardiovascular Cohort - Second Manifestations of ARTerial disease (UCC-SMART) | Cohort | Van't Klooster C.C.  2019 | Netherlands | 1,587 | 9 | 66.8 (6.8) | 11.1% | White 100% | 1996 - 2017  Median follow-up 8.3 years | Questionnaire | CRP | Age, sex, BMI, LDL cholesterol, diabetes mellitus, systolic blood pressure, kidney function |
| UK Biobank | Cohort | He H.  2022 | United Kingdom | 250,043 | 287 | Unknown | Unknown |  | Median follow-up 8.9 years | Questionnaire | In utero tobacco exposure | Age, sex, birthplace, body sizes at age 10 years, family history of cancer |
|  |  | Warkentin M.T.  2019 |  | 222,274 | 168 | 60.7 (6.5) | 70.2% | Black 2 (1.2%) Other/mixed 2 (1.2%)  Southeast Asian 3 (1.8%)  White 161 (95.8%) | 2006 - 2010  Median follow-up 5.6 years |  | Family history of lung cancer  FEV1/FVC  Personal history of cancer  Sex | Age, sex, ethnicity, height, education, household income |
|  |  | Wong J.Y.Y.  2021 |  | 232,528 | 205 | 61.0 (6.5) | 61.2% | Asian (South and East) 1 (0.5%)  Black 5 (2.4%)  Mixed 1 (0.5%)  Other 1 (0.5%)  White 190 (92.7%) | 2006 - 2010  Average follow-up 7 years |  | Neutrophil-to-lymphocyte ratio  White cell count | Age, BMI, ethnicity, assessment centre, alcohol intake, deprivation |
|  |  | Horsfall L.J.  2020 |  | 194,558 | 261 | Unknown | Unknown | White 100% | 2006 - 2016 |  | Serum bilirubin  Related SNP | Age, gender, year, ethnicity, height, weight, recruitment centre, smoking status |
|  |  | Christakoudi S.  2023 |  | 222,893 | 416 | 61.6 (6.1) | 65.9% | White 100% | 2006 – 2010  Mean follow-up 10.4 years |  | Platelet parameters | Age, region, menopausal status hormone replacement therapy BMI, height, recent weight change, smoking status and intensity, alcohol intake, physical activity, deprivation, family history of cancer, time of blood collection, fasting time, diabetes, lipid-lowering drugs, antihypertensive drugs, anticoagulants, non-steroidal anti-inflammatories, paracetamol |
|  |  | Huang Y.  2021 |  | 228,851- 246,664 | Unknown | Unknown | Unknown | Unknown | Median follow-up 7.1 years |  | Pollution | Age, sex, BMI, household income, education |
|  |  | Zhang J.  2023 |  | 223,682 | 251 |  |  |  | Median follow-up 7.1 years |  | Psychological distress | Age, sex, ethnicity, education, deprivation, family history of lung cancer, smoking healthy diet score, BMI, physical activity |
|  |  | Han D.  2023 |  | 234,302 | 267 |  |  |  | Median follow-up 13 years | Questionnaire | Leukocyte telomere length | Age, sex, smoking status, ethnicity, BMI, alcohol, white blood cell count, education, household income, deprivation |
|  |  | Zhang S.  2024 |  | 250,017 | 237 |  |  |  | Mean follow-up 8.79 years |  | Cardiovascular disease | Age, sex, race, family history of cancer, BMI, alcohol intake, physical activity, deprivation, menopause, aspirin, diabetes, hypertension |
|  |  | Weng C.  2024 |  | 106,714 | 302 |  |  |  | Median follow-up 12.5 years |  | Mosaic loss of chromosome Y | Age, assessment centre, ethnic background, deprivation, education, occupation, smoking, alcohol, MET, diet, BMI, diabetes, hypertension |
|  |  | Giratallah H.  2023 |  | 214,592 | 496 |  |  |  | End of follow-up 2020 |  | CYPA26 activity | Age, sex, smoking, genetic principal components |
|  |  | Guan Y.  2024 |  | 188,742 | 336 |  |  |  | Median follow-up 7 years |  | Allostatic load | Age, gender, race, family history, employment, education, income, deprivation, smoking, alcohol, physical activity, sleep quality |
|  |  | Liao Y.  2024 |  | 275,811 | Unknown |  |  |  | Mean follow-up 11.5 years |  | Gastro-oesophageal reflux disease | Age, sex, race, BMI, alcohol, family history of cancer, physical activity, deprivation, diabetes, hypertension, COPD |
|  |  | Fan Y.  2024 |  | 106,872 | Unknown |  |  |  | Median follow-up 13.2 years |  | MINDDiet | Age, sex, education, income, deprivation, family history of cancer, dietary energy intake, alcohol, physical activity, sleep duration, BMI, waist-hip ratio, blood pressure |
|  | Nested case-control | Tian R.  2022 |  | 725 | 121 |  |  |  | Median follow-up 6.0 years |  | Clonal haematopoeisis | Age and year at blood draw, sex, race, family history of lung cancer, principal components of genetic ancestry |
| Veterans Health Administration | Cohort | Brooks R.T.  2024 | USA | 31,772 | Unknown | Unknown | Unknown | Unknown | 2000-2019  Mean follow-up 6.3 years | Electronic health record with substantial agreement with survey data | Rheumatoid arthritis | Age, gender, enrolment year, race, ethnicity, Agent Orange exposure, Rheumatic Disease Comorbidity Index score |
| Women's Health Initiative (WHI) | Cohort | Myneni A.A.  2021 | USA | 41,950 | 251 | Unknown | 100% |  | 1993 - 1998  Follow-up 17 years | Questionnaire | Dietary indices | Age, pack years, exposure to secondhand smoke during childhood and adult (living and working) race, education, BMI, physical activity, energy intake |
|  |  | Gowda S.N.  2019 |  | 65,419 | 265 | 65.21 (estimated) |  | American Indian 2 (0.8%)  Asian/Pacific Islander 7 (2.65%)  Black 20 (7.5%)  Hispanic 5 (1.9%)  White 228 (10.6%) | Mean follow-up 14.1 years |  | Pollution | Age, race, BMI, region, emphysema, asthma, deprivation |
|  |  | Tao M.H.  2018 |  | 75,962 | 395 | 65.26 (estimated) |  | American Indian 3 (0.8%)  Asian/Pacific islander 13 (3.3%)  Black 24 (6.1%)  Hispanic/Latino 8 (2.0%)  Non-Hispanic white 340 (86.5%) | Mean follow-up 13.3 years |  | Oral bisphosphonate use | Age, ethnicity, education, alcohol intake, BMI, physical activity, total calcium intake, total vitamin D intake, statins, hormone treatment status |
|  |  | Nwizu N.N.  2017 |  | 34,097 | 150 | 70.8 (7.2) |  | American Indian or Alaskan Native 1 (0.7%)  Asian or Pacific Islander 7 (4.7%)  Black 8 (5.3%)  Hispanic/Latino 4 (2.7%)  Non-Hispanic White 127 (84.7%) | Mean follow-up 8.32 years |  | Periodontal disease | Age, BMI |
|  | Nested case-control | Cheng T.-Y.D.  2017 |  | 596 | 298 | 65.5 (7.1) |  |  |  |  | Vitamin D | Race/ethnicity, BMI, Trial allocation, serum retinol, season of blood draw |
|  | Randomised controlled trial | Tao M.H.  2017 |  | 18,753 | 56 | 63.9 (7.0) |  | Asian/ Pacific islander 1 (1.8%)  Black 6 (10.7%)  Hispanic/Latino  1 (1.8%)  Non-Hispanic white  48 (85.7%) | Mean follow-up 11.1 years |  | Calcium/vitamin D supplementation | Age, randomisationation arm, trial phase |
| Cannioto R. (2018) | Case-control | Cannioto R.  2018 | USA | 590 | 39 | 68.3 (8.6) | 53.8% | White 36 (92.3%) Missing 3 (7.7%) | Mean follow-up 40.7 months | Questionnaire | Physical activity | Age, sex, smoking, BMI, family history of lung cancer |
| Chatzidionysiou K. (2022) | Case-control | Chatzidionysiou K.  2022 | Sweden | 1,972 | 6 | Unknown | Unknown |  | Mean follow-up 7.3-7.6 years | Questionnaire | Rheumatoid arthritis | Age, sex, index year, residence |
| Delcoigne B. (2017) | Nested case- control | Delcoigne B.  2017 | Sweden | 485 | 152 | Unknown | 100% |  | 1958 – 2001  Median follow-up 18 years | Medical records and questionnaire | Radiotherapy for breast cancer | Age, region, decade of diagnosis |
| Derouen M.C. (2022) | Cohort | Derouen M.C.  2022 | USA | 889,870 | 884 | 69.6 (estimated) | 100% | Asian 204 (23.1%)  Black 13 (1.5%)  Hispanic 31 (3.5%)  Native Hawaiian/ Pacific Islander 31 (3.5%)  Non-Hispanic white 306 (34.6%) | 2000 - 2013  Median follow-up 4.8 years | Electronic health records | Ethnicity | Age |
| DiMarzio P. (2018) | Cohort | DiMarzio P.  2018 | USA | 6,218 | Unknown | Unknown | 100% |  | Median follow-up 6.17 years | Health records | Radiotherapy for primary breast cancer | Age, race, breast cancer histology, stage, chemotherapy, alcohol intake |
| Kelly-Reif, K. (2022) | Case-cohort | Kelly-Reif K.  2022 | Former Czechoslovakia | 651 | 121 | Unknown | 0% |  | Mean follow-up 13.6 years | Medical records and annual check-ups | Radon exposure | Age, birth cohort, smoking, radon exposure |
| Kim Y.W. (2025) | Cohort | Kim Y.W.  2025 | South Korea | 21,062 | 176 | 65.2 (7.9) | 79.0% | Likely all Korean | 2009 – 2021  Mean follow-up 83.8 month | Health check up | Sex | Age, family history of lung cancer |
| Kyaw T.W. (2024) | Cohort | Kyaw T.W.  2024 | Taiwan | 241,874 | 1,269 | Unknown | Unknown | Unknown | 1994 – 2017  Follow-up 23 years | Questionnaires | Preserved ratio impaired spirometry (PRISm)  COPD | Age, sex, education, smoking, alcohol, BMI, physical activity, hypertension, diabetes |
| Li J. (2020) | Cohort | Li J.  2020 | China | 17,789 | 152 | 61.9 | 78.9% | Chinese 100% | 2008 - 2012.  Median follow-up 9.1 years | In-person interview | Environmental tobacco smoke exposure | Age, education, personal income, BMI, alcohol intake, fruit and vegetable intake, family history of cancer |

# Appendix 4: Summary of meta-analyses

| **Factor** | **Adjusted hazard ratio (95% CI)** | **Num-ber of stu-dies** | **Number of never-smokers** | **p-value** | ***I^2^* (%)** | **Het-p** | **tau2** |  |
| --- | --- | --- | --- | --- | --- | --- | --- | --- |
| **Factors associated with women** | | | | | | | |  |
| Female sex | **1.28**  **(1.12-1.47)** | 6 of 7 | 597,184 | 0.005 | 0.00 | 0.728 | <0.0001 |  |
| Hormone replacement therapy | 1.01  (0.92-1.10) | 3 | 4,457,796 | 0.88 | 0.00 | 0.416 | 0.0018 |  |
| Age at birth of first child | <25: Ref | 2 of 3 studies | 610,301 | - | - | - | - |  |
|  | ≥25-<30: 1.02  (0.59-1.77) |  |  | 0.94 | 79.50 | 0.027 | 0.1289 |  |
|  | ≥30: 1.20  (0.98-1.46) |  |  | 0.08 | 0.00 | 0.361 | <0.0001 |  |
| Breastfeeding | **0.94**  **(0.90-0.98)** | 3 | 4,907,697 | 0.008 | 0.00 | 0.657 | <0.0001 |  |
| Hormonal contraception | 1.02  (0.98-1.06) | 2 | 4,415,737 | 0.41 | 0.00 | 0.989 | <0.0001 |  |
| **Respiratory comorbidities** | | | | | | | | |
| COPD/obstructive spirometry | 1.54  (0.94-2.54) | 5 | 997,817 | 0.07 | 85.50 | <0.0001 | 0.1277 |  |
| **Other comorbidities** | | | | | | | |  |
| Cancer | **2.04**  **(1.95-2.13)** | 3 | 6,419,722 | <0.0001 | 24.80 | 0.265 | <0.0001 |  |
| Rheumatoid arthritis | **1.41**  **(1.15-1.73)** | 3 | 281,432 | 0.001 | 0.00 | 0.412 | 0.0103 |  |
| Primary breast cancer and received radiotherapy | 1.04  (0.64-1.67) | 2 | 6,703 | 0.56 | 80.60 | 0.023 | 0.0512 |  |
| Cardiovascular disease | 1.21  (0.89-1.66) | 2 | 380,576 | 0.23 | 14.80 | 0.279 | 0.0083 |  |
| Hypertension | **1.16**  **(1.01-1.34)** | 3 | 5,260,101 | 0.04 | 93.50 | <0.0001 | 0.0121 |  |
| Hyper-lipidaemia | 1.12  (0.86-1.46) | 2 | 5,129,542 | 0.40 | 98.30 | <0.0001 | 0.0359 |  |
| Diabetes | 1.04  (0.83-1.31) | 4 | 1,357,714 | 0.74 | 74.10 | 0.009 | 0.0329 |  |
| Peridontitis | 1.78  (0.76-4.16) | 3 | 127,891 | 0.19 | 93.40 | <0.0001 | 0.4909 |  |
| Edentulism | 1.16  (0.90-1.49) | 2 | 44,213 | 0.27 | 0.00 | 0.846 | <0.0001 |  |
| **Family history** | | | | | | | |  |
| Family history of lung cancer | **1.24**  **(0.79-1.95)** | 6 | 1,271,413 | 0.28 | 73.6 | 0.002 | 0.1310 |  |
| **Sociodemographic factors** | | | | | | | |  |
| Lower educational attainment | **1.16**  **(0.97-1.38)** | 4 | 1,003,858 | 0.10 | 57.10 | 0.072 | 0.0150 |  |
| **Environmental factors** | | | | | | | |  |
| Passive smoking | **1.30**  **(1.22-1.40)** | 6 | 1,075,358 | <0.0001 | 0.00 | 0.549 | <0.0001 |  |
| PM10 | **1.10**  **(1.09-1.11)** | 2 | 3,782,684 | <0.001 | 0.00 | 0.875 | <0.0001 |  |
| PM2.5 | **1.16**  **(1.03-1.30)** | 7 | 839,168 | 0.02 | 6.50 | 0.378 | 0.0055 |  |
| NO2 | 0.92  (0.81-1.05) | 2 of 3 studies | 121,373 | 0.22 | 0.00 | 1.000 | <0.0001 |  |
| Urban residence | 1.04  (0.84-1.28) | 3 of 4 studies | 6,238,814 | 0.71 | 92.90 | <0.0001 | 0.0233 |  |
| **Dietary intake** | | | | | | | |  |
| Glycaemic index | Q1: Ref | 2 | Unknown | - | - | - | - |  |
|  | Q2: 0.96 (0.61-1.51) |  |  | 0.847 | 55.40 | 0.134 | 0.0674 |  |
|  | Q3: 1.08  (0.87-1.35) |  |  | 0.483 | 0.00 | 0.758 | <0.0001 |  |
|  | Q4: 1.12 (0.90-1.41) |  |  | 0.317 | 0.00 | 0.616 | <0.0001 |  |
| Fruit intake | 1-2 servings:  1.01  (0.67-1.53) | 3 of 4 studies | At least 163,488 | 0.95 | 0.00 | 0.985 | <0.0001 |  |
|  | >2: 1.21  (0.75-1.94) |  |  | 0.44 | 0.00 | 0.857 | <0.0001 |  |
| Offal | 1.00  (0.85-1.17) | 2 | 195,643 | 0.96 | 35.60 | 0.213 | 0.0047 |  |
| Retinol | 0.96  (0.86-1.07) | 2 | 195,643 | 0.46 | 0.00 | 0.641 | <0.0001 |  |
| Fibre | 0.98  (0.89-1.8) | 2 | 195,643 | 0.70 | 0.00 | 0.840 | <0.0001 |  |
| Vitamin C | 1.05  (0.94-1.17) | 2 | 195,643 | 0.37 | 0.00 | 1.000 | <0.0001 |  |
| Glycaemic load | Q1: Ref | 2 | Unknown | - | - | - | - |  |
|  | Q2: 0.92 (0.74-1.16) |  |  | 0.488 | 0.00 | 0.721 | <0.0001 |  |
|  | Q3: 1.03  (0.82-1.28) |  |  | 0.818 | 0.00 | 0.901 | <0.0001 |  |
|  | Q4: 1.02  (0.78-1.34) |  |  | 0.868 | 12.10 | 0.286 | 0.0079 |  |
| Carbohydrates | Q1: Ref | 2 | Unknown | - | - | - | - |  |
|  | Q2: 1.12 (0.90-1.40) |  |  | 0.322 | 0.00 | 0.717 | <0.0001 |  |
|  | Q3: 1.08 (0.86-1.36) |  |  | 0.495 | 0.00 | 0.498 | <0.0001 |  |
|  | Q4: 1.12 (0.81-1.53) |  |  | 0.491 | 24.50 | 0.250 | 0.0182 |  |
| **Laboratory tests** | | | | | | | |  |
| Mosaic loss of chromosome Y | 0.99  (0.75-1.32) | 2 | 117,392 | 0.97 | 17.40 | 0.271 | 0.0109 |  |
| Clonal haemato-poiesis | 1.55  (1.00-2.41)^%^ | 2 | 1,273 | 0.05 | 0.00 | 0.849 | <0.0001 |  |

^%^Odds ratio

# Appendix 5: Effect measures for all factors and studies

*Factors associated with women*

| **Factor** | **Study name** | **Hazard ratio (95%CI)** | **p-value** | **Total number of never-smokers** | **Total risk of bias score** |
| --- | --- | --- | --- | --- | --- |
| Female sex | 45 and Up | 1.03 (0.67-1.60) | 0.88 | 132,354 | 6 |
|  | PLCO | Male: 0.73 (0.51-1.04) | 0.08 | 49,569 | 7 |
|  | UK Biobank | **Male: 0.67 (0.48-0.94)** | **0.02** | 222,274 | 9 |
|  | GCS | Male: 0.88 (0.42-1.83) | 0.73 | 41,366 | 9 |
|  | MJ Health Database | **1.33 (1.07-1.64)** | **0.009** | 130,559 | 7 |
|  | Kim Y.W. (2025) | Male: 0.90 (0.64-1.26) | 0.53 | 21,062 | 8 |
|  | THIN UK | **Male: 1.62 (1.50-1.75)**** | **<0.0001** | 3,679,831 | 7 |
| Breastfeeding | NHIS | 0.95 (0.90-1.00) | **0.03** | 4,335,259 | 6 |
|  | CanSPUC | 0.91 (0.80-1.04) | 0.17 | 529,823 | 5 |
|  | JPHC | 0.83 (0.60-1.14) | 0.26 | 42,615 | 6 |
| Hormonal contraception | NHIS | 1.02 (0.98-1.06) | 0.42 | 4,335,259 | 6 |
|  | 45 and Up | 1.02 (0.68-1.52) | 0.95 | 80,478 | 6 |
| Hormone replacement therapy | NHIS | 1.03 (0.98-1.08) | 0.23 | 4,335,259 | 6 |
|  | 45 and Up | Never user: Reference  Former: 0.82 (0.51-1.32)  Current: 0.90 (0.44-1.86) | 0.73 | 80,478 | 6 |
|  | PLCO | Former: 0.82 (0.56-1.19) (estimated)  Current: 1.00 (0.70-1.43) | 1.00  0.31 | 42,059 | 6 |
| Age at birth of first child | CanSPUC | Nulliparous: 1.07 (0.69-1.64)  <25: 1.00 (ref)  **25-29: 1.29 (1.14-1.45)**  ≥ 30: 1.23 (0.99-1.51) | 0.77  **<0.0001**  0.05 | 529,823 | 5 |
|  | 45 and Up | <25: 1.00 (ref)  ≥25-30: 0.73 (0.45-1.20)  ≥ 30: 0.88 (0.44-1.74) | 0.60 | 80,478 | 6 |
|  | JPHC | ≤23: 1.00 (ref)  24-26: 1.23 (0.96-1.56)  ≥ 27: 1.09 (0.82-1.43) | 0.09  0.55 | 42,615 | 6 |
| Age at menarche | NHIS | ≤14: Ref  15-16: 1.01 (0.96-1.07)  17: 0.99 (0.93-1.05)  ≥18: 1.03 (0.98-1.09) | 0.73  0.76  0.28 | 4,335,259 | 6 |
|  | CanSPUC | <13: Ref  13: 1.11 (0.90-1.37)  14: 1.15 (0.93-1.40)  **15: 1.24 (1.01-1.53)**  **≥16: 1.46 (1.19-1.78)** | 0.34  0.18  **0.04**  **0.0003** | 529,823 | 5 |
|  | JPHC | ≤13: Ref  **14-15: 1.33 (1.01-1.76)**  ≥16: 1.05 (0.76-1.46) | **0.04**  0.78 | 42,615 | 6 |
| Parity | NHIS | 0: Ref  1: 1.01 (0.88-1.16)  ≥2: 0.91 (0.81-1.04) | 0.90  0.14 | 4,335,259 | 6 |
|  | 45 and Up | 0: 1.99 (0.53-7.56)  1: Ref  2: 1.19 (0.49-2.92)  ≥3: 1.44 (0.60-3.47) | 0.65 | 80,478 | 6 |
|  | JPHC | 1-2: Ref  3: 0.92 (0.71-1.19)  4: 0.98 (0.73-1.32) | 0.54  0.90 | 42,615 | 6 |
| Reproductive period | NHIS | < 30y: Ref  30-34y: 0.99 (0.95-1.04)  35-39y: 1.00 (0.95-1.05)  ≥40y: 1.06 (0.99-1.14) | 0.68  1.00  0.11 | 4,335,259 | 6 |
|  | JPHC | ≤32y: Ref  33-35: 1.09 (0.82-1.46)  ≥36: 1.32 (0.99-1.75) | 0.57  0.06 | 42,615 | 6 |
| Menopause age | NHIS | <40: Ref  40-44: 0.99 (0.87-1.13)  45-49: 0.96 (0.85-1.08)  50-54: 0.98 (0.88-1.10)  ≥55: 1.04 (0.93-1.18) | 0.89  0.51  0.74  0.53 | 4,335,259 | 6 |
|  | CanSPUC | <45: Ref  45-49: 0.84 (0.65-1.09)  50-54: 0.92 (0.72-1.18)  ≥55: 0.97 (0.70-1.35) | 0.19  0.52  0.87 | 529,823 | 5 |
|  | 45 and Up | <45: Ref  45-50: 1.04 (0.49-2.23)  ≥50: 0.74 (0.37-1.50) | 0.16 | 80,478 | 6 |
|  | JPHC | ≤47: Ref  48-50: 1.01 (0.75-1.36)  ≥51: 1.21 (0.91-1.60) | 0.94  0.19 | 42,615 | 6 |
|  | PLCO | <45: 1.04 (0.56-1.96)  45-54: Ref  ≥55: 0.75 (0.41-1.37) | 0.89  0.34 | 38,883 | 6 |
| Menopausal status | CanSPUC | Natural menopause: Ref  **Surgical: 1.67 (1.32-2.11)** | **0.0002** | 529,823 | 5 |
|  | JPHC | Premenopausal: Ref  Natural: 1.46 (0.85-2.50)  **Surgical: 1.99 (1.10-3.59)** | 0.17  **0.02** | 42,615 | 6 |
| Benign breast disease | CanSPUC | **1.24 (1.10-1.41)** | **0.0007** | 529,823 | 5 |
| Length of menstrual cycle (days) | JPHC | ≤26: 0.97 (0.71-1.32)  27-29: 1.00 (ref)  ≥30: 0.92 (0.71-1.18) | 0.86  0.53 | 42,615 | 6 |
| Exogenous hormone use | JPHC | 1.15 (0.85-1.56) | 0.37 | 42,615 | 6 |
| Surgery on reproductive system | CanSPUC | 1.10 (0.94-1.27) | 0.22 | 529,823 | 5 |

**Incidence rate ratio

*Respiratory comorbidities*

| **Factor** | **Study name** | **Hazard ratio (95%CI)** | **p-value** | **Total number of never-smokers** | **Total risk of bias score** |
| --- | --- | --- | --- | --- | --- |
| COPD/obstructive spirometry | 45 and Up | 1.63 (0.77-3.44) | 0.20 | 132,354 | 6 |
|  | NHIS | **2.67 (2.09-3.40)** | **<0.0001** | 241,633 | 7 |
|  | Lifelines | 0.90 (0.40-2.10) | 0.81 | 45,430 | 8 |
|  | CKB | **1.48 (1.22‐1.81)** | **<0.001** | 336,526 | 8 |
|  | Kyaw T.W. (2024) | 1.16 (0.93-1.45) | 0.19 | 241, 874 | 8 |
| Asthma | 45 and Up | 1.35 (0.73-2.50) | 0.33 | 132,354 | 6 |
| PRISm | Kyaw T.W. (2024) | 1.08 (0.93-1.24) | 0.34 | 241, 874 | 8 |
| Pleural plaques | ARDCO | **3.13 (1.04-9.35)** | **0.04** | 1,305 | 9 |
| Bronchiectasis | NHIS | **1.44 (1.31-1.57)** | **<0.001** | 2,323,605 | 9 |
| Pulmonary tuberculosis | NHIS | **1.42 (1.04-1.95)**  Without COPD:  0.91 (0.62-1.33) | **0.03**  0.64 | 32,086 | 7 |
| Obstructive sleep apnoea | NHS | **2.96 (1.42-6.18)** | **0.004** | 31,323 | 6 |
| FEV1/FVC (per 5% increase) | UK Biobank | 0.93 (0.84-1.04) | 0.18 | 222,274 | 9 |
| Emphysema | WHI | 1.13 (0.53-2.39)* | 0.76 | 65,419 | 8 |
| Chronic respiratory disease | CanSPUC | **1.94 (1.24-3.04)** | **0.004** | 75,917 | 7 |
| Primary lung cancer | MEC | **Standardised incidence rate 14.50 (8.73-22.65)** | N/A | 94,579 | 7 |

*Other comorbidities*

| **Factor** | **Study name** | **Hazard ratio (95% CI)** | **p-value** | **Total number of never-smokers** | **Total risk of bias score** |
| --- | --- | --- | --- | --- | --- |
| Any cancer | UK Biobank | 1.39 (0.87-2.24) | 0.17 | 222,274 | 9 |
|  | CKB | **2.21 (1.57‐3.10)** | **<0.001** | 336,526 | 8 |
|  | NHIS | Uterine, ovarian, cervical cancer: **1.98 (1.66-2.36)**  Breast: **2.25 (1.92-2.62)**  Other: **2.03 (1.93-2.13)** | **<0.0001**  **<0.0001**  **<0.0001** | 5,860,922 | 9 |
| Rheumatoid arthritis | Chatzidionysiou K. (2022) | 1.40 (0.25-7.77) | 0.71 | 1,972 | 8 |
|  | NHIS | **1.31 (1.13-1.53)** | **0.0005** | 247,688 | 8 |
|  | Veterans Health Administration | **1.65 (1.22–2.24)** | **0.001** | 31,772 | 7 |
| Primary breast cancer and received radiotherapy | Delcoigne B. (2017) | 0.80 (0.57-1.13) | 0.20 | 485 | 9 |
|  | DiMarzio P. (2018) | **1.30 (1.02-1.66)** | **0.03** | 6,218 | 6 |
| Cardiovascular disease | UK Biobank | 0.98 (0.60-1.61) | 0.94 | 250,017 | 8 |
|  | MJ Health Database | 1.37 (0.96-1.94) | 0.08 | 130,559 | 7 |
| Hypertension | CanSPUC | **1.28 (1.20-1.36)** | **<0.0001** | 794,283 | 5 |
|  | NHIS | **1.05 (1.01-1.08)** | **0.004** | 4,335,259 | 6 |
|  | MJ Health Database | 1.17 (0.90-1.52) | 0.26 | 130,559 | 7 |
| Hyperlipidaemia | CanSPUC | **1.28 (1.21-1.37)** | **<0.0001** | 794,283 | 5 |
|  | NHIS | 0.98 (0.95-1.01) | 0.20 | 4,335,259 | 6 |
| Diabetes | CanSPUC | **1.29 (1.21-1.37)** | **<0.0001** | 794,283 | 5 |
|  | NHIS | 0.91 (0.71-1.17) | 0.47 | 300,518 | 9 |
|  | MJ Health Database | 0.92 (0.64-1.33) | 0.66 | 130,559 | 7 |
|  | 45 and Up | 0.87 (0.52-1.46) | 0.87 | 132,354 | 6 |
| Peridontitis or poor dental health | ARIC | 1.70 (0.75-3.90) | 0.21 | 2,847 | 8 |
|  | WHI | 0.89 (0.59-1.33) | 0.59 | 34,097 | 6 |
|  | NHIS | **3.56 (2.68-4.72)** | **<0.0001** | 90,947 | 8 |
| Edentulism | ARIC | 0.98 (0.18-5.25) | 0.98 | 2,847 | 8 |
|  | GCS | Tooth loss: 1.16 (0.90-1.51)  Number of decayed, missing or filled teeth: 1.31 (0.97-1.76) | 0.26  0.082 | 41,366 | 9 |
| Psychological comorbidities | NHS (symptoms per 1-SD increase) | Depression:  1.07 (0.90-1.28) | 0.46 | 129 | 7 |
|  | UK Biobank | Psychological distress (positive PHQ-4):  1.10 (0.97-1.24) | 0.14 | 223,682 | 8 |
| Cerebrovascular disease | MJ Health Database | 1.00 (0.32-3.15) | 0.996 | 130,559 | 7 |
| Peripheral arterial disease | ARIC | **Symptomatic:**  **9.08 (1.16-71.34)**  Asymptomatic:  1.59 (0.35-7.20) | **0.04**  0.56 | 5,548 | 8 |
| Hepatobiliary disease | CanSPUC | **1.29 (1.21-1.37)** | **<0.0001** | 794,283 | 5 |
| Gastro-intestinal diseases | CanSPUC | **1.30 (1.22-1.38)** | **<0.0001** | 794,283 | 5 |
| Gastro-oesophageal reflux disease | UK Biobank | 0.89 (0.70-1.14) | 0.35 | 275,811 | 8 |
| Knee osteoarthritis | SIDIAP | 0.98 (0.76-1.27) | 0.89 | 405,566 | 8 |
| Oral microbiome | SMHS and SWHS | Some associated with increased risk e.g. *Lactobacillales* order medium vs. low OR 2.15 (1.03-4.47), some with decreased e.g. *Bacteroidetes* order high vs. low OR 0.31 (0.15-0.64) | Various | 180 | 8 |

*Medication*

| **Factor** | **Study name** | **Hazard ratio (95%CI)** | **p-value** | **Total number of never-smokers** | **Total risk of bias score** |
| --- | --- | --- | --- | --- | --- |
| Anti-hypertensives | 45 and Up | Nil: Ref  ACE-I: 1.24 (0.77-2.01)  ARB: 1.06 (0.65-1.73)  CCB: 1.36 (0.68-2.70)  ≥2: 1.17 (0.54-2.54) | 0.92 | 132,354 | 6 |
|  | NHIS | CCB: Ref  **ARB: 0.64 (0.42-0.99)** | **0.04** | 39,784 | 9 |
| Statin use | NHIS | 1.04 (0.93-1.16) | 0.50 | 515,100 | 9 |
| Calcium and vitamin D supplementation | WHI CaD | 1.26 (0.63-2.55) | 0.53 | 18,753 | 8 |
| Oral bisphosphonate | WHI | **0.57 (0.39-0.84)** | **0.004** | 75,962 | 7 |
| Aspirin for cardiovascular use | NHIS | 0.97 (0.89-1.07) | 0.53 | 515,100 | 9 |
| Aspirin and statin | NHIS | E.g. use ≥547.5 days:  1.03 (0.75-1.41) | 0.86 | 515,100 | 9 |
| Metformin use for diabetes | NHIS | **Compared to non-diabetics:**  **0.86 (0.74-0.99)**  Compared to diabetics who had never used metformin:  0.87 (0.71-1.07) | **0.04**  0.18 | 515,100 | 9 |
| Aspirin and metformin | NHIS | Use ≥547.5 days:  0.93 (0.59-1.46) | 0.77 | 515,100 | 9 |
| Statin and metformin | NHIS | Use ≥547.5 days:  1.03 (0.55-1.93) | 0.93 | 515,100 | 9 |
| Aspirin, statin and metformin | NHIS | Use ≥547.5 days:  **0.38 (0.20-0.73)** | **0.003** | 515,100 | 9 |

*Family history of cancer*

| **Factor** | **Study name** | **Hazard ratio (95%CI)** | **p-value** | **Total number of never-smokers** | **Total risk of bias score** |
| --- | --- | --- | --- | --- | --- |
| Family history of lung cancer | 45 and Up | 1.22 (0.78-1.93) | 0.39 | 132,354 | 6 |
|  | CanSPUC | **1.50 (1.29-1.75)** | **<0.001** | 547,218 | 6 |
|  | UK Biobank | 0.85 (0.53-1.38) | 0.52 | 222,274 | 9 |
|  | Kangbuk Samsung Cohort Study | **1.93 (1.24-3.01)** | **0.004** | 189,439 | 7 |
|  | MJ Health Database | **1.68 (1.20-2.37)** | **<0.001** | 130,559 | 7 |
|  | PLCO | **0.59 (0.34-1.00)** | **0.048** | 49,569 | 7 |
| Number of relatives with lung cancer | CanSPUC | **1.31 (1.24-1.39)** | **<0.0001** | 794,283 | 5 |
| Father | CanSPUC | **2.15 (1.66-2.77)** | **<0.0001** | 547,218 | 6 |
| Mother | CanSPUC | **1.33 (1.14-1.57)** | **0.0005** | 547,218 | 6 |
| Siblings | CanSPUC | **2.02 (1.30-3.12)** | **0.002** | 547,218 | 6 |
| Family history of any cancer | CanSPUC | **1.31 (1.24-1.40)** | **<0.0001** | 794,283 | 5 |

*Sociodemographic factors*

| **Factor** | **Study name** | **Hazard ratio (95%CI)** | **p-value** | **Total number of never-smokers** | **Total risk of bias score** |
| --- | --- | --- | --- | --- | --- |
| Education | BWHS | College degree & above: Ref  Some college:  1.06 (0.65-1.73)  High school:  1.27 (0.71-2.28) | 0.83  0.43 | 37,650 | 5 |
|  | CanSPUC | **1.30 (1.23-1.38)** | **<0.0001** | 794,283 | 5 |
|  | GCS | Any: 1.71 (0.73-3.98) | 0.22 | 41,366 | 9 |
|  | MJ Health Database | Lower: 1.05 (0.84-1.31) | 0.65 | 130,559 | 7 |
| Income | BWHS (annual family income) | <25,000: 0.74 (0.31-1.80)  25,000-50,000: 0.68 (0.33-1.37) 50,000-100,000: 0.67 (0.34-1.34)  >100,0000: Reference | 0.51  0.29  0.26 | 37,650 | 5 |
|  | NHIS | Q1: 1.00 (0.96-1.04)  Q2: 0.96 (0.92-1.00)  Q3: 0.97 (0.93-1.01)  Q4 (highest): 1 (ref.) | 1.00  0.09  0.15 | 4,525,203 | 6 |
| Deprivation | BWHS | **1.30 (1.04-1.63)** per 10 unit increase of neighbourhood Concentrated Disadvantage Index | **0.02** | 37,650 | 5 |
|  | THIN | Women**:  1 (least deprived): Ref  2: 1.01 (0.88-1.16)  3: 0.92 (0.79-1.07)  4: 0.98 (0.83-1.15)  5: 1.13 (0.94-1.36)  Men**:  1 (least deprived): Ref  2: 0.98 (0.84-1.15)  3: 1.01 (0.85-1.20)  **4: 1.24 (1.04**-**1.48)**  **5: 1.42 (1.15**-**1.76)** | 0.31  **0.002** | 3,679,831 | 7 |
|  | GCS | Quartile 1 (low): Ref  Q2: 1.01 (0.45-2.26)  Q3: 0.72 (0.30-1.73)  Q4: 0.94 (0.39-2.29) | 0.99  0.47  0.90 | 41,366 | 9 |
| Ethnicity | Derouen M.C. (2022) | Non-Hispanic white: Ref  AANHPI: **1.69 (1.38-2.02)**  Black: Not available  Hispanic: 0.84 (0.56-1.23)** | **<0.0001**  0.39 | 889,870 | 6 |
|  | PLCO | Non-Hispanic white: Ref  Hispanic: 0.76 (0.11-5.58)  Asian: 1.00 (0.41-2.50) | 0.79  0.995 | 49,569 | 7 |
| Country of birth | 45 and Up | **Asian: 2.83 (1.64-4.89)** | **0.0002** | 132,354 | 6 |
| Health insurance | BWHS | No: 1.18 (0.41-3.36) | 0.77 | 37,650 | 5 |
| Adventist | AHS-2 | 0.74 (0.53-1.03) | 0.08 | 64,700 | 7 |

**Incidence rate ratio

*Physical measurements*

| **Factor** | **Study name** | **Hazard ratio (95%CI)** | **p-value** | **Total number of never-smokers** | **Total risk of bias score** |
| --- | --- | --- | --- | --- | --- |
| BMI | BWHS | 1.01 (0.97-1.05)  per unit increase | 0.64 | 37,650 | 5 |
|  | SIDIAP | **0.95 (0.91-1.00)**  per 5kg/m^2^ increase | **0.03** | 2,339,363 | 8 |
|  | 45 and Up | **<18.5: 1.98 (1.16-3.39)**  18.5-25: Ref  ≥25-<30: 0.92 (0.65-1.30)  ≥30: 1.01 (0.64-1.59) | 0.05 | 132,354 | 6 |
|  | NHIS | <18.5: 0.93 (0.83-1.03)  18.5-<23: Ref  **23-<25: 0.95 (0.91-0.99)**  **25-<30: 0.95 (0.91-0.99)**  ≥30: 0.97 (0.90-1.05) | 0.19  **0.02**  **0.02**  0.45 | 4,335,259 | 6 |
|  | JPHC | <19: 0.86 (0.56-1.33)  19-22.9: 0.98 (0.63-1.52)  23-25: Ref  25-26.9: 0.86 (0.51-1.46)  >27: 0.65 (0.33-1.29) | 0.52  0.93  0.59  0.22 | 54,950 | 8 |
|  | CKB | **<18.5: 1.45 (1.21‐1.73)**  **18.5-23.9: 1.17 (1.08‐1.27)**  ≥24: Ref | **<0.001**  **<0.001** | 336,526 | 8 |
|  | MJ Health Database | <18.5: 0.77 (0.50-1.18)  18.5-23.9: Ref  **24-27.9: 0.80** **(0.64-0.99)**  ≥28: 0.72 (0.51-1.03) | 0.23  **0.04**  0.08 | 130,559 | 7 |
|  | CanPath | <25: Ref  25-29.9: 0.77 (0.46-1.28)  >30: 0.62 (0.35-1.09)^%^ | 0.32  0.10 | 950 | 7 |
|  | CPS-II | <18.5: 1.30 (0.60-2.84)  18.5-22: Ref  22.1-24.9: 1.00 (0.75-1.34)  25-29.9: 1.04 (0.79-1.37)  30+: 0.85 (0.60-1.21)* | 0.52  1.00  0.79  0.37 | Unknown | 9 |
| BMI change | Kailuan | Major loss <1.0 kg/m^2^/year:  **1.97 (1.12-3.45)**  Major gain ≥1.0 kg/m^2^/year:  **2.15 (1.15-4.02)** | **0.02**  **0.02** | 37,085 | 7 |
| Height | JPHC | Q1: 1.03 (0.82-1.28)  Q2: Ref  Q3: 0.96 (0.75-1.23)  Q4: 0.99 (0.78-1.26) | 0.84  0.74  0.94 | 54,950 | 8 |
|  | Miyagi | 1.07 (0.81-1.41)  per 10cm increment | 0.64 | 18,907 | 9 |
|  | NHIS | **1.10 (1.09-1.11)**  **per 5cm increment** | **<0.0001** | Unknown | 7 |
|  | CKB | <150cm: Ref  **150-154: 1.14 (1.01‐1.27)**  **155-159: 1.15 (1.02-1.30)**  **≥160: 1.26 (1.12‐1.42)** | **0.03**  **0.02**  **<0.001** | 336,526 | 8 |
|  | 45 and Up | 1.01 (0.98-1.03) | 0.67 | 132,354 | 6 |
| Waist circumference | CanPath | >102cm males or >88cm females: 0.96 (0.61-1.50)^%^ | 0.87 | 950 | 7 |
|  | CPS-II | Quartile 1 (Q1): Ref  Q2: 1.23 (0.88-1.71)  Q3: 1.10 (0.77-1.57)  Q4: 1.27 (0.89-1.82)* | 0.22  0.61  0.19 | Unknown | 9 |
| Weight change | JPHC | Decrease: 1.08 (0.79-1.47)  Unchanged: Ref  Increase: 0.99 (0.79-1.25) | 0.64  0.96 | 54,950 | 8 |
| Waist-to-hip ratio | CanPath | ≥1 males or ≥0.86 females:  1.11 (0.72-1.71)^%^ | 0.22 | 950 | 7 |

*Relative risk ^%^Odds ratio

*Lifestyle factors*

| **Factor** | **Study name** | **Hazard ratio (95%CI)** | **p-value** | **Total number of never-smokers** | **Total risk of bias score** |
| --- | --- | --- | --- | --- | --- |
| Physical activity | NHIS | 0.97 (0.94-1.01) | 0.10 | 4,335,259 | 6 |
|  | CKB | **0.83 (0.71-0.97) per 1-SD increment** | **0.02** | 32,186 | 9 |
|  | 45 and Up | Nil: Ref  0-150min: 1.06 (0.53-2.12)  150-300 min: 0.75 (0.35-1.59)  ≥300 min: 1.10 (0.58-2.10) | 0.49 | 132,354 | 6 |
|  | BWHS | Tertile 1: Ref Tertile 2: 0.79 (0.37-1.65) Tertile 3: 1.10 (0.54-2.24) | 0.55  0.80 | Unknown | 7 |
|  | NOWAC | 1-2 decile: 1.39 (0.41-4.71) 3-4: 1.23 (0.63-2.42) 5-6: Ref  7-8: 1.26 (0.66-2.40) 9-10: 0.45 (0.06-3.34) | 0.61  0.56  0.49  0.44 | Unknown | 5 |
|  | CanPath | 1.27 (0.80-2.02)^%^ | 0.26 | 950 | 7 |
|  | Cannioto, R. (2018) | **Inactive: 3.00 (1.33-6.78)^%^** | **0.008** | 590 | 7 |
|  | CPS-II | 0 METS: 1.13 (0.82-1.55) 0-<8.75: Ref 8.75-17.4: 0.77 (0.59-1.00) 17.5+: 0.92 (0.74-1.15)* | 0.46  0.05  0.47 | Unknown | 9 |
| Alcohol | 45 and Up | 0-<1 drinks/week:  1.07 (0.67-1.70)  ≥1-≤3.5: Ref  >3.5-≤7: 0.94 (0.54-1.63)  >7: 1.16 (0.69-1.96) | 0.94 | 132,354 | 6 |
|  | CanPath | 1.06 (0.50-1.62)^%^ | 0.86 | 950 | 7 |
|  | CanSPUC | **1.30 (1.22-1.38)** | **<0.0001** | 794,283 | 5 |
|  | NHIS | None: Ref  Mild: 1.04 (0.98-1.10)  Heavy: 1.21 (0.90-1.64) | 0.18  0.21 | 4,335,259 | 6 |
|  | EPIC  (Beer/cider) | 0.96 (0.79-1.17) | 0.70 | 194,087 | 9 |
|  | NLCS (Beer/cider) | 1.12 (0.81-1.55) | 0.50 | 1,556 | 9 |
| Walking for exercise >1hour/week | BWHS | 1.73 (0.92-3.24) | 0.09 | 38,432 | 7 |
| Sitting and watching television >1hour/week | BWHS | 1.99 (0.58-8.29) | 0.32 | 38,432 | 7 |
| Sleep quality | Lianyungang | 1.21 (0.62-2.39)^%^ | 0.59 | 182 | 8 |
| Sleep duration (<6 or >9 hours) | CanPath | **1.52 (1.01–2.29)^%^** | **0.0008** | 950 | 7 |
| 2018 World Cancer Research Fund/American Institute for Cancer Research Score calculated from weight, physical activity, diet, alcohol intake | NIH-AARP Diet and Health Study | Males: 1.12 (0.92-1.37)  Females: 0.92 (0.79-1.08) | 0.27  0.30 | 86,242 | 8 |

*Relative risk ^%^Odds ratio

*Environmental factors*

| **Factor** | **Study name** | **Hazard ratio (95%CI)** | **p-value** | **Total number of never-smokers** | **Total risk of bias score** |
| --- | --- | --- | --- | --- | --- |
| Passive smoking | 45 and Up | 0.97 (0.69-1.37) | 0.87 | 132,354 | 6 |
|  | CanSPUC | **1.31 (1.23-1.39)** | **<0.0001** | 794,283 | 5 |
|  | BWHS | **1.56 (1.09-2.24)** | **0.02** | 37,650 | 5 |
|  | PLCO | Work: 1.31 (0.98-1.76)  Home: 1.34 (1.00-1.79) | 0.07  0.054 | 49,569 | 7 |
|  | NOWAC | 1.34 (0.89-2.01) | 0.16 | 43,713 | 7 |
|  | Li J. (2020) | 1.40 (0.96-2.05) | 0.08 | 17,789 | 7 |
| In utero exposure to secondhand smoke | UK Biobank | 1.09 (0.82-1.44) | 0.56 | 250,043 | 8 |
| Childhood exposure to secondhand smoke | PLCO | 0.92 (0.68-1.26) | 0.62 | 49,569 | 7 |
| PM10 | NHIS | **1.10 (1.09-1.11)** | **<0.0001** | 3,553,833 | 8 |
|  | UK Biobank | 1.16 (0.60-2.26) | 0.67 | 228,851 | 9 |
| PM2.5 | WHI | Yearly: 0.85 (0.53-1.36)  Year 2000 cumulative:  1.09 (0.74-1.61) | 0.51  0.68 | 65,419 | 8 |
|  | NHIS | 0.71 (0.32-1.58) | 0.41 | 55,954 | 9 |
|  | AHSMOG-2 | 1.32 (0.90-1.93) | 0.15 | 64,906 | 7 |
|  | UK Biobank | 0.89 (0.48-1.65) per 5μg/m3 | 0.71 | 228,851 | 9 |
|  | CKB | **1.10 (1.04-1.17)** | **0.002** | 255,829 | 9 |
|  | MJ Health Database | **1.32 (1.12-1.56)** | **0.001** | 130,559 | 7 |
|  | BWHS | 1.01 (0.95-1.08) per μg/m3 | **0.77** | 37,650 | 5 |
| Nitrogen dioxide (NO2) | WHI | 0.92 (0.74-1.15) per 10ppb | 0.48 | 65,419 | 8 |
|  | NHIS | 0.92 (0.78-1.09) per 10ppb | 0.33 | 55,954 | 9 |
|  | UK Biobank | 0.98 (0.85-1.13) per 10μg/m3 | 0.81 | 246,664 | 9 |
| Urban residence | THIN | Women  Urban: Ref  Village: 0.98 (0.83-1.14)  Rural: 0.92 (0.74-1.13)  Men  Village: 1.09 (0.92-1.30)  Rural: 0.78 (0.61-1.01)** | 0.70  0.07 | 3,679,831 | 7 |
|  | CKB | **Urban: 1.19 (1.09‐1.29)** | **<0.001** | 336,526 | 8 |
|  | GCS | Rural: 1.16 (0.51-2.64) | 0.72 | 41,366 | 9 |
|  | NHIS | **Urban: 0.94 (0.92-0.96)** | **<0.0001** | 5,860,922 | 9 |
| Wood-burning | Sister Study | Used wood as a source of fuel:  1.62 (0.95-2.75)  1-29/days/year: 1.64 (0.87-3.10)  **≥1 month/year: 1.99 (1.02-3.89)** | 0.07  0.13  **0.04** | 28,290 | 7 |
| Distance to A1 (busy London road) (metres) | WHI | >200: Ref  50-<200: 0.82 (0.34-1.98)  **<50: 5.23 (1.94-14.13)** | 0.67  **0.001** | 65,419 | 8 |
| Pesticides e.g. chlorimuron-ethyl | AHS | Non-exposed: Ref  <median: 2.17 (0.92-5.11)  **>median: 3.80 (1.53-9.48)** | 0.08  **0.004** | 26,859 | 7 |
| Occupational exposure to hazardous substances | CanSPUC | **1.29 (1.21-1.37)** | **<0.0001** | 794,283 | 5 |
| Air pollution | CanSPUC | **1.29 (1.22-1.37)** | **<0.0001** | 794,283 | 5 |
| Cooking oil fumes | CanSPUC | **1.29 (1.22-1.37)** | **<0.0001** | 794,283 | 5 |
| Radon | Kelly-Reif, K. (2022) | Working level months (WLM) 10 -<50: 1.27 (0.71-2.27)  50-<100: 0.93 (0.47-1.85)  100-<150: 0.88 (0.42-1.86)  150-<200: 1.55 (0.61-3.92)  200+: 1.79 (0.90-3.57)* | 0.43  0.85  0.75  0.36  0.10 | 651 | 6 |
| Radiation from atomic bomb | Life Span | Excess risk/Gray:  0.81 (0.51-1.18)* | 0.33 | 432 | 7 |

*Relative risk **Incidence rate ratio

*Dietary intake*

| **Factor** | **Study name** | **Hazard ratio (95% CI)** | **p-value** | **Total number of never-smokers** | **Total risk of bias score** |
| --- | --- | --- | --- | --- | --- |
| **Dietary indices** | | | | | |
| Glycaemic index | SWHS | Q1: Ref  Q2: 1.13 (0.89-1.44) Q3: 1.10 (0.86-1.40) Q4: 1.15 (0.90-1.47) | 0.32  0.45  0.27 | Unknown | 9 |
|  | SMHS | Q1: Ref  Q2: 0.69 (0.38-1.26)  Q3: 1.00 (0.57-1.73)  Q4: 0.98 (0.55-1.74) | 0.94 | Unknown | 9 |
| AMED | MEC | 0-2: Ref 3: 1.04 (0.80-1.34) 4: 0.89 (0.69-1.16) 5: 0.83 (0.63-1.09) 6-9: 0.79 (0.60-1.04) | 0.78  0.39  0.18  0.09 | 80,635 | 9 |
|  | WHI | Q1: Ref Q2: 1.26 (0.82-1.92) Q3: 1.14 (0.75-1.75) Q4: 0.87 (0.55-1.38) Q5: 1.02 (0.66-1.59) | 0.29  0.56  0.56  0.94 | 41,950 | 7 |
|  | GCS | 0-3: Ref 3-4: 1.75 (0.90-3.42) 4-7**: 3.28 (1.50-7.13)** | 0.10  **0.003** | Unknown | 9 |
|  | NLCS | 0-3: Ref  4-5: 0.95 (0.67-1.35)  6-8: 0.60 (0.35-1.01) | 0.79  0.06 | Unknown | 9 |
| HEI-2015 | MEC | 17.9-58.2: Ref  58.3-64.6: 1.12 (0.85-1.48)  64.7-70.2: 1.04 (0.79-1.37)  70.3-76.6: 0.82 (0.62-1.09)  76.7-100: 0.94 (0.72-1.24) | 0.43  0.79  0.17  0.67 | 80,635 | 9 |
|  | WHI | Quintile (Q) 1: Ref  Q2: 0.93 (0.61-1.43)  Q3: 1.07 (0.71-1.63)  Q4: 0.85 (0.55-1.31)  Q5: 1.00 (0.65-1.52) | 0.75  0.76  0.47  0.99 | 41,950 | 7 |
|  | GCS | 7-30: Ref 31-37: 1.19 (0.63-2.26) 38-82: 1.84 (0.97-3.50) | 0.61  0.06 | Unknown | 9 |
| HEI-10 | SCCS | Highest quality diet: Ref  Higher: **3.15 (1.59-6.24)**  Lower: **4.14 (2.08-8.21)**  Lowest: **2.70 (1.25-5.83)** | **0.001**  **<0.0001**  **0.01** | 25,860 | 8 |
| AHEI-2010 | MEC | 25.1-56.6: Ref 56.7-62.2: 0.83 (0.64-1.08) 62.3-67.1: 0.82 (0.63 -1.06) 67.2-72.6: 0.91 (0.71-1.18) **72.7-104.5: 0.66 (0.50-0.87)** | 0.16  0.14  0.48  **0.003** | 80,635 | 9 |
|  | WHI | Q1: Ref Q2: 0.69 (0.47-1.02) **Q3: 0.60 (0.40-0.91) Q4: 0.59 (0.39-0.93)** Q5: 0.98 (0.67-1.43) | 0.06  **0.02**  **0.02**  0.92 | 41,950 | 7 |
|  | GCS | T1: Ref  T2: 1.05 (0.57-1.93)  T3: 1.19 (0.63-2.27) | 0.88  0.61 | Unknown | 9 |
| DASH | MEC | 8-20: Ref  21-22: 1.23 (0.94-1.62)  23-25: 0.96 (0.75-1.24)  26-27: 0.94 (0.71-1.24)  28-40: 0.83 (0.64-1.09) | 0.14  0.76  0.68  0.17 | 80,635 | 9 |
|  | WHI | Q1: Ref  Q2: 1.01 (0.65-1.55)  Q3: 1.09 (0.70-1.70)  Q4: 0.94 (0.62-1.44)  Q5: 1.20 (0.80-1.81) | 0.97  0.72  0.79  0.39 | 41,950 | 7 |
|  | GCS | T1: Ref  T2: 0.91 (0.51-1.64)  T3: 0.75 (0.39-1.44) | 0.76  0.39 | Unknown | 9 |
|  | PLCO | Q4 vs. Q1: 1.20 (0.69-2.09) | 0.53 | 24,588 | 7 |
| DII | MEC | 0.46-4.98: Ref -0.94-0.45: 1.03 (0.77-1.39) -2.12- -0.95: 1.04 (0.78-1.39) -3.24- -2.13: 0.96 (0.72 1.28)  -6.44- -3.25: 0.90 (0.68-1.20) | 0.85  0.80  0.79  0.48 | 80,635 | 9 |
| MINDDiet | UK Biobank | 0.95 (0.79-1.14) | 0.60 | 106,872 | 8 |
| **Specific foods** | | | | | |
| Fruit | 45 and Up | ≥0-<1 serves/day: Ref  ≥1-<2: 0.99 (0.42-2.33) ≥2: 1.19 (0.52-2.73) | 0.59 | 132,354 | 6 |
|  | BWHS | <1 servings/day: Ref  1-<2: 0.98 (0.50-1.93)  >2: 0.94 (0.32-2.75) | 0.96  0.92 | 31,134 | 9 |
|  | GCS | <87g/day: Ref  87-165: 1.06 (0.55-2.04)  >165: 1.35 (0.67-2.70) | 0.87  0.41 | Unknown | 9 |
|  | EPIC | 0.95 (0.83-1.10)  per 1-SD increment | 0.48 | 194,087 | 9 |
| Vegetable | 45 and Up | 0-<3 serves/day: Ref  3-5: 1.29 (0.85-1.97)  ≥5: 1.08 (0.70-1.67) | 0.45 | 132,354 | 6 |
|  | BWHS | <1 servings/day: Ref  1-2: 1.22 (0.65-2.70)  >2: 0.7 (0.16-3.01) | 0.60  0.65 | 31,134 | 9 |
|  | GCS | <149g/day: Ref  149-214: 0.77 (0.40-1.47)  >214: 1.18 (0.60-2.29) | 0.44  0.64 | Unknown | 9 |
| Cruciferous vegetable | BWHS | <0.5 servings/day: Ref  0.5-1: 0.71 (0.29-1.75)  >1: 1.49 (0.59-3.79) | 0.46  0.41 | 31,134 | 9 |
|  | JPHC | Quartile (Q) 1: Ref  Q2: 0.84 (0.64-1.09)  Q3: 0.94 (0.74-1.21)  Q4: 0.93 (0.73-1.18) | 0.18  0.66  0.54 | 54,659 | 7 |
| Fruit and vegetable | BWHS | <3 servings/day: Ref 3-<5: 1.12 (0.53-2.39) >5: 0.50 (0.12-2.13) | 0.78  0.34 | 31,134 | 9 |
|  | MJ Health Database | ≥2 servings/day:  1.12 (0.69-1.82) | 0.65 | 130,559 | 7 |
|  | CanPath | **<5/day: 1.5 (1.03-2.17)^%^** | **0.03** | 950 | 7 |
| Red and processed meat | 45 and Up | Red  Never: Ref  >0-≤2/week: 0.89 (0.52-1.50)  >2-≤5: 0.94 (0.58-1.54)  >5: 1.26 (0.69-2.27)  Processed  Never: Ref  **0-≤ 1/week: 0.61 (0.39-0.96)** > 1-≤ 2: 0.99 (0.60-1.62) >2: 0.88 (0.51-1.50) | 0.57  0.12 | 132,354 | 6 |
|  | GCS | <8g/d: Ref  8-19: 1.88 (0.99-3.58)  >19: 1.69 (0.83-3.46) | 0.05  0.15 | Unknown | 9 |
|  | NHIS | Vegetable: Ref  **Vegetable and meat: 1.04 (1.02-1.06)**  **Meat intake: 1.08 (1.01-1.15)** | **<0.0001**  **0.02** | 5,860,922 | 9 |
| Offal | EPIC | 1.07 (0.91-1.26) | 0.42 | 194,087 | 9 |
|  | NLCS | 0.91 (0.75-1.11) | 0.35 | 1,556 | 9 |
| Tea drinking | CKB | Less than weekly: Ref Weekly: 1.06 (0.87-1.30) ≤2g/day: 0.96 (0.81-1.14) 2.1-4.0g/day: 0.95 (0.78-1.16) >4.0g/day: 1.08 (0.84-1.40) | 0.58  0.65  0.62  0.57 | 334,082 | 7 |
| Citrus fruit | BWHS | <1 servings/day: Ref  1-<2: 0.75 (0.34-1.66)  >2: 0.25 (0.03-1.88) | 0.49  0.19 | 31,134 | 9 |
| Non-starchy vegetable | BWHS | <1 servings/day: Ref  1-<2: 1.82 (0.94-3.53) ≥2: 0.96 (0.37-2.51) | 0.08  0.94 | 31,134 | 9 |
| Starchy vegetable | BWHS | <0.5 servings/day: Ref 0.5-1: 1.08 (0.66-1.76) >1: 0.94 (0.34-2.58) | 0.77  0.91 | 31,134 | 9 |
| Low-fat dairy | GCS | <72g/d: Ref  72-150: 0.61 (0.33-1.13)  >150: 0.51 (0.25-1.03) | 0.11  0.06 | Unknown | 9 |
| Nuts | GCS | <10g/d: Ref  10-19: 1.10 (0.60-2.01)  >19: 1.09 (0.57-2.10) | 0.77  0.81 | Unknown | 9 |
| Refined grain | GCS | <350g/d: Ref **350-478: 0.39 (0.19-0.79)** >478: 0.48 (0.21-1.08) | **0.01**  0.08 | Unknown | 9 |
| **Specific nutrients** | | | | | |
| Retinol | EPIC | 0.98 (0.85-1.12) | 0.79 | 194,087 | 9 |
|  | NLCS | 0.93 (0.78-1.10) | 0.42 | 1,556 | 9 |
| Fibre | EPIC | 0.99 (0.86-1.13) | 0.89 | 194,087 | 9 |
|  | NLCS | 0.97 (0.84-1.12) | 0.69 | 1,556 | 9 |
| Vitamin C | EPIC | 1.05 (0.91-1.21) | 0.51 | 194,087 | 9 |
|  | NLCS | 1.05 (0.89-1.23) | 0.57 | 1,556 | 9 |
| Glycaemic load | SWHS | Q1: Ref  Q2: 0.94 (0.74-1.21)  Q3: 1.02 (0.80-1.30)  Q4: 1.09 (0.86-1.39) | 0.63  0.88  0.49 | Unknown | 9 |
|  | SMHS | Q1: Ref  Q2: 0.84 (0.48-1.49)  Q3: 1.06 (0.61-1.84)  Q4: 0.76 (0.41-1.41) | 0.54 | Unknown | 9 |
| Carbohydrates | SWHS | Q1: Ref  Q2: 1.14 (0.89-1.45) Q3: 1.12 (0.87-1.43) Q4: 1.22 (0.95-1.55) | 0.30  0.38  0.11 | Unknown | 9 |
|  | SMHS | Q1: Ref  Q2: 1.02 (0.59-1.77)  Q3: 0.90 (0.50-1.60)  Q4: 0.83 (0.45-1.52) | 0.51 | Unknown | 9 |
| Total isoflavones | PLCO | <240: Ref  240-440: 1.30 (0.80-2.11)  440-810: 1.09 (0.65-1.82)  >810: 1.44 (0.85-2.44) | 0.29  0.76  0.18 | 48,549 | 9 |
|  | SWHS | Q1: Ref  **Q2: 0.52 (0.35-0.76)**  **Q3: 0.53 (0.36-0.78)**  **Q4: 0.47 (0.31-0.72)** | **0.001**  **0.001**  **0.0005** | 956 | 8 |
| Genistein | PLCO | <70: Ref  70-150: 1.18 (0.74-1.87)  150-360: 0.90 (0.54-1.48)  >360: 1.20 (0.73-1.98) | 0.49  0.70  0.48 | 48,549 | 9 |
|  | SWHS | Q1: Ref  **Q2:** **0.57 (0.39-0.83)**  **Q3: 0.49 (0.33-0.72)**  **Q4: 0.46 (0.30-0.70)** | **0.004**  **0.0004**  **0.0004** | 956 | 8 |
| Daidzein | PLCO | <100: Ref  100-180: 1.57 (0.97-2.53)  180-340: 1.02 (0.60-1.74)  **>340: 1.73 (1.03-2.90)** | 0.06  0.95  **0.04** | 48,549 | 9 |
|  | SWHS | Q1: Ref  **Q2: 0.57 (0.39-0.84)**  **Q3: 0.52 (0.36-0.77)**  **Q4: 0.50 (0.33-0.76)** | **0.004**  **0.0008**  **0.001** | 956 | 8 |
| Glycitein | PLCO | 0: Ref  <10: 0.84 (0.54-1.28)  10–20: 0.68 (0.33-1.43)  >20: 1.09 (0.66-1.78) | 0.44  0.31  0.75 | 48,549 | 9 |
|  | SWHS | Q1: 1.00  **Q2: 0.61 (0.42-0.89)**  **Q3: 0.54 (0.36-0.80)**  **Q4: 0.47 (0.31-0.72)** | **0.010**  **0.003**  **0.0005** | 956 | 8 |
| Fatty acid | SWHS | Q1: Ref  Q2: 0.90 (0.72-1.11)  Q3: 0.81 (0.64-1.02)  Q4: 0.92 (0.73-1.15)  Q5: 0.81 (0.64-1.03) | 0.35  0.08  0.48  0.08 | 65,076 | 9 |
| Vitamin K | JACC | Q1: Ref Q2: 1.08 (0.64-1.84) Q3: 0.87 (0.48-1.57) Q4: 0.79 (0.40-1.55) | 0.79  0.66  0.51 | Unknown | 9 |
| Saturated fatty acid | SWHS | Q1: Ref  Q2: 0.82 (0.66-1.02)  Q3: 0.83 (0.66-1.04)  Q4: 0.90 (0.72-1.13)  Q5: 0.86 (0.68-1.09) | 0.07  0.11  0.37  0.21 | 65,076 | 9 |
| Polyunsaturated fatty acid | SWHS | Q1: Ref  Q2: 0.82 (0.65-1.02)  Q3: 0.94 (0.76-1.18)  **Q4: 0.78 (0.62-0.99)**  Q5: 0.80 (0.64-1.01) | 0.08  0.59  **0.04**  0.05 | 65,076 | 9 |
| Linoleic acid | SWHS | Q1: Ref  Q2: 0.96 (0.77-1.20)  Q3: 0.91 (0.72-1.14)  Q4: 0.89 (0.71-1.12)  Q5: 0.81 (0.64-1.03) | 0.73  0.43  0.32  0.08 | 65,076 | 9 |
| Linolenic acid | SWHS | Q1: Ref  Q2: 0.88 (0.70-1.11)  Q3: 1.02 (0.81-1.27)  Q4: 0.93 (0.74-1.17)  Q5: 0.84 (0.67-1.07) | 0.28  0.87  0.55  0.14 | 65,076 | 9 |
| Arachidonic acid | SWHS | Q1: Ref Q2: 1.00 (0.81-1.24) Q3: 1.05 (0.85-1.31) Q4: 0.81 (0.63-1.03) Q5: 0.86 (0.68-1.10) | 0.99  0.67  0.09  0.22 | 65,076 | 9 |
| DHA | SWHS | Q1: Ref Q2: 1.02 (0.80-1.28) **Q3: 1.29 (1.03-1.62) Q4: 1.30 (1.03-1.64)** Q5: 1.21 (0.95-1.54) | 0.88  **0.03**  **0.03**  0.12 | 65,076 | 9 |
| Timnodonic acid-EPA | SWHS | Q1: Ref Q2: 0.92 (0.73-1.11) **Q3: 1.26 (1.01-1.57)** Q4: 1.25 (0.99-1.57) Q5: 1.10 (0.87-1.40) | 0.44  **0.04**  0.06  0.44 | 65,076 | 9 |
| Total n-3 PUFA | SWHS | Q1: Ref Q2: 0.97 (0.77-1.21) Q3: 0.94 (0.75-1.18) Q4: 0.97 (0.77-1.22) Q5: 0.91 (0.72-1.15) | 0.80  0.60  0.81  0.44 | 65,076 | 9 |
| Total n-6 PUFA | SWHS | Q1: Ref  Q2: 0.92 (0.73-1.15)  Q3: 0.89 (0.71-1.11)  Q4: 0.91 (0.73-1.14)  **Q5: 0.78 (0.62-0.99)** | 0.48  0.31  0.41  **0.04** | 65,076 | 9 |
| n-6 PUFA/n-3 PUFA ratio | SWHS | Q1: Ref  Q2: 0.98 (0.79-1.23)  Q3: 0.79 (0.62-1.00)  Q4: 0.82 (0.65-1.04)  **Q5: 0.74 (0.59-0.93)** | 0.87  0.05  0.10  **0.009** | 65,076 | 9 |
| Total n-3 HUFA | SWHS | Q1: Ref Q2: 0.92 (0.73-1.16) Q3: 1.24 (0.99-1.55) **Q4: 1.26 (1.00-1.58)** Q5: 1.17 (0.92-1.48) | 0.49  0.06  **0.047**  0.20 | 65,076 | 9 |
| Mono-unsaturated fatty acid | SWHS | Q1: Ref  Q2: 1.02 (0.82-1.27) Q3: 0.91 (0.73-1.15) Q4: 0.98 (0.78-1.24) Q5: 0.90 (0.71-1.15) | 0.87  0.42  0.87  0.40 | 65,076 | 9 |
| PUFA + MUFA/SFA | GCS | <0.6: Ref  0.6-0.8: 1.02 (0.53-1.96) >0.8: 1.45 (0.78-2.70) | 0.96  0.24 | Unknown | 9 |
| Phytoestrogens | PLCO | >1030: 1.42 (0.83-2.41)  510-1030: 1.16 (0.69-1.95)  290-510: 1.32 (0.81-2.14)  <290: Ref | 0.27  0.59  0.20 | 48,549 | 9 |
| Biochanin A | PLCO | <10: Ref 10-20: 1.20 (0.75-1.92) 20-40: 1.31 (0.81-2.12)  >40: 1.62 (0.97-2.69) | 0.46  0.27  0.06 | 48,549 | 9 |
| Formononetin | PLCO | <10: Ref  10-20: 0.89 (0.51-1.55) 20-50: 1.10 (0.67-1.81)  >50: 1.45 (0.96-2.17) | 0.42  0.70  0.07 | 48,549 | 9 |
| Coumestrol | PLCO | <30: Ref  30-80: 1.19 (0.75-1.91)  80-170: 1.15 (0.70-1.89)  >170: 1.42 (0.87-2.31) | 0.48  0.59  0.16 | 48,549 | 9 |

^%^Odds ratio

*Laboratory tests*

| **Factor** | **Study name** | **Hazard ratio (95%CI)** | **p-value** | **Total number of never-smokers** | **Total risk of bias score** |
| --- | --- | --- | --- | --- | --- |
| **Blood tests** | | | | | |
| Serum testosterone | HIMS | 1.10 (0.60-2.00) | 0.76 | 1,226 | 7 |
|  | SWHS | Q1: Ref **Q2: 0.60 (0.41-0.87)** Q3: 0.80 (0.52-1.24) Q4 0.63 (0.40-1.00)^%^ | **0.007**  0.32  0.05 | 794 | 8 |
| C-reactive protein (CRP) | UCC-SMART | 1.16 (0.88-1.53) | 0.30 | 1,587 | 7 |
|  | SWHS | Q1: Ref  Q2: 0.76 (0.47-1.25)  **Q3:** **0.57 (0.34-0.97)**  Q4: 0.63 (0.37-1.06)^%^ | 0.27  **0.04**  0.08 | 511 | 9 |
| Bilirubin | UK Biobank | 1.00 (0.87-1.15)^**^ | 0.97 | 194,558 | 6 |
|  | KCPS-II | Total per 1SD increase:  1.11 (0.98-1.25)  Indirect: 1.09 (0.94-1.26)  Direct: 1.01 (0.86-1.19) | 0.09  0.25  0.91 | 63,880 | 9 |
| White cell count | UK Biobank | Women  ≤5.55: Ref  5.55-6.51: 1.27 (0.76-2.13)  6.51-7.67: 1.09 (0.64-1.88)  **>7.67: 1.82 (1.10-3.00)**  Men  ≤5.50: Ref 5.50-6.41: 0.77 (0.41-1.46) 6.41-7.50: 0.63 (0.32-1.22) >7.50: 0.96 (0.53-1.75) | 0.37  0.75  **0.02**  0.42  0.17  0.90 | 232,528 | 9 |
| Neutrophil-to-lymphocyte ratio | UK Biobank | Women: **1.20 (1.01-1.43)**  Men: 1.06 (0.85-1.33) | **0.03**  0.62 | 232,528 | 9 |
| Platelet count | UK Biobank | Women: **1.18 (1.04-1.34)**  Men: 1.13 (0.95-1.34) | **0.01**  0.18 | 222,893 | 8 |
| Mean platelet volume | UK Biobank | 1.01 (0.85-1.19) | 0.94 | 222,893 | 8 |
| Platelet distribution width | UK Biobank | 1.07 (0.91-1.26) | 0.40 | 222,893 | 8 |
| eGFR | MJ Health Database | ≥90: Ref  **60-89: 1.58 (1.25-2.00)**  **45-59: 1.85 (1.23-2.79)**  <45: 1.47 (0.54-4.06)  Annual decline ≥ 5: 1.28 (0.94-1.73) | **<0.001**  **0.003**  0.45  0.12 | 130,559 | 7 |
| Total cholesterol | Kailuan | **Q1: 1.42 (1.05-1.93)** Q2: Ref  **Q3: 1.46 (1.07-2.00)** Q4: 1.24 (0.90-1.70) **Q5: 1.38 (1.01-1.89)** | **0.02**  **0.02**  0.19  **0.04** | 56,097 | 7 |
| Triglycerides | Kailuan | Q1: 1.34 (0.99-1.82)  Q2: Ref  Q3: 1.20 (0.89-1.63)  Q4: 0.98 (0.70-1.36)  **Q5 1.37 (1.04-1.82)** | 0.06  0.24  0.91  **0.03** | 56,097 | 7 |
| LDL-C | Kailuan | **Q1: 1.39 (1.02-1.89)** Q2: Ref  Q3: 1.17 (0.83-1.65) **Q4: 1.58 (1.14-2.20) Q5: 1.42 (1.00-2.02)** | **0.04**  0.38  **0.006**  0.05 | 56,097 | 7 |
| HDL-C | Kailuan | Q1: 0.90 (0.64-1.25) Q2: Ref  Q3: 0.73 (0.52-1.02) Q4: 0.80 (0.59-1.09) Q5: 0.89 (0.66-1.20) | 0.55  0.07  0.15  0.45 | 56,097 | 7 |
| Non-HDL-C | Kailuan | Q1: 0.98 (0.72-1.34) Q2: Ref  Q3: 1.18 (0.87-1.59) Q4: 1.15 (0.86-1.55) Q5: 1.03 (0.76-1.39) | 0.91  0.29  0.36  0.86 | 56,097 | 7 |
| Fasting blood sugar | NHIS | With T2DM Hypoglycaemic:  0.76 (0.36-1.59) Normal: Ref  100-125: 1.01 (0.95-1.09) ≥126 mg/dL: 1.05 (0.94-1.18)  Without T2DM  Hypoglycaemic:  1.40 (0.58-3.38)  Normal: Ref  100-125: 0.99 (0.89-1.10)  ≥126: 1.09 (0.89-1.33) | 0.48  0.79  0.41  0.46  0.86  0.41 | 300,518 | 9 |
| Folate | CHHRS | <6.72ng/mL:  **1.72 (1.11-2.66)^%^** | **0.01** | 555 | 8 |
| 5-mTHF | CHHRS | <5.80 ng/mL:  **1.58 (1.02-2.43)^%^** | **0.04** | 555 | 8 |
| Vitamin D | WHI | <33.9 nmol/L: Ref 33.9-45.3: 1.11 (0.65-1.88) 45.3-58.9: 1.15 (0.69-1.90)  ≥58.9: 1.06 (0.61-1.84)^%^ | 0.71  0.60  0.85 | 596 | 7 |
| CEA | MJ Health Database | **1.06 (1.04-1.08)** | **<0.001** | 130,559 | 7 |
| Estrone | SWHS | Q1: Ref  Q2: 0.83 (0.55-1.27) Q3: 0.93 (0.61-1.41) Q4: 0.74 (0.46-1.20)^%^ | 0.40  0.72  0.22 | 794 | 8 |
| Estradiol | SWHS | Q1: Ref  Q2: 0.96 (0.63-1.44) Q3: 0.95 (0.61-1.47) Q4: 0.66 (0.38-1.14)^%^ | 0.83  0.81  0.14 | 794 | 8 |
| Free estradiol | SWHS | Q1: Ref  Q2: 0.90 (0.56-1.43)  Q3: 0.93 (0.58-1.49)  Q4: 0.91 (0.55-1.51) | 0.65  0.77  0.72 | 794 | 8 |
| Estrone/  androstenedione ratio | SWHS | Q1: Ref  Q2: 0.85 (0.55-1.29)  Q3: 1.01 (0.66-1.55)  **Q4: 0.59 (0.35-0.97)** | 0.43  0.96  **0.04** | 794 | 8 |
| Androstenedione | SWHS | Q1: Ref  Q2: 0.81 (0.52-1.25) Q3: 0.73 (0.47-1.15) Q4: 0.86 (0.55-1.36)^%^ | 0.33  0.17  0.52 | 794 | 8 |
| SHBG | SWHS | Q1: Ref Q2: 1.07 (0.66-1.72) Q3: 1.33 (0.84-2.11) Q4: 1.21 (0.74-1.99)^%^ | 0.80  0.22  0.45 | 794 | 8 |
| Free testosterone | SWHS | Q1: Ref Q2: 0.67 (0.44-1.04) Q3: 0.82 (0.53-1.27) **Q4: 0.63 (0.39-0.99)**^%^ | 0.07  0.37  **0.045** | 794 | 8 |
| sIL-6R | SWHS | Q1: Ref  Q2: 1.20 (0.69-2.10)  **Q3: 1.91 (1.12-3.23)  Q4: 2.37 (1.40-4.02)^%^** | 0.54  **0.02**  **0.001** | 511 | 9 |
| sTNFRI | SWHS | Q1: Ref  Q2: 0.68 (0.42-1.11)  Q3: 0.88 (0.54-1.41)  **Q4: 0.49 (0.29-0.83)^%^** | 0.12  0.61  **0.008** | 511 | 9 |
| sVEGFR2 | SWHS | Q1: Ref Q2: 0.63 (0.39-1.04)  Q3: 0.88 (0.54-1.42)  **Q4: 0.45 (0.26-0.76)^%^** | 0.06  0.62  **0.004** | 511 | 9 |
| sVEGFR3 | SWHS | Q1: Ref  Q2: 0.78 (0.48-1.26)  Q3: 0.80 (0.49-1.30)  **Q4: 0.53 (0.32-0.90)^%^** | 0.32  0.38  **0.02** | 511 | 9 |
| IL-21 | SWHS | Q1: Ref  Q2: 0.99 (0.59-1.64)  **Q3: 0.51 (0.29-0.89)  Q4: 0.53 (0.31-0.93)^%^** | 0.97  **0.02**  **0.02** | 511 | 9 |
| IL-10 | SWHS | Q1: Ref  Q2: 0.85 (0.50-1.45)  Q3: 0.64 (0.37-1.11)  Q4: 0.60 (0.34-1.05)^%^ | 0.56  0.11  0.08 | 511 | 9 |
| CX3CL1/ Fractalkine | SWHS | Q1: Ref  Q2: 1.10 (0.65-1.84)  Q3: 0.57 (0.33-1.01)  **Q4: 0.54 (0.30-0.96)^%^** | 0.73  0.05  **0.04** | 511 | 9 |
| CCL2/MCP-1 | SWHS | Q1: Ref  Q2: 1.05 (0.61-1.78)  Q3: 1.65 (0.97-2.81)  Q4: 1.62 (0.94-2.80)^%^ | 0.87  0.06  0.08 | 511 | 9 |
| Sphingomyelin | CPS | 1.07 (0.83-1.36)^%^ | 0.61 | 462 | 9 |
| Taurodeoxycholic acid 3-sulfate | CPS | 1.09 (0.87-1.38)^%^ | 0.45 | 462 | 9 |
| CYPA26 activity | UK Biobank | 1.04 (0.95-1.14)^%^ | 0.35 | 214,592 | 7 |
| Allostatic load (physical measurements and blood tests) | UK Biobank | High: 0.93 (0.74-1.17) | 0.55 | 188,742 | 8 |
| **Genetic information** | | | | | |
| Leukocyte telomere length | Singapore Chinese Health Study | Adenocarcinoma Q1 (shortest): Ref Q2: 1.22 (0.65-2.29) Q3: 1.53 (0.84-2.81) Q4: 1.39 (0.74-2.60) **Q5: 3.14 (1.80-5.49)**  Squamous cell carcinoma  Q1: Ref  Q2: 0.31 (0.03-3.02) Q3: 0.30 (0.03-2.91) Q4: 0.87 (0.17-4.49) Q5: 0.56 (0.09-3.58) | 0.55  0.17  0.31  **<0.001**  0.32  0.31  0.88  0.55 | 18,034 | 8 |
|  | SMHS | Q1 (shortest): Ref  Q2: 1.37 (0.71-2.62)  **Q3: 3.48 (1.85-6.57)**  Q4: 1.85 (0.99-3.44)^%^ | 0.35  **0.0001**  0.05 | 323 | 8 |
|  | SWHS | Q1 (shortest): Ref  **Q2: 1.45 (1.05-2.02)**  **Q3: 1.76 (1.28-2.43)**  **Q4: 2.10 (1.52-2.90)** | **0.03**  **0.0005**  **<0.0001** | 1590 | 8 |
|  | UK Biobank | Per IQR change:  **1.45 (1.23-1.71)** | **<0.001** | 234,302 | 8 |
| Mosaic loss of chromosome Y | UK Biobank | Non-expanded: 0.90 (0.67-1.22)  Expanded: 0.97 (0.62-1.52) | 0.50  0.90 | 106,714 | 8 |
|  | CKB | Non-expanded: 1.01 (0.45-2.30)  Expanded: 1.72 (0.75-3.92) | 0.98  0.20 | 10,678 | 8 |
| Clonal haematopoeisis | UK Biobank | 1.64 (0.79-3.39)^%^ | 0.18 | 725 | 8 |
|  | Mass General Brigham Biobank | 1.50 (0.86-2.62)^%^ | 0.15 | 548 | 8 |
| Lung cancer-related CpG sites | NOWAC | Various, including cg10151248-PC  **0.36 (0.17-0.77)^%^** and cg13482620-B3GNTL1 **0.31 (0.14-0.67)^%^** | **Various** | 71 | 7 |
| SNP UGT1A1 rs887829 | UK Biobank | TT: Ref  CC or CT: 1.1 (0.7-1.6)** | 0.82 | 194,558 | 6 |
| 8-*epi*-prostaglandin F_2α_ | SMHS | Q1: Ref  Q2: 1.62 (0.70-3.78)  Q3: 1.54 (0.68-3.52)  Q4: 0.71 (0.29-1.75)^%^ | 0.27  0.31  0.46 | 232 | 8 |
| DNA adducts in buccal swab | SMHS | Various including 8-oxo-dGuo | All non-significant |  | 7 |
| **Urinary tests** | | | | | |
| Urine metabolamics | SWHS | pos_2.61_127.0382m/z  **0.57 (0.46-0.72)**^%^  neg_2.60_369.0408m/z  **0.97 (0.96-0.98)^%^**  pos_2.61_184.0325n  **0.55 (0.43-0.71)^%^**  5-methyl2-furoic acid  Tertile 1: Ref  **T2: 0.52 (0.34-0.80)**  **T3: 0.46 (0.30-0.70)^%^** | **<0.001**  **<0.001**  **<0.001**  **0.003**  **<0.001** | 564 | 9 |
| Urinary phytoestrogens | SWHS | Isoflavones  Q1: Ref  **Q2: 0.57 (0.39-0.83)**  **Q3: 0.64 (0.44-0.92)**  **Q4: 0.60 (0.41-0.86)^%^**  Daidzein  Q1: Ref  Q2: 0.54 (0.36-0.80)  Q3: 0.73 (0.51-1.05)  Q4: 0.56 (0.38-0.82)^%^  Genistein  Q1: Ref  **Q2: 0.62 (0.43-0.90)**  Q3: 0.79 (0.55-1.13)  **Q4: 0.63 (0.44-0.91)^%^**  Equol  Q1: Ref  Q2: 1.42 (0.99-2.04)  Q3: 0.91 (0.60-1.36)  Q4: 0.95 (0.64-1.41)^%^  O-Desmethylangolensin  Q1: Ref  Q2: 0.81 (0.57-1.17)  **Q3: 0.60 (0.40- 0.88)**  Q4: 0.94 (0.65-1.36)^%^  Enterodiol  Q1: Ref  Q2: 0.89 (0.62-1.29)  Q3: 0.80 (0.54-1.18)  Q3: 0.81 (0.56-1.17)^%^  Enterolactone  Q1: Ref  Q2: 0.97 (0.67-1.41)  Q3: 1.12 (0.77-1.63)  Q4: 0.94 (0.64-1.37)^%^ | 0.04  0.05  0.06  0.24  0.85  0.15  0.41 | 956 | 8 |
| Urinary benzothiazole, benzotriazole and derivatives | QEEHH | Benzothiazole  Q1: Ref  Q2: 1.17 (0.51-2.65)  Q3: 2.41 (1.15-5.03)  Q4: 0.65 (0.26-1.57)^%^  2-OH-BTH  **2.44 (1.29-4.62)**^%^  BTR  0.60 (0.33-1.09)^%^  1-OH-BTR  Q1: Ref  Q2: 1.20 (0.55-2.61)  Q3: 1.31 (0.60-2.84)  Q4: 1.07 (0.48-2.37)^%^  XTR  Q1: Ref  Q2: 1.73 (0.75-3.96)  Q3: 2.06 (0.93-4.55)  Q4: 1.65 (0.74-3.69)^%^  TTR  Q1: Ref  Q2: 0.84 (0.37-1.88)  Q3: 1.38 (0.65-2.95  Q4: 1.07 (0.49-2.31)^%^  2-MeS-BTH  Q1: Ref  Q2: 0.68 (0.32-1.45)  Q3: 0.62 (0.28-1.34)  Q4: 0.78 (0.37-1.68)^%^  2-NH_2_-BTH  1.27 (0.72-2.23)^%^ | 0.88  **<0.01**  0.09  0.98  0.38  0.66  0.48  0.39 | 228 | 8 |

^%^Odds ratio **Incidence rate ratio

# Appendix 6: Factors associated with medication, dietary intake and urine laboratory tests

*Medication*

Angiotensin-converting enzyme (ACE)-inhibitors, angiotensin receptor blockers (ARB) and calcium channel blockers (CCB) were not associated with LCINS (n=132,534)^1^ in a study with moderate risk of bias, however participants using ARB were less likely to have LCINS than those using CCB at median follow-up of 7.8 years (aHR 0.64 (95%CI 0.42-0.99), n=39,784)^2^ in a study with low risk of bias.

In single studies with low risk of bias, use of oral bisphosphonate was negatively associated with LCINS (aHR 0.57 (95%CI 0.39-0.84), n=75,962)^3^. In a study reviewing the use of aspirin, statin and metformin individually and in combination, diabetics who were prescribed metformin were less likely to have LCINS than non-diabetics (aHR 0.86 (95%CI 0.74-0.99)) but not when compared with diabetics who had never used metformin (aHR 0.87 (95%CI 0.71-1.07), n=515,100)^4^. Furthermore, individuals who were prescribed aspirin, statin and metformin in combination were less likely to be diagnosed with LCINS (aHR 0.38 (0.20-0.73), n=515,100)^4^.

*Dietary intake*

Dietary indices

Meta-analysis of two studies with very low risk of bias reviewing glycaemic index found no association with LCINS (n=Unknown, Appendix 4).^5^

Three studies reviewing HEI-2015 (n=at least 122,585) found no association with LCINS^6–8^; a study using a previous HEI version (HEI-10) found lower quality diets were associated with LCINS (lowest quality aHR 2.70 (95%CI 1.25-5.83), n=25,860)^9^. Two of three studies reviewing AHEI-2010 suggested higher scores, associated with healthier diets, were associated with lower risk of LCINS (n=at least 122,585, Appendix 5)^6–8.^

There were no consistent associations between LCINS and any other dietary index reviewed in multiple studies (AMED, DASH) or single studies (DII, glycaemia index and MINDDiet) (Appendix 5).

All studies reviewing dietary indices had low risk of bias.

Specific foods

Six factors were reported in multiple studies. Meta-analysis of three of four studies with low-moderate risk of bias reporting fruit intake (n=at least 163,488)^1,8,10^ and two studies reporting offal intake with low risk of bias (n=195,643) found no association with LCINS^11^ (Appendix 4).

None of the factors reported in single studies were consistently associated with LCINS (Appendix 5).

Specific nutrients

Nine factors were reported in multiple studies. Meta-analysis was possible for five factors (retinol, fibre, vitamin C, glycaemic load and carbohydrates); none were associated with LCINS (Appendix 4).^5,11^ There was no consistent association between LCINS and any other factor reported in multiple or single studies (Appendix 5). All studies reporting on specific nutrients had low risk of bias.

*Laboratory tests*

Urine tests

Urine metabolamics such as pos_2.61_127.0382m/z (which may be related to soy intake) and 5-methyl2-furoic acid (n=564)^12^ and certain urinary phytoestrogens such as isoflavones (n=956)^13^ were negatively associated with LCINS (Table 2). Of various measurements of urinary benzothiazole, benzotriazole and its derivatives, which may reflect exposure to environment pollutants, only 2-OH-BTH was associated with LCINS (aOR 2.44 (95%CI 1.29-4.62), n=228)^14^.

All studies reporting on urine tests had low risk of bias.

# Appendix 7: Subgroup analyses

*Subgroup analyses by region (Western and East Asian)*

Of 32 meta-analyses conducted, seven included studies only conducted in East Asia and six only in Western countries. Two meta-analyses included only Western and Iranian studies and one only East Asian and Iranian studies. Of the remaining 16 meta-analyses, five only included two studies and were not suitable for subgroup analyses. Subgroup analysis by region was performed for the remaining 11 meta-analyses.

| **Factor** | **Region** | **Adjusted hazard ratio (95% CI)** | **Number of studies** | **Hetp** | ***I^2^* (%)** | **p-value for subgroup differences** |
| --- | --- | --- | --- | --- | --- | --- |
| **Female sex** | Western | 0.75 (0.48-1.18) | 3 | 0.403 | 0.0 | 0.710 |
|  | East Asian | 0.79 (0.28-2.22) | 2 | 0.379 | 0.0 |  |
| **Hormone replacement therapy** | Western | 0.89 (0.72-1.11) | 2 | 0.752 | 0.0 | 0.198 |
|  | East Asian | 1.03 (0.98-1.08) | 1 |  |  |  |
| **COPD/ obstructive spirometry** | Western | 1.25 (0.03-53.31) | 2 | 0.297 | 7.9 | 0.459 |
|  | East Asian | 1.66 (0.57-4.78) | 3 | <0.0001 | 92.2 |  |
| **Rheumatoid arthritis** | Western | 1.64 (1.22-2.21) | 2 | 0.854 | 0.0 | 0.187 |
|  | East Asian | 1.31 (1.13-1.52) | 1 |  |  |  |
| **Cancer** | Western | 1.40 (0.87-2.25) | 1 |  |  | 0.117 |
|  | East Asian | 2.05 (1.96-2.14) | 2 | 0.658 | 0.0 |  |
| **Diabetes** | Western | 0.87 (0.52-1.46) | 1 | 0.008 | 79.4 | 0.491 |
|  | East Asian | 1.07 (0.83-1.37) | 3 |  |  |  |
| **Peridontitis** | Western | 1.11 (0.61-2.03) | 2 | 0.167 | 47.6 | **0.001** |
|  | East Asian | **3.56 (2.68-4.72)** | 1 |  |  |  |
| **Family history lung cancer** | Western | 0.86 (0.35-2.11) | 3 | 0.120 | 52.8 | **0.006** |
|  | East Asian | **1.56 (1.23-1.98)** | 3 | 0.517 | 0.0 |  |
| **Lower educational attainment** | Western | 1.14 (0.79-1.66) | 1 |  |  | 0.814 |
|  | East Asian | 1.20 (0.98-1.47) | 2 | 0.067 | 70.3 |  |
| **Passive smoking** | Western | 1.28 (0.93-1.76) | 4 | 0.282 | 21.4 | 0.829 |
|  | East Asian | 1.31 (1.14-1.50) | 2 | 0.720 | 0.0 |  |
| **PM2.5** | Western | 1.17 (0.94-1.46) | 4 | 0.819 | 0.0 | 0.950 |
|  | East Asian | 1.16 (0.77-1.74) | 3 | 0.068 | 62.9 |  |

*Meta-regression by mean age of study cohort*

20 of 32 meta-analyses included only two studies for which mean age was available and therefore were not suitable for meta-regression. Meta-regression was performed for the remaining 12 factors. For two meta-analyses, mean age was not available for one of the studies and this study was excluded from meta-regression.

| **Factor** | **Number of studies** | **p-value for impact of age** |
| --- | --- | --- |
| Sex | 6 (out of 6) | 0.409 |
| Hormone replacement therapy | 3 (out of 3) | 0.423 |
| Breastfeeding | 3 (out of 3) | 0.538 |
| COPD/obstructive spirometry | 4 (out of 5) | 0.640 |
| Hypertension | 3 (out of 3) | 0.825 |
| Diabetes | 4 (out of 4) | 0.746 |
| Family history lung cancer | 6 (out of 6) | 0.214 |
| Lower educational attainment | 4 (out of 4) | 0.823 |
| Passive smoking | 6 (out of 6) | 0.220 |
| PM2.5 | 6 (out of 7) | 0.119 |
| Urban residence | 3 (out of 3) | 0.119 |
| Fruit intake | 3 (out of 3) | 0.981 |

# Appendix 8: Summary of risk of bias

Criteria

Note: A study can be awarded a maximum of one star for each numbered item within the Selection and Exposure categories. A maximum of two stars can be given for Comparability.

**CASE CONTROL STUDIES**

**Selection**

1) Is the case definition (how they define people with lung cancer) adequate?

a) yes, with independent validation *****

b) yes, eg record linkage or based on self reports

c) no description

2) Representativeness of the cases

a) consecutive or obviously representative series of cases *****

b) potential for selection biases or not stated

3) Selection of controls (i.e. people without lung cancer)

a) community controls *****

b) hospital controls

c) no description

4) Definition of Controls

a) no history of disease (endpoint) *****

b) no description of source

**Comparability**

1) Comparability of cases and controls on the basis of the design or analysis

a) study controls for age and sex *****

b) study controls for any additional factor *****

**Exposure**

1) Ascertainment of exposure

a) secure record (eg surgical records) *****

b) structured interview or questionnaire where blind to case/control status *****

c) interview not blinded to case/control status

d) written self report or medical record only

e) no description

2) Same method of ascertainment for cases and controls

a) yes *****

b) no

3) Non-Response rate

a) same rate for both groups *****

b) non respondents described

c) rate different and no designation

**COHORT STUDIES**

**Selection**

1) Representativeness of the exposed cohort

a) truly representative of the average never-smoker in the community *****

b) somewhat representative of the average never-smoker in the community *****

c) selected group of users e.g. nurses, volunteers

d) no description of the derivation of the cohort

2) Selection of the non exposed cohort

a) drawn from the same community as the exposed cohort *****

b) drawn from a different source

c) no description of the derivation of the non exposed cohort

3) Ascertainment of exposure

a) secure record (eg surgical records) *****

b) structured interview or validated questionnaire *****

c) written self report

d) no description

4) Demonstration that outcome of interest was not present at start of study

a) yes *****

b) no

**Comparability**

1) Comparability of cohorts on the basis of the design or analysis (covariates)

a) study controls for age and sex *****

b) study controls for additional relevant factor *****

**Outcome**

1) Assessment of outcome (lung cancer)

a) independent blind assessment *****

b) record linkage *****

c) self report

d) no description

2) Was follow-up long enough for outcomes to occur

a) yes (5 years) *****

b) no

3) Adequacy of follow up of cohorts

a) complete follow up - all subjects accounted for *****

b) subjects lost to follow up unlikely to introduce bias - small number lost <10 % or description provided of those lost) *****

c) follow up rate (number lost >10%) and no description of those lost

d) no statement

| **Report first author** | **Year** | **Selection** | **Compa-rability** | **Exposure (case-control)**  **Outcome (cohort)** | **Total score** |
| --- | --- | --- | --- | --- | --- |
| Cheng E.S. | 2022 | 3 | 2 | 1 | 6 |
| Fraser G.E. | 2020 | 3 | 2 | 2 | 7 |
| Bonner M. | 2017 | 2 | 2 | 3 | 7 |
| Gharibvand L. | 2017 | 3 | 1 | 3 | 7 |
| Gallet J. | 2022 | 3 | 2 | 3 | 8 |
| Michaud D.S. | 2018 | 4 | 2 | 2 | 8 |
| Nohara S. | 2025 | 4 | 2 | 2 | 8 |
| Erhunmwunsee L. | 2022 | 1 | 2 | 2 | 5 |
| Nomura S.J.O. | 2018 | 4 | 2 | 3 | 9 |
| Bethea T.N. | 2022 | 2 | 2 | 3 | 7 |
| Murphy R.A. | 2022 | 4 | 2 | 1 | 7 |
| Wang F. | 2021 | 2 | 2 | 2 | 6 |
| Guo L. | 2023 | 4 | 2 | 1 | 7 |
| Wu Z. (CanSPUC) | 2022 | 3 | 1 | 1 | 5 |
| Yang Z. | 2021 | 2 | 2 | 1 | 5 |
| Wei Y. | 2022 | 4 | 2 | 2 | 8 |
| Li X. | 2019 | 3 | 1 | 3 | 7 |
| Jian S. | 2022 | 4 | 2 | 2 | 8 |
| Zhu M. | 2024 | 4 | 2 | 3 | 9 |
| Ma Z. | 2023 | 4 | 2 | 2 | 8 |
| Weng C. | 2024 | 4 | 2 | 2 | 8 |
| Patel A.V. | 2017 | 4 | 2 | 3 | 9 |
| Tang Z. | 2024 | 4 | 2 | 3 | 9 |
| Heath A.K. (EPIC) | 2022 | 3 | 2 | 2 | 7 |
| Wang Q. (GCS) | 2021 | 4 | 2 | 3 | 9 |
| Yano Y. | 2024 | 4 | 2 | 3 | 9 |
| Chan Y.X. | 2017 | 3 | 2 | 2 | 7 |
| Yan Fangyu | 2022 | 4 | 2 | 3 | 9 |
| Mori N. | 2017 | 4 | 1 | 2 | 7 |
| Abe S.K. | 2021 | 3 | 2 | 3 | 8 |
| Wilunda C. | 2021 | 2 | 2 | 2 | 6 |
| Lyu Z. | 2019 | 3 | 2 | 2 | 7 |
| Wu Z. (Kailuan) | 2022 | 3 | 2 | 2 | 7 |
| Lee Y-G | 2025 | 3 | 2 | 2 | 7 |
| Shin J.W. | 2025 | 4 | 2 | 3 | 9 |
| Ruan G-T | 2023 | 4 | 2 | 2 | 8 |
| Cahoon E.K. | 2017 | 3 | 1 | 3 | 7 |
| Du Y. | 2022 | 4 | 2 | 2 | 8 |
| Tian R. (MGBB) | 2022 | 4 | 2 | 2 | 8 |
| Mugikura M. | 2020 | 4 | 2 | 3 | 9 |
| Huang H-L | 2021 | 4 | 2 | 3 | 7 |
| Park S.Y. | 2021 | 4 | 2 | 3 | 9 |
| Choi E. | 2023 | 4 | 1 | 2 | 7 |
| Cho M.H | 2023 | 4 | 2 | 2 | 8 |
| Park H.Y. | 2020 | 3 | 2 | 2 | 7 |
| Park H.Y. | 2022 | 3 | 2 | 2 | 7 |
| Choi H. | 2022 | 4 | 2 | 3 | 9 |
| Moon S. | 2020 | 4 | 2 | 3 | 9 |
| Kang J. | 2021 | 4 | 2 | 3 | 9 |
| Choi Y.J. | 2019 | 4 | 2 | 1 | 7 |
| Jeon K.H | 2020 | 2 | 2 | 2 | 6 |
| Park H.J. | 2019 | 4 | 2 | 3 | 9 |
| Yang S. | 2021 | 4 | 2 | 3 | 9 |
| Lee H.W. | 2022 | 4 | 2 | 2 | 8 |
| Ko Y.H. | 2020 | 4 | 2 | 3 | 9 |
| Kim B-G | 2024 | 4 | 2 | 2 | 8 |
| Heath A.K. (NLCS) | 2022 | 4 | 2 | 3 | 9 |
| Schulpen M. | 2018 | 4 | 2 | 3 | 9 |
| Korn A.R. | 2022 | 4 | 2 | 2 | 8 |
| Huang T. | 2021 | 2 | 2 | 2 | 6 |
| Trudel-Fitzgerald C. | 2022 | 3 | 2 | 2 | 7 |
| Sandanger T.M. | 2018 | 4 | 2 | 1 | 7 |
| Hansen M.S. | 2021 | 3 | 2 | 2 | 7 |
| Borch K.B. | 2019 | 2 | 1 | 2 | 5 |
| Wang Q. (PLCO) | 2021 | 4 | 2 | 3 | 9 |
| Abdel-Rahman O. | 2020 | 3 | 2 | 2 | 7 |
| Titan A.L. | 2020 | 2 | 2 | 2 | 6 |
| Zhu Z. | 2023 | 4 | 2 | 1 | 7 |
| Gai X. | 2024 | 3 | 2 | 1 | 6 |
| Mao W. | 2024 | 4 | 2 | 2 | 8 |
| Yuan J. (SMHS) | 2018 | 4 | 2 | 2 | 8 |
| Hosgood H.D. (SMHS) | 2021 | 3 | 2 | 3 | 8 |
| Wong J.Y.Y. (SMHS) | 2024 | 4 | 2 | 2 | 8 |
| Hosgood H.D. (SWHS) | 2021 | 3 | 2 | 3 | 8 |
| Shiels M.S. | 2017 | 4 | 2 | 3 | 9 |
| Sun J. | 2018 | 4 | 2 | 3 | 9 |
| Seow W.J. | 2019 | 4 | 2 | 3 | 9 |
| Luu H.N. | 2018 | 4 | 2 | 3 | 9 |
| Zhao Y. | 2022 | 4 | 2 | 2 | 8 |
| Li M. | 2022 | 4 | 2 | 2 | 8 |
| Wong J.Y.Y. (SWHS) | 2024 | 4 | 2 | 2 | 8 |
| Recalde M. | 2021 | 4 | 2 | 2 | 8 |
| Turkiewicz A. | 2022 | 4 | 2 | 2 | 8 |
| Yuan J. (Singapore Chinese Health Study) | 2018 | 3 | 2 | 3 | 8 |
| Mehta S.S. | 2023 | 3 | 2 | 2 | 7 |
| Munro H.M. | 2023 | 3 | 2 | 3 | 8 |
| Rait G. | 2020 | 3 | 2 | 2 | 7 |
| Van't Klooster C.C. | 2019 | 3 | 2 | 2 | 7 |
| Zhang P. | 2022 | 4 | 2 | 2 | 8 |
| He H. | 2022 | 3 | 2 | 3 | 8 |
| Warkentin M.T. | 2019 | 4 | 2 | 3 | 9 |
| Wong J.Y.Y. (UK Biobank) | 2021 | 4 | 2 | 3 | 9 |
| Horsfall L.J. | 2020 | 3 | 2 | 1 | 6 |
| Christakoudi S. | 2023 | 3 | 2 | 3 | 8 |
| Huang Y. | 2021 | 4 | 2 | 3 | 9 |
| Zhang J. | 2023 | 4 | 2 | 2 | 8 |
| Han D. | 2023 | 4 | 2 | 2 | 8 |
| Zhang S. | 2024 | 4 | 2 | 2 | 8 |
| Weng C. (UK Biobank) | 2024 | 4 | 2 | 2 | 8 |
| Giratallah H. | 2023 | 3 | 2 | 2 | 7 |
| Guan Y. | 2024 | 4 | 2 | 2 | 8 |
| Liao Y. | 2024 | 3 | 2 | 3 | 8 |
| Fan Y. | 2024 | 4 | 2 | 2 | 8 |
| Tian R. (UK Biobank) | 2022 | 4 | 2 | 2 | 8 |
| Brooks R.T. | 2024 | 3 | 2 | 2 | 7 |
| Myneni A.A. | 2021 | 3 | 1 | 3 | 7 |
| Gowda S.N. | 2019 | 3 | 2 | 3 | 8 |
| Tao M.H. | 2018 | 3 | 2 | 2 | 7 |
| Nwizu N.N. | 2017 | 2 | 2 | 2 | 6 |
| Cheng T.-Y.D. | 2017 | 3 | 1 | 3 | 7 |
| Tao M.H. | 2017 | 4 | 2 | 2 | 8 |
| Cannioto R. | 2018 | 3 | 2 | 2 | 7 |
| Chatzidionysiou K. | 2022 | 4 | 2 | 2 | 8 |
| Delcoigne B. | 2017 | 4 | 2 | 3 | 9 |
| Derouen M.C. | 2022 | 3 | 2 | 1 | 6 |
| DiMarzio P. | 2018 | 3 | 2 | 1 | 6 |
| Kelly-Reif K. | 2022 | 2 | 2 | 2 | 6 |
| Kim Y.W. | 2025 | 4 | 2 | 2 | 8 |
| Kyaw T.W. | 2024 | 4 | 2 | 2 | 8 |
| Li J. | 2020 | 3 | 2 | 2 | 7 |
| Yoon J.H. | 2018 | 2 | 2 | 2 | 6 |

# References for appendices

1. Cheng ES, Weber MF, Steinberg J, Canfell K, Yu XQ. Evaluating risk factors for lung cancer among never-smoking individuals using two Australian studies. *J Cancer Res Clin Oncol*. 2022;148(10):2827-2840. doi:10.1007/s00432-022-04043-9

2. Moon S, Lee HY, Jang J, Park SK. Association Between Angiotensin II Receptor Blockers and the Risk of Lung Cancer Among Patients With Hypertension From the Korean National Health Insurance Service-National Health Screening Cohort. *J Prev Med Public Health*. 2020;53(6):476-486. doi:10.3961/jpmph.20.405

3. Tao MH, Chen S, Freudenheim JL, et al. Oral bisphosphonate use and lung cancer incidence among postmenopausal women. *Annals of Oncology*. 2018;29(6):1476-1485. doi:10.1093/annonc/mdy097

4. Kang J, Jeong SM, Shin DW, Cho M, Cho JH, Kim J. The Associations of Aspirin, Statins, and Metformin With Lung Cancer Risk and Related Mortality: A Time-Dependent Analysis of Population-Based Nationally Representative Data. *Journal of Thoracic Oncology*. 2021;16(1):76-88. doi:10.1016/j.jtho.2020.08.021

5. Sun JW, Zheng W, Li HL, et al. Dietary Glycemic Load, Glycemic Index, and Carbohydrate Intake on the Risk of Lung Cancer among Men and Women in Shanghai. *Nutrition and Cancer*. 2018;70(4):671-677. doi:10.1080/01635581.2018.1460675

6. Park SY, Boushey CJ, Shvetsov YB, et al. Diet Quality and Risk of Lung Cancer in the Multiethnic Cohort Study. *Nutrients*. 2021;13(5):1614. doi:10.3390/nu13051614

7. Myneni AA, Giovino GA, Millen AE, et al. Indices of Diet Quality and Risk of Lung Cancer in the Women’s Health Initiative Observational Study. *The Journal of Nutrition*. 2021;151(6):1618-1627. doi:10.1093/jn/nxab033

8. Wang Q, Hashemian M, Sepanlou SG, et al. Dietary quality using four dietary indices and lung cancer risk: the Golestan Cohort Study (GCS). *Cancer Causes Control*. 2021;32(5):493-503. doi:10.1007/s10552-021-01400-w

9. Munro HM, Yu D, Zheng W, Blot WJ, Cai Q, Shrubsole MJ. Diet quality and lung cancer incidence in a low-income population in the United States. *Br J Cancer*. 2023;129(4):626-635. doi:10.1038/s41416-023-02342-7

10. Nomura SJO, Dash C, Rosenberg L, Palmer J, Adams-Campbell LL. Fruit and VegeTable Intake and Lung Cancer Incidence Among Black Women According to Cigarette Smoking Status. *Nutrition and Cancer*. 2018;70(6):904-912. doi:10.1080/01635581.2018.1491608

11. Heath AK, Muller DC, Van Den Brandt PA, et al. Diet‐wide association study of 92 foods and nutrients and lung cancer risk in the European Prospective Investigation into Cancer and Nutrition study and the Netherlands Cohort Study. *Intl Journal of Cancer*. 2022;151(11):1935-1946. doi:10.1002/ijc.34211

12. Seow WJ, Shu XO, Nicholson JK, et al. Association of Untargeted Urinary Metabolomics and Lung Cancer Risk Among Never-Smoking Women in China. *JAMA Netw Open*. 2019;2(9):e1911970. doi:10.1001/jamanetworkopen.2019.11970

13. Li M, Cai Q, Gao YT, et al. Phytoestrogens and lung cancer risk: a nested case-control study in never-smoking Chinese women. *The American Journal of Clinical Nutrition*. 2022;115(3):643-651. doi:10.1093/ajcn/nqab358

14. Mao W, Qu J, Liu H, et al. Associations between urinary concentrations of benzothiazole, benzotriazole, and their derivatives and lung cancer: A nested case-control study. *Environmental Research*. 2024;251:118750. doi:10.1016/j.envres.2024.118750
